# Charge and Spin Transfer Dynamics in a Weakly Coupled Porphyrin Dimer

**DOI:** 10.1021/jacs.4c04186

**Published:** 2024-07-23

**Authors:** Sebastian M. Kopp, Ashley J. Redman, Igor Rončević, Lisa Schröder, Lapo Bogani, Harry L. Anderson, Christiane R. Timmel

**Affiliations:** † Centre for Advanced Electron Spin Resonance, Department of Chemistry, University of Oxford, Oxford, OX1 3QR, U.K.; ‡ Chemistry Research Laboratory, Department of Chemistry, University of Oxford, Oxford, OX1 3TA, U.K.; ¶ Department of Materials, University of Oxford, Oxford, OX1 3PH, U.K.

## Abstract

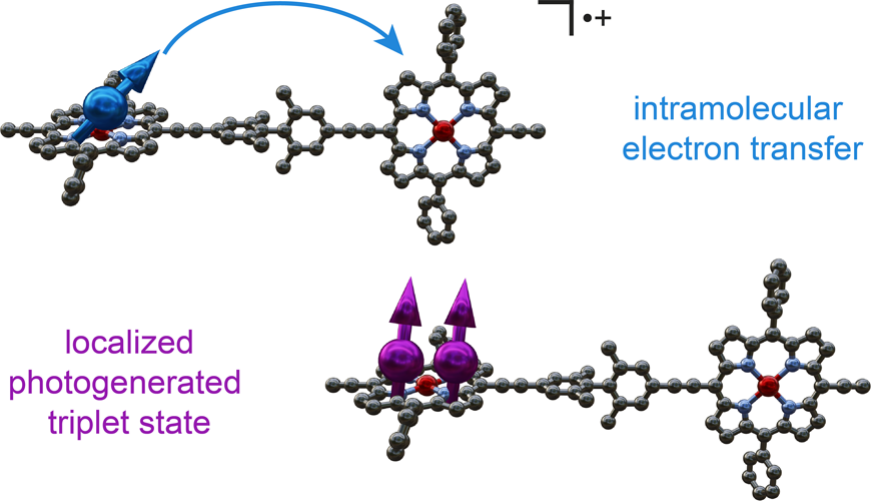

The dynamics of electron and spin transfer in the radical cation and photogenerated triplet states of a tetramethylbiphenyl-linked zinc-porphyrin dimer were investigated, so as to test the relevant parameters for the design of a single-molecule spin valve and the creation of a novel platform for the photogeneration of high-multiplicity spin states. We used a combination of multiple techniques, including variable-temperature continuous wave EPR, pulsed proton electron–nuclear double resonance (ENDOR), transient EPR, and optical spectroscopy. The conclusions are further supported by density functional theory (DFT) calculations and comparison to reference compounds. The low-temperature cw-EPR and room-temperature near-IR spectra of the dimer monocation demonstrate that the radical cation is spatially localized on one side of the dimer at any point in time, not coherently delocalized over both porphyrin units. The EPR spectra at 298 K reveal rapid hopping of the radical spin density between both sites of the dimer via reversible intramolecular electron transfer. The hyperfine interactions are modulated by electron transfer and can be quantified using ENDOR spectroscopy. This allowed simulation of the variable-temperature cw-EPR spectra with a two-site exchange model and provided information on the temperature-dependence of the electron transfer rate. The electron transfer rates range from about 10.0 MHz at 200 K to about 53.9 MHz at 298 K. The activation enthalpies Δ^‡^*H* of the electron transfer were determined as Δ^‡^*H* = 9.55 kJ mol^–1^ and Δ^‡^*H* = 5.67 kJ mol^–1^ in a 1:1:1 solvent mixture of CD_2_Cl_2_/toluene-*d*_8_/THF-*d*_8_ and in 2-methyltetrahydrofuran, respectively, consistent with a Robin–Day class II mixed valence compound. These results indicate that the interporphyrin electronic coupling in a tetramethylbiphenyl-linked porphyrin dimer is suitable for the backbone of a single-molecule spin valve. Investigation of the spin density distribution of the photogenerated triplet state of the Zn-porphyrin dimer reveals localization of the triplet spin density on a nanosecond time scale on one-half of the dimer at 20 K in 2-methyltetrahydrofuran and at 250 K in a polyvinylcarbazole film. This establishes the porphyrin dimer as a molecular platform for the formation of a localized, photogenerated triplet state on one porphyrin unit that is coupled to a second redox-active, ground-state porphyrin unit, which can be explored for the formation of high-multiplicity spin states.

## Introduction

Much of our understanding of the fundamental principles that govern electron transfer processes comes from studying organic mixed valence compounds, which consist of two or more redox centers with different formal oxidation states.^1−8^ Moreover, because of their impressive chemical tunability, organic mixed valence compounds have found application in single-molecule electronic devices,^9−12^ organic light-emitting diodes,^13,14^ near-IR dyes,^15−17^ organic superconductors^18,19^ and semiconductors,^20−22^ and organic solar cells.^23−25^

**Figure 1:**
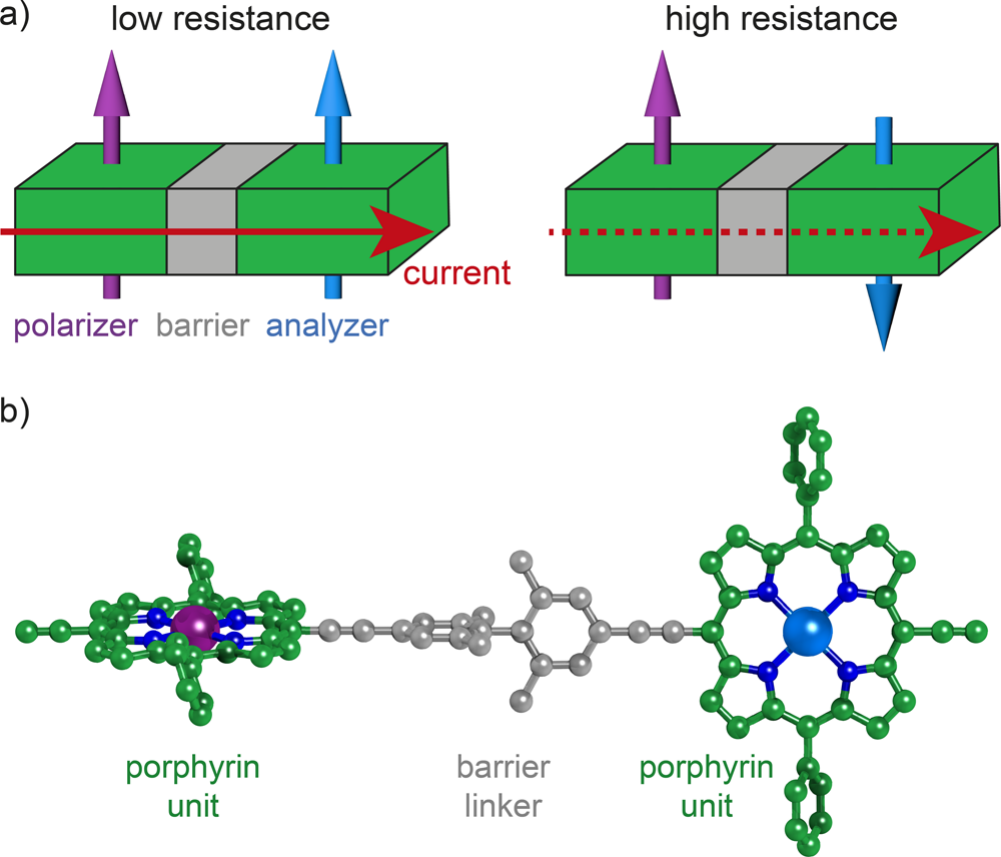
a) Scheme of the basic elements of a spin valve, which allows tuning the current (red arrow) that flows through it by the mutual alignment of two magnetic moments (purple and light blue arrows), separated by a barrier (gray). b) Scheme of the molecular unit, with metals in purple and light blue, nitrogens in blue, and carbons color-coded depending on the functional element they belong to, green for the porphyrin elements, gray for the twisted biphenyl barrier.

These applications raise important questions about how the individual building blocks that form functional mixed valence compounds can be rationally tuned to influence their performance. For example, spin valves typically require^26−31^ a high energy barrier between the functional elements (Figure 1), whereas the Pauli spin blockade observed in two-quantum-dot nanoelectronic devices^32−34^ requires low tunneling barriers. Lack of understanding of how different linking groups produce different electronic tunneling barriers impedes the rational design of molecular electronic devices, such as spin valves^26−31^ or double-dots. It is thus important to study how electronic tunneling barriers can be tuned by chemical design.

Organic mixed valence systems can be classified based on the strength of the electronic coupling between the individual redox sites as proposed by Robin and Day:^35^ (i) the electronic coupling between individual sites is negligible and results in localization of the charge (Robin–Day class I); (ii) a moderate electronic coupling results in partially localized charges and the possibility of electron transfers across a barrier between the redox sites (Robin–Day class II); (iii) the strong electronic coupling leads to coherent charge delocalization with a single minimum on the potential energy surface (Robin–Day class III).^2,35^ Due to their ability to coherently delocalize charge carriers over long distances, Robin–Day class III mixed valence compounds have been extensively investigated for applications as molecular wires.^36−41^ On the other hand, many molecular electronics elements require class II systems with reduced electronic coupling. For example, a single-molecule tunneling spin valve would need a well-controlled tunneling barrier (Figure 1).^42−44^

Our proposed prototype molecular spin valve consists of two different paramagnetic metalloporphyrin complexes that are independent single-molecule magnets at low temperature and act as spin polarizer and spin analyzer, creating and reading out a spin-polarized current, respectively (Figure 1b). The different magnetic anisotropy barriers of the paramagnetic metal porphyrin complexes allow for a selective inversion of their magnetic moments and the preferential spin polarization of the conductance electrons by an external magnetic field.^45,46^ This enables reversible switching between low-resistance and high-resistance states. If the sign of the exchange coupling between the metal ions and the conductance electrons is the same for both metalloporphyrin units, low-resistance and high-resistance states are obtained with parallel and antiparallel aligned magnetic moments of the metals, respectively (Figure 1).

The performance of a spin valve is highly dependent on the nature of the linker between the porphyrin complexes, as it must convey sufficient electronic coupling to sustain a current via single-electron transfer events, while preserving the independence of the two magnetic centers. In addition, the height of the tunneling barrier between the two spin centers determines the magnitude of the magnetoresistance.^42^ The resulting spin valve could be used as a magneto-responsive switch because its electrical resistance is expected to change in response to an external magnetic field, leading to applications as nonvolatile memory devices or for implementing logic operations in single-molecule conductance measurements.

In this work, we have used a combination of variable-temperature continuous wave (cw) EPR spectroscopy, ^1^H Mims electron–nuclear double resonance (ENDOR) spectroscopy, optical spectroscopy, and supporting density functional theory (DFT) calculations and simulations to investigate the electronic structure of a tetramethylbiphenyl-linked Zn-porphyrin dimer mixed valence compound that acts as a model system for the porphyrin backbone of the proposed spin valve. To simulate the electron transfer dynamics in this mixed valence system, we developed a robust approach using pulsed hyperfine spectroscopy to quantify interactions that cannot be resolved by cw-EPR spectroscopy. Previously, the difficulty to resolve these couplings limited the quantitative investigation of electron transfer dynamics in Zn-porphyrin nanostructures.^47−50^

The spin transfer dynamics of the radical cation mixed valence compound are compared to the dynamics of a photogenerated triplet state on the same porphyrin backbone. Molecular architectures with two decoupled porphyrin sites could be used as platforms for generating high-multiplicity spin states for quantum information processing.^51^ Recent work on photoexcited chromophore–radical or chromophore–metal systems has shed light on a broad range of highly tunable trip–doublet and quartet states with potential applications in molecular spintronics and as multilevel qudit systems.^52−57^ Therefore, a molecule that allows the formation of a photogenerated triplet state that is weakly coupled to a redox active, tunable, ground-state porphyrin unit with a metal binding site would be a versatile platform for investigating high-multiplicity spin states. In this work, we investigate the delocalization of the photogenerated triplet state in a tetramethylbiphenyl-linked Zn-porphyrin dimer using transient cw-EPR and ^1^H Mims ENDOR spectroscopy. Our findings demonstrate that the photogenerated triplet state remains localized on one porphyrin unit, on the EPR time scale, over a wide range of temperatures and in different sample environments.

## Results and Discussion

### Synthesis and Molecular Design

**Scheme 1:**
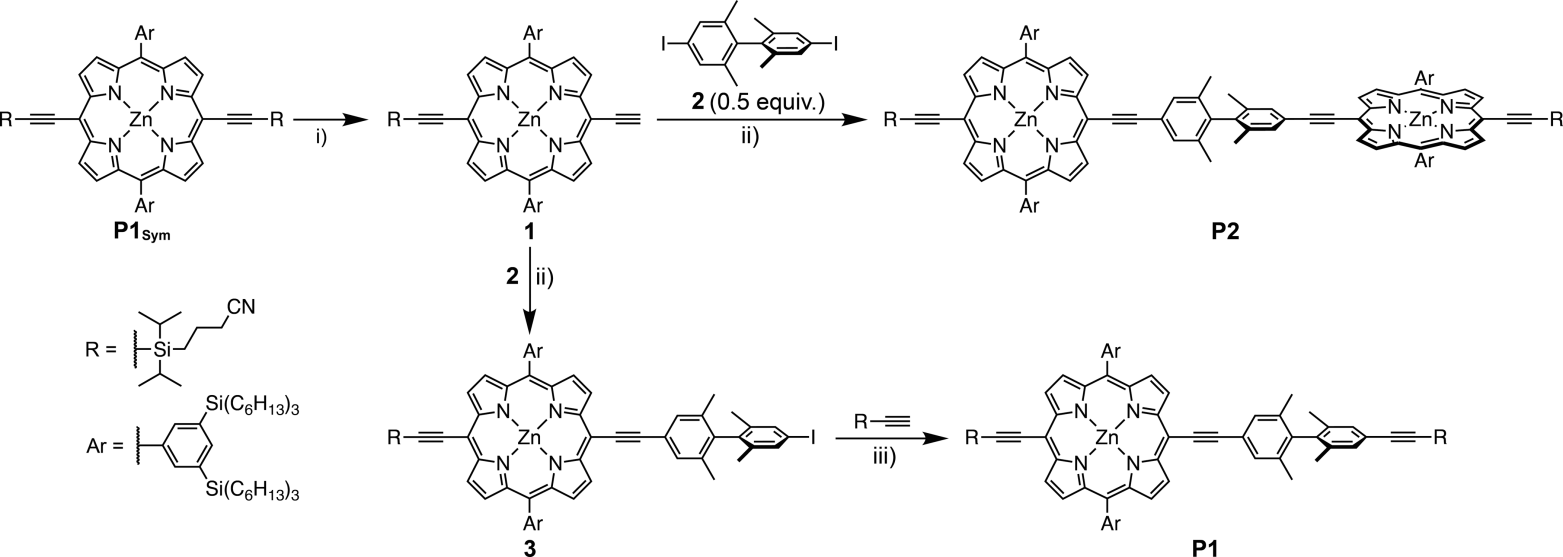
Synthesis of Biphenyl-Porphyrin Monomer **P1** and Biphenyl-Linked Porphyrin Dimer **P2** from the Symmetric Monomer **P1**_**Sym**_ Reaction conditions: (i) TBAF, CHCl_3_, 21 °C, 30 min, 50%; (ii) XPhos Pd G4, CuI, toluene/*i*-Pr_2_NH (2:1), 70 °C, 3 h, **P2**: 45%; (iii) Pd(PPh_3_)_2_Cl_2_, CuI, toluene/*i*-Pr_2_NH (5:1), 45 °C, 2 h, 15% over two steps.

Porphyrin dimer **P2** was designed to feature two redox-active zinc porphyrin units connected via a twisted tetramethyl biphenyl bridge (Scheme 1). Porphyrin monomer **P1** was chosen to model a single site of **P2**. The steric demand of the methyl substituents on the 2,2′,6,6′ positions of the biphenyl groups of **P1** and **P2** results in a perpendicular orientation of the two phenyl units. This type of twisted bridge has previously been used to hold two chromophores in a linear arrangement while avoiding direct π-conjugation.^58−60^ Investigation of the dihedral angle distribution between the porphyrin and biphenyl linker in **P1**^***•+***^ using DFT calculations shows that at 298 K about 75% of the populated geometries have an angle smaller than 25° (see Supporting Information Section 7.1 for details). Therefore, only the lowest energy geometry with a coplanar arrangement of the porphyrin and neighboring phenyl was considered in the analysis of this compound. Two factors lead to a high barrier to charge hopping in **P2**^***•+***^: a) the twist in the central biphenyl and b) the fact that the planes of the two porphyrins tend to be orthogonal. Terminal alkyne units were included in **P2** so that the dimer could be incorporated into a molecular wire. Bulky 3,5-bis(trihexylsilyl)phenyl substituents were attached to both peripheral *meso* positions of each porphyrin unit to achieve high solubility and inhibit aggregation.

Biphenyl-porphyrin monomer **P1** and biphenyl-linked porphyrin dimer **P2** were synthesized from the symmetric porphyrin monomer **P1**_**Sym**_ as summarized in Scheme 1. Monodeprotection of **P1**_**Sym**_ followed by a 2-fold Sonogashira coupling of porphyrin **1** with 4,4′-diiodo-2,2′,6,6′-tetramethyl-1,1′-biphenyl **2** and purification by recycling gel-permeation chromatography (GPC) afforded dimer **P2** in 45% yield. Sonogashira coupling of deprotected monomer **1** with 0.9 equiv. of **2** followed by recycling GPC and a second Sonogashira coupling to attach the cyanopropyldiisopropylsilyl (CPDIPS) acetylene to the biphenyl group gave biphenyl-porphyrin monomer **P1** in 15% yield over two steps. Monomer **P1**_**Sym**_ and biphenyl derivative **2** were synthesized following published procedures.^61,62^

Partial oxidation of **P1**_**Sym**_ and **P1** to their radical cations **P1**_**Sym**_^***•+***^ and **P1**^***•+***^ was achieved by addition of 0.5 equiv. of tris(4-bromophenyl)aminium hexachloroantimonate (BAHA) in a 1:1:1 solvent mixture of CD_2_Cl_2_/toluene-*d*_8_/THF-*d*_8_ or 2-methyltetrahydrofuran (MTHF), both of which form glasses at low temperatures.^36,37^ The radical cation **P2**^***•+***^ was obtained by chemical oxidation with 0.25 equiv. of BAHA to prevent oxidation of both porphyrin units in **P2**.

### UV–Visible–NIR Absorption Spectroscopy

Optical spectroscopy of an organic mixed valence system, such as **P2**^***•+***^, can provide important information about its reorganization energy, λ, and the electronic coupling, *H*, between different sites of the molecule and probes the delocalization of the radicals on a femtosecond time scale in solution.^2,8^ Compared to their neutral analogues, radical cations of π-conjugated materials typically feature two characteristic low-energy polaron absorption bands P_1_ and P_2_ that approximately correspond to HOMO → SOMO and SOMO → LUMO transitions, respectively.^63−65^

**Figure 2:**
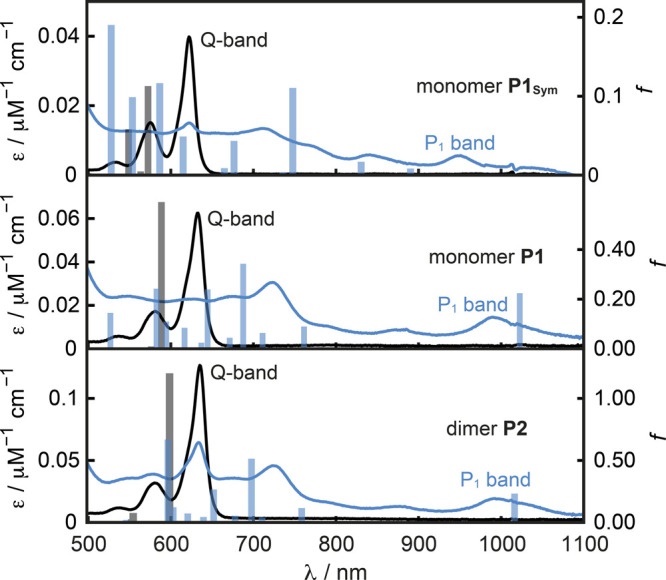
Steady-state UV–vis–NIR absorption spectra of neutral (black) and oxidized (blue) **P1**_**Sym**_, **P1**, and **P2** in CHCl_3_ (298 K). The absorption spectra of the radical cations **P1**_**Sym**_^***•+***^, **P1**^***•+***^, and **P2**^***•+***^ were obtained by oxidation with one equivalent of BAHA (see SI Figure S27 for the full oxidation titrations). The vertical bars indicate the TD-DFT (LC-ωPBE/6-31G*; ω = 0.1) calculated wavelengths and oscillator strengths, *f*, for the electronic transitions of the neutral and oxidized compounds in the absence of vibronic coupling.

The steady-state absorption spectra of neutral and oxidized **P1**_**Sym**_, **P1**, and **P2** were measured during an oxidation titration with BAHA in CHCl_3_ and are shown in Figure 2. The spectra of neutral **P1**_**Sym**_, **P1**, and **P2** show a characteristic Q-band absorption with lowest energy absorption bands at 622, 633, and 635 nm, respectively. In the spectra of their respective radical cations, the lowest energy electronic transitions are shifted to 950, 993, and 993 nm, respectively. TD-DFT calculations of the excitation energies of neutral and oxidized **P1**_**Sym**_, **P1**, and **P2** with LC-ωPBE, ω = 0.1 as functional and the 6-31G* basis set are in good agreement with the experimental observations (Figure 2, vertical bars). The calculated electronic transitions for the neutral compounds indicate that the Q-band absorptions arise from HOMO → LUMO transitions, as expected from the Gouterman four-orbital model.^66−68^ The lowest energy absorption bands of **P1**_**Sym**_^***•+***^, **P1**^***•+***^, and **P2**^***•+***^ mainly arise from HOMO → SOMO transitions and correspond to the P_1_ bands of the hole polarons. The slight bathochromic shift of the Q-band absorption of neutral **P1** compared to **P1**_**Sym**_ results from an extension of the conjugated π-system by one phenyl group in the biphenyl-porphyrin systems. The lack of a further red-shift of the lowest-energy absorption band of **P2** is evidence for the electronic decoupling of the two porphyrin sites by the twisted biphenyl linker. Similarly for the radical cations, the P_1_ bands of **P1**^***•+***^ and **P2**^***•+***^ are shifted toward longer absorption wavelengths by about 40 nm (Δ*E*_Abs_ = 0.06 eV) relative to the P_1_ band of **P1**_**Sym**_^***•+***^, which points toward a partial delocalization of the radical cation onto one-half of the biphenyl linker. The identical lowest-energy absorption bands of **P1**^***•+***^ and **P2**^***•+***^ stem from the same radical distribution in both systems and demonstrate the localization of the hole on one site of **P2**^***•+***^. TD-DFT calculations on **P2**^***•+***^ predict a very weak (*f* = 0.0006) intervalence charge transfer (IV-CT) band at 2032 nm, and observation of this band could provide a more in-depth analysis of the electronic structure; however, this band appears to be too broad and weak to be observed experimentally (SI Figure S29 and Table S15).

### Continuous Wave EPR

**Figure 3:**
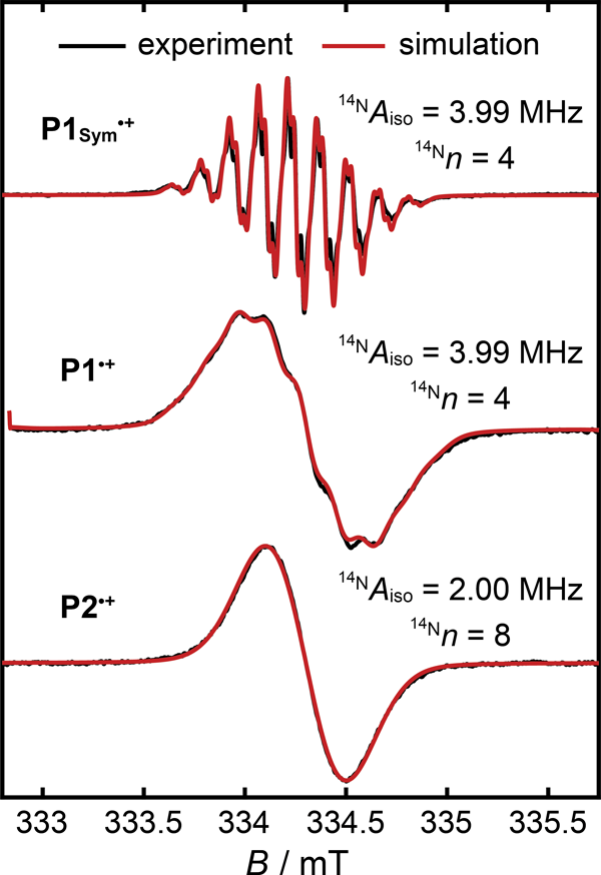
Experimental cw-EPR spectra of the radical cations **P1**_**Sym**_^***•+***^, **P1**^***•+***^, and **P2**^***•+***^ (black) acquired at 298 K in CD_2_Cl_2_/toluene-*d*_8_/THF-*d*_8_ 1:1:1 at X-band frequencies. The simulated cw-EPR spectra (red) of **P1**_**Sym**_^***•+***^ and **P1**^***•+***^ were obtained by least-squares fitting of the isotropic ^14^N hyperfine interactions ^14N^*A*_iso_, and in the case of **P1**_**Sym**_^***•+***^ the isotropic hyperfine coupling ^1H^*A*_iso_ to four equivalent ^1^H nuclei using EasySpin.^70^ Dimer **P2**^***•+***^ was simulated assuming a complete and uniform distribution of the radical spin density over both sites on the EPR time scale with ^14N^*A*_iso_(**P2**^***•+***^) = 0.5·^14*N*^*A*_iso_(**P1**^***•+***^).

The cw-EPR spectra of the radical cations **P1**_**Sym**_^***•+***^, **P1**^***•+***^, and **P2**^***•+***^ at 298 K in CD_2_Cl_2_/toluene-*d*_8_/THF-*d*_8_ 1:1:1 at X-band frequencies are shown in Figure 3. The spectrum of **P1**_**Sym**_^***•+***^ is consistent with previous reports for this radical cation^37^ and consists of nine prominent hyperfine lines that arise from an isotropic coupling of ^14N^*A*_iso_ = 3.99 MHz to the four equivalent ^14^N nuclei, which are further split by an isotropic coupling of ^1H^*A*_iso_ = 0.94 MHz to the four equivalent *ortho* protons of the *meso* aryl groups. The cw-EPR spectrum of **P1**^***•+***^ exhibits a partially resolved hyperfine structure that arises from an isotropic coupling of ^14N^*A*_iso_ = 3.99 MHz to the four equivalent ^14^N nuclei. The ^1^H hyperfine coupling pattern is not resolved in this spectrum due to additional hyperfine interactions, which leads to the inhomogeneous broadening of the spectrum. The room-temperature cw-EPR spectrum of **P2**^***•+***^ lacks a resolved hyperfine structure and is narrower than the spectrum of **P1**^***•+***^. If hyperfine interactions are the main contribution to the spectral envelope and the radical spin density is completely and uniformly distributed over both sites of **P2**^***•+***^ on the EPR time scale, the theoretical relationship in eqs 1 and 2 established by Norris et al. applies:^69^
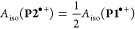
1
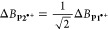
2where  and  are the Gaussian spectral envelope widths of **P1**^***•+***^ and **P2**^***•+***^, respectively.

The numerical simulation of the cw-EPR spectrum of **P2**^***•+***^ following eq 1 with Gaussian and Lorentzian peak-to-peak line width contributions Γ_G_ = 0.14 mT and Γ_L_ = 0.05 mT, identical to those in **P1**^***•+***^, is in excellent agreement with the experimental spectrum (Figure 3). The trend in the spectral envelope widths of **P1**^***•+***^ and **P2**^***•+***^ is analyzed in Figure S3 of the Supporting Information and shows a good agreement with the Norris relationship in eq 2. This implies fast hopping of the radical spin density over both sites of **P2**^***•+***^ on the EPR time scale at 298 K. The lack of a bathochromic shift in the NIR absorption spectrum of **P2**^***•+***^ compared to **P1**^***•+***^ shows that the apparent spin density distribution is the result of fast reversible intramolecular electron transfers and not coherent delocalization. Similar behavior was found for the cw-EPR spectra of **P1**^***•+***^ and **P2**^***•+***^ in MTHF at 298 K discussed in Section 3.2 of the Supporting Information

**Figure 4:**
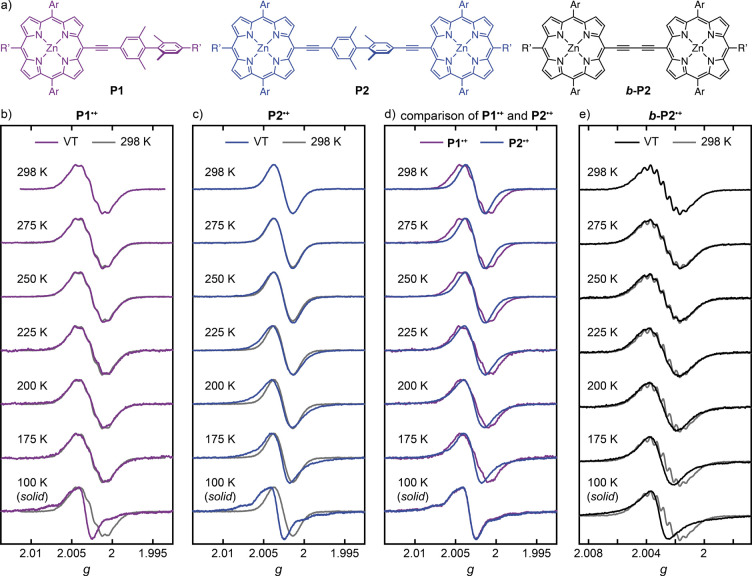
a) Chemical structures of **P1**, **P2**, and ***b*****-P2** with colors corresponding to the cw-EPR spectra in b)–e); Ar = 3,5-bis(trihexylsilyl)phenyl and R′ = cyanopropyldiisopropylsilyl (CPDIPS) acetylene. Variable-temperature (VT) cw-EPR spectra of b) **P1**^***•+***^, c) **P2**^***•+***^, and e) ***b*****-P2**^***•+***^ in CD_2_Cl_2_/toluene-*d*_8_/THF-*d*_8_ 1:1:1 between 298 and 175 K in fluid solution and at 100 K in a frozen glass at X-band frequencies. At each temperature, the variable-temperature spectrum is compared to the corresponding spectrum at 298 K (gray). The variable temperature spectra of **P2**^***•+***^ are superimposed in Figure S7 to highlight the exchange broadening. d) Comparison of the variable-temperature cw-EPR spectra of **P1**^***•+***^ (purple) and **P2**^***•+***^ (blue).

Dynamic chemical processes such as electron transfers result in line width changes of the cw-EPR transitions and provide information about the rate of the chemical process from the magnitude of the exchange broadening.^71−74^ To obtain insights into the kinetics of reversible intramolecular electron transfer in **P2**^***•+***^, the temperature dependence of the cw-EPR spectra of **P1**^***•+***^ and **P2**^***•+***^ in CD_2_Cl_2_/toluene-*d*_8_/THF-*d*_8_ 1:1:1 at X-band frequencies was investigated by variable-temperature cw-EPR spectroscopy between 298 and 175 K in fluid solution and at 100 K in a frozen glass (Figure 4).

The variable-temperature cw-EPR spectra of the butadiyne-linked porphyrin dimer cation ***b*****-P2**^***•+***^ were measured as a reference to differentiate between dynamic line shape effects that result from the intramolecular electron transfer in **P2**^***•+***^ and anisotropic broadening due to slower tumbling at low temperatures (Figure 4e). The size and shape of ***b*-P2** is comparable to **P2**, and the rotational diffusion times of both molecules are expected to be similar under identical conditions. The investigations of ***b*****-P2**^***•+***^ by Peeks, Tait, et al.^36^ demonstrated coherent delocalization of the radical spin density over both porphyrin units (Robin–Day class III). Therefore, temperature-dependent line width changes in the cw-EPR spectrum of ***b*****-P2**^***•+***^ arise from anisotropic broadening and indicate insufficient rotational averaging of ***b*****-P2**^***•+***^ and **P2**^***•+***^.

The fluid-phase variable-temperature cw-EPR spectra of **P1**^***•+***^ between 298 and 175 K are virtually identical, indicating that the hyperfine interactions and the intrinsic Lorentzian line widths, Γ_L_, in **P1**^***•+***^ are essentially temperature-independent above 175 K. In contrast, the fluid-phase cw-EPR spectra of **P2**^***•+***^ exhibit a continuous broadening of their spectral envelope with decreasing temperature, which is highlighted by the superimposed variable-temperature cw-EPR spectra of **P2**^***•+***^ in Figure S7. The cw-EPR spectra of ***b*****-P2**^***•+***^ are virtually unchanged between 298 and 225 K but show features consistent with anisotropic broadening at 200 and 175 K. Consequently, the broadening of the cw-EPR spectra of **P2**^***•+***^ between 298 and 225 K can be attributed to a slower rate *k*_ex_ of the intramolecular electron transfer, whereas at 200 and 175 K exchange broadening and anisotropic broadening both contribute to the line shape. In all fluid-phase measurements in CD_2_Cl_2_/toluene-*d*_8_/THF-*d*_8_ 1:1:1, the spectral envelope of **P2**^***•+***^ remains narrower than that of **P1**^***•+***^, which suggests that the intramolecular electron transfer in **P2**^***•+***^ remains fast on the EPR time scale. The cw-EPR spectra of **P1**^***•+***^ and **P2**^***•+***^ in a frozen glass show anisotropic broadening and are temperature-independent between 150 and 100 K (Figure 4d and SI Figure S5). The identical spectra of **P1**^***•+***^ and **P2**^***•+***^ at 100 K demonstrate the localization of the radical electron on one porphyrin unit in **P2**^***•+***^ on the EPR time scale, which is supported by the simulation of these cw-EPR spectra (SI Figure S8). This localization is probably caused by a combination of the reorganization energy associated with electron transfer, the lack of flexibility of the porphyrin backbone in the frozen solution, and possibly localization of the SbCl_6_^–^ counterion. In contrast, the frozen solution cw-EPR spectral envelope of ***b*****-P2**^***•+***^ remains narrower than the spectral envelope of **P1**_**Sym**_^***•+***^, consistent with coherent delocalization of the radical spin density over both porphyrin units in the dimer.^36^

The variable-temperature cw-EPR spectra of **P1**^***•+***^, **P2**^***•+***^, and ***b*****-P2**^***•+***^ in MTHF are discussed in detail in Section 3.3 of the Supporting Information Similar to the discussion above, the cw-EPR spectra of **P1**^***•+***^ and ***b*****-P2**^***•+***^ are virtually temperature-independent between 298 and 225 K, while the cw-EPR spectra of **P2**^***•+***^ exhibit increasing exchange broadening with decreasing temperature. All fluid-phase cw-EPR spectra exhibit increasing anisotropic broadening between 200 and 140 K and suggest localization of the radical spin density on one porphyrin unit of **P2**^***•+***^ on the EPR time scale below 200 K (SI Figure S6). The temperature at which hopping becomes slow in MTHF (200 K) is higher than in CD_2_Cl_2_/toluene-*d*_8_/THF-*d*_8_ 1:1:1 (175 K), due to the increasing viscosity of MTHF^75^ and possibly its higher polarity.

### ^1^H Mims ENDOR Spectroscopy

**Figure 5:**
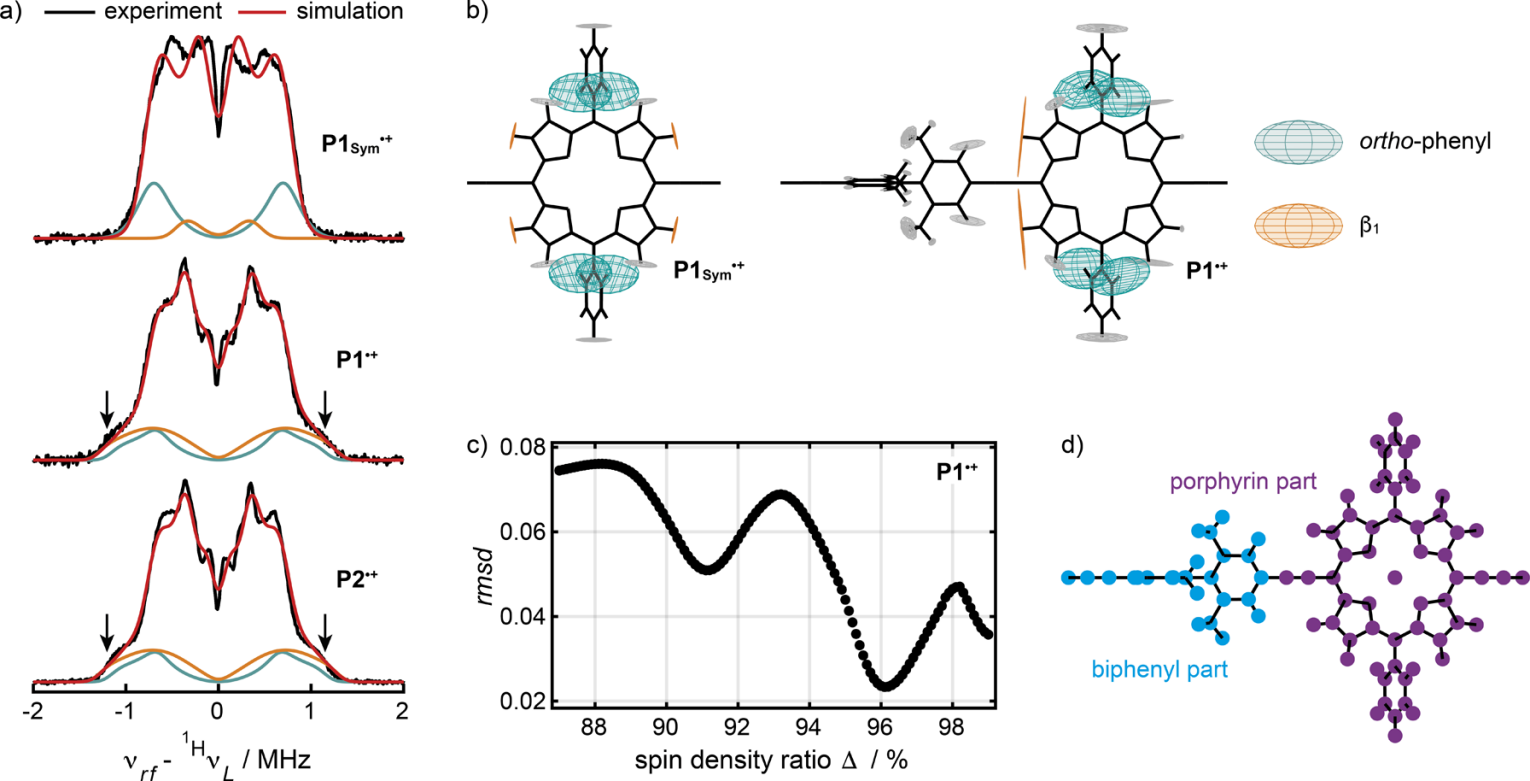
a) Experimental (black) and simulated (red) ^1^H Mims ENDOR spectra of **P1**_**Sym**_^***•+***^, **P1**^***•+***^, and **P2**^***•+***^ recorded at Q-band frequencies in frozen CD_2_Cl_2_/toluene-*d*_8_/THF-*d*_8_ 1:1:1 at 80 K. The spectrum of **P1**_**Sym**_^***•+***^ was simulated using anisotropic ^1^H hyperfine tensors, ^1H^***A***, obtained from DFT calculations at the B3LYP/EPR-II level of theory. The spectra of **P1**^***•+***^ and **P2**^***•+***^ were simulated under identical conditions using hyperfine tensors calculated via the distributed point-dipole model for **P1**^***•+***^ with a spin density ratio Δ = 96.1%, which is defined as the fraction of spin density on the porphyrin part. The individual contributions to the ^1^H ENDOR spectra from the *ortho*- and β_1_-hydrogens are highlighted in dark green and orange, respectively. b) Schematic representation of the anisotropic ^1^H hyperfine tensors of **P1**_**Sym**_^***•+***^ and **P1**^***•+***^. The tensors of the *ortho*- and β_1_-protons that give rise to the highlighted transitions in a) are shown in dark green and orange, respectively; additional tensors are shown in gray as a reference. c) Root-mean-square deviation (rmsd) between the experimental and simulated ^1^H Mims ENDOR spectra of **P1**^***•+***^ as a function of Δ. d) Schematic visualization of the formal separation of **P1**^***•+***^ into a porphyrin (purple) and biphenyl (blue) fragment.

The anisotropic ^1^H hyperfine interactions of **P1**_**Sym**_^***•+***^, **P1**^***•+***^, and **P2**^***•+***^ were measured by pulse Mims ENDOR spectroscopy in frozen CD_2_Cl_2_/toluene-*d*_8_/THF-*d*_8_ 1:1:1 at 80 K and Q-band frequencies (Figure 5). Although decomposition of the ENDOR spectra is not feasible due to the large number of nuclei with similar hyperfine interactions, these spectra provide valuable insight into the spin delocalization of the radical cations. In addition, the anisotropic hyperfine interactions are not dynamically averaged in the frozen solvent matrix. Simulation of the ENDOR spectra can therefore be used to obtain information about the ^1^H hyperfine interactions that are not resolved due to the inhomogeneous broadening of the room-temperature cw-EPR spectra. In addition, dynamic processes such as polaron migration and electron hopping can occur rapidly on the EPR time scale at room temperature and can contribute to the investigated spin density distribution.^47^ However, these processes are typically not observed by EPR spectroscopy at cryogenic temperatures in a frozen matrix, which provides a better probe for the instantaneous radical distribution and hyperfine interactions.^36,37^

The Q-band ^1^H Mims ENDOR spectra of **P1**_**Sym**_^***•+***^, **P1**^***•+***^, and **P2**^***•+***^ are centered around the proton Larmor frequency, ^1H^ν_L_, and split into symmetric peaks by the hyperfine interactions (Figure 5a). This is characteristic of nuclei in the weak coupling limit, |^^1^H^*A*| < |2^^1^H^ν_L_|. The ENDOR spectra of **P1**^***•+***^ and **P2**^***•+***^ are virtually identical, which suggests that both radical cations have the same anisotropic proton hyperfine interactions. This is consistent with localization of the radical spin density on one porphyrin unit in **P2**^***•+***^ on the EPR time scale and provides additional evidence that the radical cation is not coherently delocalized over both porphyrin units. Simulation of the ENDOR spectra of **P1**^***•+***^ and **P2**^***•+***^ using DFT-calculated hyperfine tensors is not feasible due to an over-delocalization of the radical spin density from the porphyrin onto the biphenyl part for a wide a range of DFT functionals with different range-separation parameters (SI Figure S9).

To overcome this limitation, we employed the distributed point-dipole model, described in Section 4 of the Supporting Information, to calculate the ^1^H hyperfine interactions in **P1**^***•+***^ from a systematically assigned spin density distribution. In brief, **P1**^***•+***^ was formally divided into a porphyrin and a biphenyl subunit (Figure 5d). The extent of radical delocalization between the subunits is quantified by the spin density ratio, Δ, which is defined as the fraction of spin density on the porphyrin subunit. The spin density assigned to each subunit is distributed to individual nuclei in agreement with DFT calculations. Variation of the spin density ratio, Δ, allows for the systematic screening of the radical delocalization with a single fitting parameter. The optimal spin density ratio to describe **P1**^***•+***^ was determined by comparing the experimental ^1^H Mims ENDOR spectrum of **P1**^***•+***^ with a range of simulated spectra using hyperfine tensors, ^1H^***A***, calculated via the distributed point dipole model for a series of spin density ratios (SI Figure S12). The best agreement between the experimental and simulated spectra was found for Δ = 96.1%, as judged by the root-mean-square deviation (rmsd) between the two spectra (Figure 5c).

The ^1^H Mims ENDOR spectra of **P1**^***•+***^ and **P2**^***•+***^ were simulated with identical hyperfine tensors, ^1H^***A***, obtained for a spin density ratio Δ = 96.1% (Figure 5a). Noticeably, both ENDOR spectra exhibit a resolved shoulder corresponding to a hyperfine interaction of approximately 2.7 MHz that is absent in the spectrum of **P1**_**Sym**_^***•+***^ (Figure 5a; indicated by arrows). The origin of this feature becomes evident from the simulations of the individual hyperfine contributions to the ENDOR spectra (Figure 5a,b). For **P1**_**Sym**_^***•+***^, the width of the ENDOR spectrum is determined by the hyperfine interactions to the *ortho*-protons of the aryl side groups (dark green). Introduction of the biphenyl unit in **P1**^***•+***^ desymmetrizes the porphyrin and changes the environment around the porphyrin β-protons. This results in a substantial increase of the largest principal component of the anisotropic hyperfine interactions with the β_1_-protons (orange) that determine the width of the ENDOR spectra of **P1**^***•+***^ and **P2**^***•+***^ and give rise to the pronounced shoulder. Apart from subtle intensity changes, identical ^1^H ENDOR spectra of **P1**^***•+***^ and **P2**^***•+***^ are observed in frozen MTHF at 80 K (SI Figure S13). This is important evidence that the hyperfine couplings of both cations are independent of the solvent systems in frozen solution.

### Simulation of the Electron Transfer Dynamics

**Figure 6:**
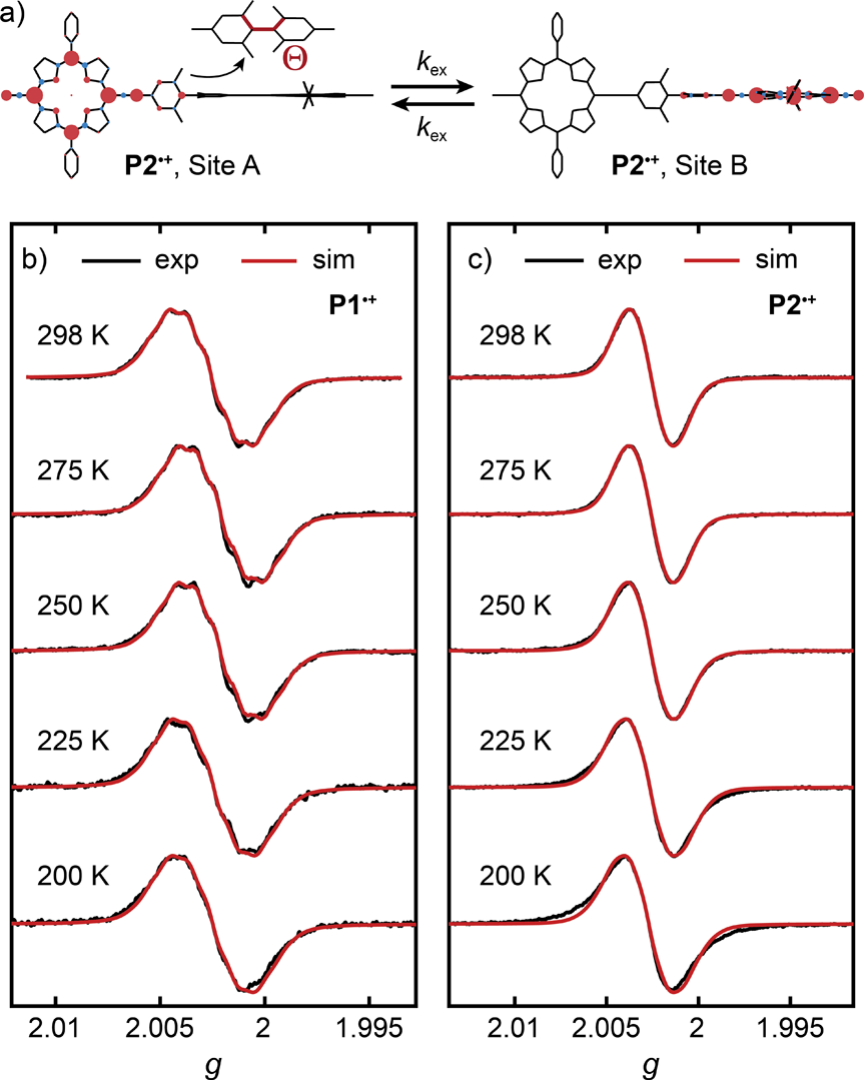
a) Schematic visualization of the redistribution of spin density during the reversible intramolecular electron transfer in **P2**^***•+***^. The spin population on each nucleus is represented by a sphere centered on the atom with a radius proportional to the magnitude of its assigned spin population. Red spheres represent excess spin-up and light-blue spheres indicate excess spin-down populations. The torsion angle Θ between the phenyl parts of the tetramethylbiphenyl linker is shown in the inset. Comparison of the experimental (black) and simulated (red) variable-temperature cw-EPR spectra of b) **P1**^***•+***^ and c) **P2**^***•+***^ at X-band frequencies in CD_2_Cl_2_/toluene-*d*_8_/THF-*d*_8_ 1:1:1 between 298 and 200 K. Simulations were performed with a two-site chemical exchange model. All monomer spectra were simulated in the slow exchange limit with *k*_ex_ = 10^–10^ MHz.

The temperature dependence of the rate constant, *k*_ex_, for reversible intramolecular electron transfer in **P2**^***•+***^ was determined by simulating the variable-temperature cw-EPR spectra of **P1**^***•+***^ and **P2**^***•+***^ between 298 and 200 K at X-band frequencies. The best-fit simulations in CD_2_Cl_2_/toluene-*d*_8_/THF-*d*_8_ 1:1:1 are shown in Figure 6. The simulations of **P2**^***•+***^ were obtained with the electron transfer rates, *k*_ex_, in Table 1 with error margins determined as the electron transfer rates with a 5% larger rmsd between the experimental and simulated spectra than the minimum rmsd (SI Figure S18). The experimental and simulated variable-temperature cw-EPR spectra in MTHF are compared in Figure S19. All simulations were performed with a two-site chemical exchange model implemented by Stoll^76^ and Kozhanov^77^ in MATLAB based on the EasySpin^70^ software package in which the radical spin density is distributed over one-half or the other half of **P2**^***•+***^ (Figure 6a). The magnetic interactions of the unpaired electron with the two redox centers of **P2**^***•+***^ involved in the intramolecular electron transfer are analogous to the magnetic interactions in **P1**^***•+***^ and need to be characterized in detail for an accurate simulation of the variable-temperature spectra. The inherent peak-to-peak line width, Γ, of the EPR transitions at each temperature was determined by simulating the cw-EPR spectra of **P1**^***•+***^ using the chemical exchange model in the slow exchange limit (*k*_ex_ = 10^–10^ MHz) and subsequently used to describe the two redox sites in the simulation of the variable-temperature cw-EPR spectra of **P2**^***•+***^ at the same temperature.

**Table 1:** Summary of the Electron Transfer Rates *k*_ex_ Resulting in the Best-Fit Simulation of the Variable-Temperature cw-EPR Spectra of **P2**^***•+***^ in CD_2_Cl_2_/Toluene-*d*_8_/THF-*d*_8_ 1:1:1^a^

**P2**^***•+***^	298 K	275 K	250 K	225 K	200 K
*k*_ex_/MHz	53.9_–6.5_^+8.0^	40.7_–3.8_^+4.4^	23.1_–2.4_^+2.8^	12.0_–2.1_^+2.4^	10.0_–3.2_^+4.1^
rmsd	0.0110	0.0105	0.0142	0.0240	0.0440

a The uncertainties of *k*_ex_ were determined as the electron transfer rates resulting in root-mean-square deviations (rmsd) 5% larger than the minimum rmsd. The intensity of the experimental and simulated spectra was normalized to calculate the rmsd.

The characteristic exchange broadening of the cw-EPR spectra of **P2**^***•+***^ for different electron transfer rates *k*_ex_ is caused by dynamic changes of the hyperfine interactions. Therefore, simulation of the exchange process requires accurate knowledge of the magnitude of the isotropic hyperfine interaction that are modulated during electron transfer. While the isotopic ^14^N hyperfine interactions, ^14N^*A*_iso_, can be obtained from least-squares fitting of the cw-EPR spectrum of **P1**^***•+***^ at 298 K, the proton hyperfine couplings are not resolved in the cw-EPR spectra (*vide supra*). Instead, the ^1^H hyperfine interactions were probed by Mims ENDOR spectroscopy and accurately simulated with ^1^H hyperfine tensors, ^1H^***A***, calculated with the distributed point dipole model for Δ = 96.1%. The five largest isotropic components of these hyperfine tensors in combination with ^14N^*A*_iso_ allow an accurate description of the hyperfine interactions modulated during the electron transfer process (SI Figure S16, Table S3). Attempts to simulate the variable-temperature cw-EPR spectra of **P1**^***•+***^ and **P2**^***•+***^ using only ^14N^*A*_iso_ do not result in a good agreement with the experimental spectra and highlight the importance of the unresolved ^1^H hyperfine interactions (see SI Section 6.5).

The reversible intramolecular electron transfer in **P2**^***•+***^ results in a modulation of the hyperfine interactions, which means that hyperfine couplings to the nuclei on one-half of **P2**^***•+***^ are accompanied by the absence of couplings to the corresponding nuclei on the other side, and vice versa. Consequently, the symmetry-related nuclei on both sides of **P2**^***•+***^ are dynamically equivalent with identical time-averaged hyperfine interactions but different instantaneous hyperfine couplings. The effects of a modulation of hyperfine interactions for dynamically equivalent nuclei on the EPR spectrum of a simple model system are discussed in Section 6.2 of the Supporting Information

**Figure 7:**
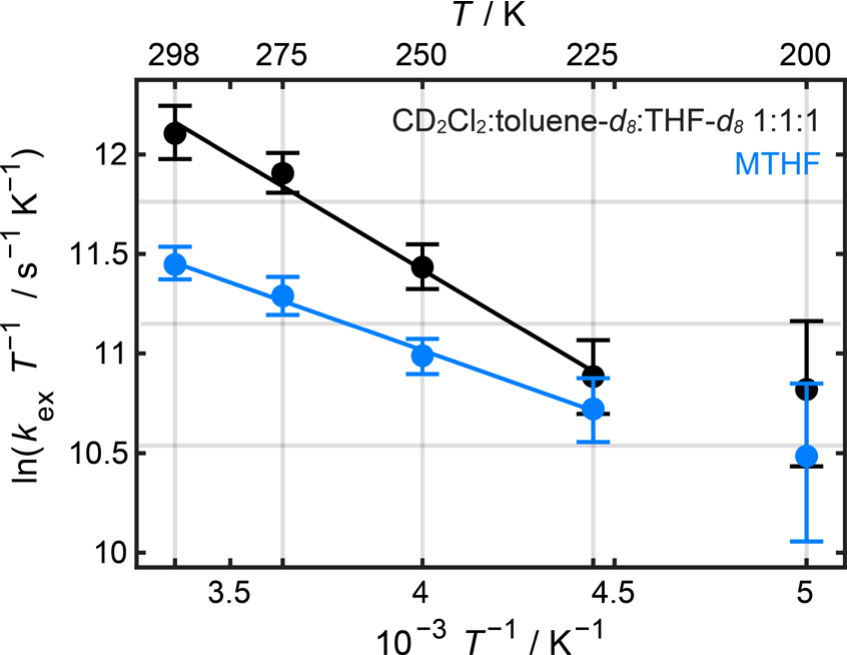
Eyring plots of ln(*k*_ex_*T*^–1^) versus *T*^–1^ for **P2**^***•+***^ in CD_2_Cl_2_/toluene-*d*_8_/THF-*d*_8_ 1:1:1 (black) and MTHF (blue). The data points at 200 K were omitted from the linear regression analysis because the experimental cw-EPR spectra at this temperature exhibit anisotropic broadening.

The simulations of the variable-temperature cw-EPR spectra of **P2**^***•+***^ in CD_2_Cl_2_/toluene-*d*_8_/THF-*d*_8_ 1:1:1 and MTHF quantify the decreasing rate of electron transfer with decreasing temperature. The spectra of **P2**^***•+***^ between 298 and 225 K can be well simulated with the two-site exchange model, whereas the spectra at 200 K, with contributions of anisotropic broadening to the spectral shape, are no longer well described by the simulations, as evident from the larger root-mean-square deviations and uncertainty boundaries. Therefore, we focus on the temperature range above 200 K in the following analysis. The temperature-dependence of the electron transfer rate *k*_ex_ between 298 and 225 K in CD_2_Cl_2_/toluene-*d*_8_/THF-*d*_8_ 1:1:1 and MTHF was investigated using the Eyring relationship in eq 3 to determine the activation enthalpy, Δ^‡^*H,* and activation entropy, Δ^‡^*S,* that govern the reversible intramolecular electron transfer in **P2**^***•+***^:
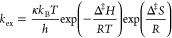
3where *k*_B_ is the Boltzmann constant, *R* is the molar gas constant, *h* is Planck’s constant, and κ is the electron transmission coefficient.^78,79^ Plots of ln(*k*_ex_*T*^–1^) versus *T*^–1^ for **P2**^***•+***^ in CD_2_Cl_2_/toluene-*d*_8_/THF-*d*_8_ 1:1:1 and MTHF are shown in Figure 7 and were used to determine Δ^‡^*H* and Δ^‡^*S* from the slope and intercept of the trendline between 298 and 225 K, respectively. The kinetic parameters that govern the electron transfer in **P2**^***•+***^ are given in Table 2.

**Table 2:** Summary of the Activation Enthalpies Δ^‡^*H* and Entropies Δ^‡^*S* Calculated with the Eyring Equation with κ = 0.035 from the Temperature Dependence of the Electron Transfer Rate *k*_ex_ in **P2**^***•+***^ in CD_2_Cl_2_/Toluene-*d*_8_/THF-*d*_8_ 1:1:1 and MTHF

	Δ^‡^*H*/kJ mol^–1^	Δ^‡^*S*/kJ mol^–1^
CD_2_Cl_2_/toluene-*d*_8_/THF-*d*_8_ 1:1:1	9.55 ± 0.43	–36.5 ± 0.55
2-methyltetrahydrofuran	5.67 ± 0.55	–55.4 ± 1.30

We used theoretical modeling to estimate the value of the electron transmission coefficient κ. The rate of the electron transfer in **P2**^***•+***^ is determined by the overlap integral *S*_AB_ between sites A and B of the two-state chemical exchange process (Figure 6a). The expectation value of the torsion angle Θ between the two phenyl groups of the 2,2′,6,6′-tetramethylbiphenyl linker of **P2**^***•+***^ and the resulting overlap integral, *S*_AB_, were calculated by DFT between 298 and 200 K to investigate the temperature dependence of the electronic coupling between sites A and B (see Supporting Information Section 7.2 for detailed information). In the equilibrium geometry, the two phenyl groups are virtually orthogonal (Θ_eq_ = 90°), resulting in a low overlap integral between the two symmetric charge-localized states. The magnitude of *S*_AB_ is modulated by changes in the torsion angle, Θ: a broader range of Θ becomes available with increasing temperature, which increases *k*_ex_. The extent of coupling between the electron transfer and nuclear vibrations, ν_n_, was quantified by calculating the electron transmission coefficient following the Landau–Zener approach, which gave κ = 0.035.^80,81^ The small transmission coefficient (κ ≪ 1) strongly indicates that the electron transfer in **P2**^***•+***^ occurs in the nonadiabatic regime and proceeds slower than the nuclear motions (κ_ex_ < ν_n_).

As expected from Marcus–Hush theory, the electron transfer dynamics in **P2**^***•+***^ are energetically and dynamically influenced by the polarity of the solvent, resulting in different activation enthalpies and entropies in CD_2_Cl_2_/toluene-*d*_8_/THF-*d*_8_ 1:1:1 and MTHF.^1,82,83^ Solvation effects contribute to the outer-sphere reorganization energy, λ_o_, which is often calculated using a dielectric continuum model based on the static dielectric constant, ϵ, and the square of the refractive index, *n*, of the solvent.^1,84^ The implicit temperature dependence of λ_o_ and therefore Δ^‡^*H* due to the temperature dependence of ϵ and *n* is omitted in our analysis.^85^ As seen from Table 2, the activation enthalpies, Δ^‡^*H*, of the intramolecular electron transfer in **P2**^***•+***^ in CD_2_Cl_2_/toluene-*d*_8_/THF-*d*_8_ 1:1:1 and MTHF are of the same order of magnitude. The larger activation enthalpy in CD_2_Cl_2_/toluene-*d*_8_/THF-*d*_8_ 1:1:1 compared to MTHF suggests a higher outer reorganization energy in the mixed solvent system. Further analysis of the solvent effects on the electron transfer dynamics in **P2**^***•+***^ would require the in-depth characterization of the dielectric properties of the 1:1:1 solvent mixture of CD_2_Cl_2_/toluene-*d*_8_/THF-*d*_8_ and is beyond the scope of this work. The activation entropies, Δ^‡^*S*, were calculated for a transmission coefficient κ = 0.035, which approximates the dependence of the intramolecular electron transfer on nuclear vibrations by considering only the change in the biphenyl torsion angle. The negative sign of Δ^‡^*S* suggests that an ordered transition state of **P2**^***•+***^ with sufficient electronic coupling between the porphyrin units is required for an efficient electron transfer.

### Investigation of the Triplet-State Delocalization

**Figure 8:**
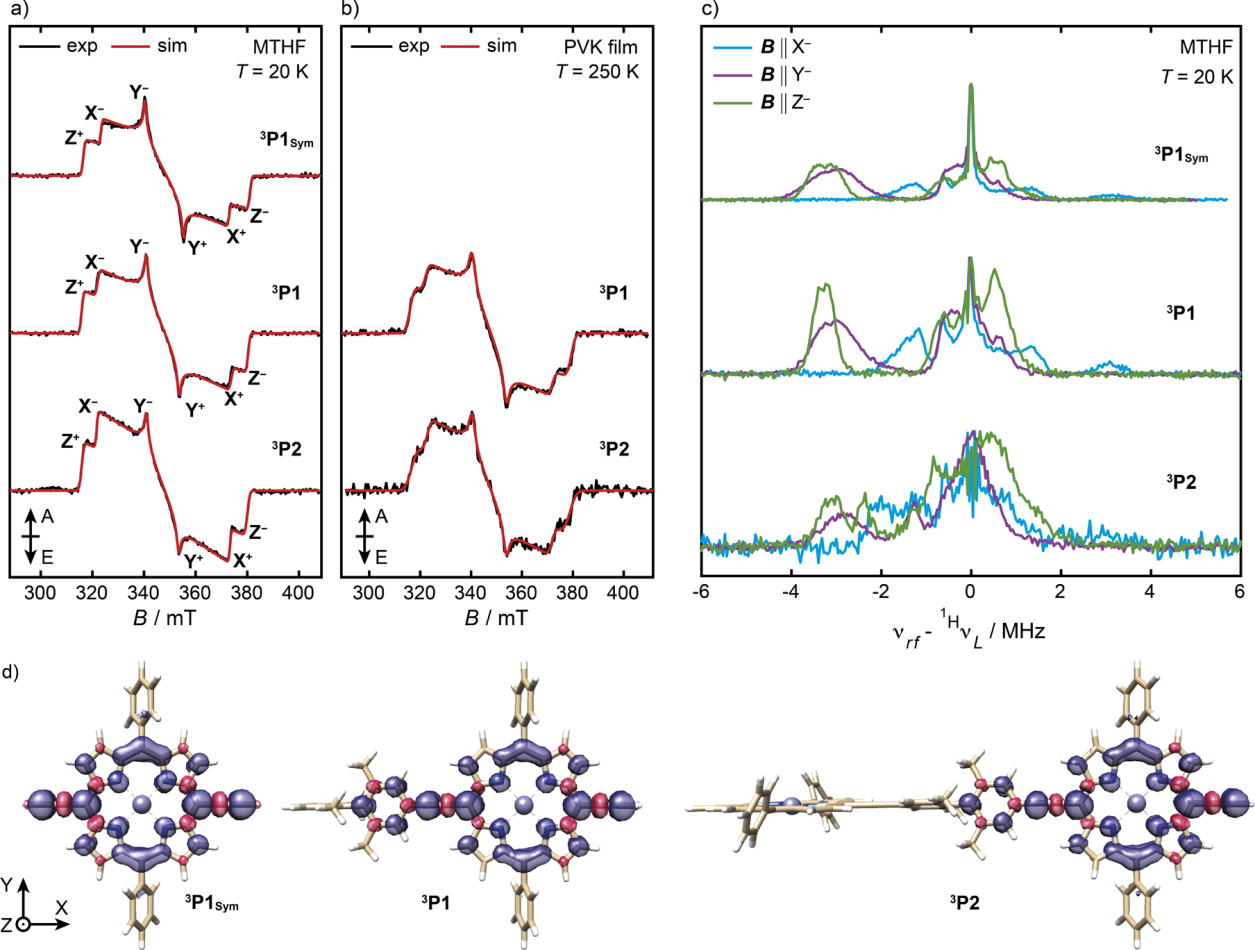
Comparison of the experimental (black) and simulated (red) transient EPR spectra of ^3^**P1**_**Sym**_, ^3^**P1**, and ^3^**P2** at X-band frequencies in a) MTHF at 20 K and b) a polyvinylcarbazole (PVK) film at 250 K. The experimental spectra are recorded as an average between 300 and 400 ns after the laser pulse with depolarized light excitation at 532 nm. Simulations were performed with the parameters reported in Table 3. The energetic ordering of the principal components of ***D*** was chosen as |*D*_*Z*_| > |*D*_*X*_| > |*D*_*Y*_|, and the canonical field positions are indicated (A = absorption, E = emission). c) Experimental ^1^H Mims ENDOR spectra of ^3^**P1**_**Sym**_, ^3^**P1**, and ^3^**P2** recorded at the *X*^–^, *Y*^–^, and *Z*^–^ field positions at 20 K in MTHF at X-band frequencies. d) DFT-calculated spin density distributions of ^3^**P1**_**Sym**_, ^3^**P1**, and ^3^**P2** using the B3LYP functional and the EPR-II basis set.

The transfer of triplet excitation is a closely related process to electron transfer, since they both involve electron exchange interaction.^86−89^ As part of this study, we therefore also investigate the triplet excited state of the porphyrin dimer **P2**. The transient EPR spectra of ^3^**P1**_**Sym**_, ^3^**P1**, and ^3^**P2** in MTHF at 20 K and for ^3^**P1**, and ^3^**P2** in a polyvinylcarbazole (PVK) film at 250 K were averaged between 300 and 400 ns after the laser pulse with depolarized light excitation at 532 nm and are shown in Figure 8. The spin polarization of all transient EPR spectra does not change significantly over time (SI Figure S32). The *AAAEEE* polarization pattern (*A* = absorptive, *E* = emissive) of the transient EPR spectra indicates a non-Boltzmann population of the triplet sublevels as expected for photogenerated porphyrin triplet states.^90,91^ This spin polarization arises from different relative intersystem crossing rates to and relaxation rates from the individual triplet sublevels.

The line shapes and widths of the EPR spectra of organic triplets are dominated by their zero-field splitting (ZFS) interactions that arise from the dipolar spin–spin interaction between the two electrons that comprise the triplet and in some cases also from a spin–orbit interaction. Recent work by Moise, Redman, and co-workers has shown that spin–orbit contributions to the zero-field splitting are negligible in Zn-porphyrins akin to the ones investigated here, and we therefore focus our discussion exclusively on the spin–spin interaction.^92^ The spin–spin contributions to the zero-field splitting ***D***-tensor can be defined by two ZFS parameters, *D* and *E*,
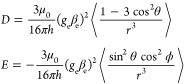
4where *r* is the interspin distance, θ is the angle between the *z*-axis of the molecule and the spin–spin vector, ϕ is the azimuthal angle of the spin–spin vector in the *xy*-plane, *g*_e_ is the electronic *g*-factor, β_e_ is the Bohr magneton, μ_0_ is the vacuum permeability, and the angular brackets indicate integration over the triplet wave function.^93,94^ The magnitudes of *D* and *E* are measures of the interspin distance, *r*, and the orthorhombicity of the spin density distribution, respectively, and determine the distance between the turning points in the transient EPR spectra. These turning points correspond to the canonical orientations of ***D*** denoted as *X*, *Y*, and *Z* in Figure 8; the + and – subscripts refer to the *m*_*s*_ = 0 ↔ *m*_*s*_ = +1 and *m*_*s*_ = −1 ↔ *m*_*s*_ = 0 transitions, respectively. All other things being equal, an increasing delocalization of the triplet state should result in a reduction of |*D*|.^91^ The sign of the *D*-parameter indicates the orientation of the ZFS tensor in the molecular frame: a positive *D*-value suggest an oblate spin density (*Z*-axis of the ***D***-tensor is perpendicular to the porphyrin plane), whereas a negative *D*-value is consistent with a prolate spin density (*Z*-axis of ***D*** is in the porphyrin plane).

The transient EPR spectrum of ^3^**P1**_**Sym**_ in MTHF at 20 K is consistent with previous investigations of the triplet state of this compound by Tait et al.^95,96^ The intersystem crossing in Zn-porphyrin monomers is driven by spin–orbit coupling due to mixing of the zinc d-orbitals with the π-system of the porphyrin and results in a preferential population of the out-of-plane sublevel of the triplet state.^97^ For ^3^**P1**_**Sym**_, this results in a preferential population of the *Z* sublevel (Table 3). Tait et al. assigned a positive sign to the *D*-parameter of ^3^**P1**_**Sym**_ using magnetophotoselection experiments, which is replicated by DFT calculations at the B3LYP/EPR-II level of theory (Table 4).^95^ The *D*-values of biphenyl porphyrins ^3^**P1** and ^3^**P2** are similar to |*D*| for ^3^**P1**_**Sym**_. This suggests a similar triplet-state delocalization in all three systems consistent with a localization of the triplet state on one-half of ^3^**P2**; in other words, there is no evidence for exciton mobility on the EPR time scale.

**Table 3:** Zero-Field Splitting Parameters and Relative Sublevel Populations of ^3^**P1**_**Sym**_, ^3^**P1**, and ^3^**P2** Determined through Simulation of Their Transient EPR Spectra Presented in Figure 8

	2-methyltetrahydrofuran (20 K)			PVK film (250 K)		
	|*D*|/MHz	|*E*|/MHz	*p*_*X*_:*p*_*Y*_:*p*_*Z*_	|*D*|/MHz	|*E*|/MHz	*p*_*X*_:*p*_*Y*_:*p*_*Z*_
^3^**P1**_**Sym**_	903 ± 2	163 ± 2	0.05:0.00:0.95			
^3^**P1**	904 ± 2	182 ± 2	0.07:0:0.93	891 ± 4	170 ± 2	0.10:0:0.90
^3^**P2**	900 ± 2	183 ± 1	0:0.05:0.95	875 ± 6	166 ± 2	0.04:0:0.96

**Table 4:** Experimental and DFT-Calculated Zero-Field Splitting Parameters for ^3^**P1**_**Sym**_, ^3^**P1**, and ^3^**P2**

	experiment		B3LYP/EPR-II	
	*D*/MHz	|*E*|/MHz	*D*/MHz	|*E*|/MHz
^3^**P1**_**Sym**_	+903 ± 2	163 ± 2	+501	122
^3^**P1**	+904 ± 2	182 ± 2	+492	135
^3^**P2**	+900 ± 2	183 ± 1	+481	133

The 12% increase of |*E*| for ^3^**P1** and ^3^**P2** compared to the value for ^3^**P1**_**Sym**_ points toward a higher orthorhombicity of the spin-density distribution. The preferential population of the *Z* sublevels in ^3^**P1** and ^3^**P2** and the position of the hyperfine transitions in the Mims ENDOR spectra (*vide infra*) suggest that the ***D***-tensors of ^3^**P1** and ^3^**P2** have approximately the same orientation as in ^3^**P1**_**Sym**_, resulting in a positive *D*-parameter. The experimental *D*-values are qualitatively replicated by DFT calculations with B3LYP as functional and the EPR-II basis set that predict approximately identical magnitudes and a positive sign for the *D*-parameters of ^3^**P1**_**Sym**_, ^3^**P1**, and ^3^**P2** (Table 4).

The discrepancy between the DFT-calculated spin–spin contributions to the *D*-parameter and the experimental values is a known limitation for aromatic triplet states.^98,99^ The trend in DFT-calculated *D*-parameters for a series of compounds can however inform the interpretation of their experimental spectra.^91,98,99^ The spin density distributions found in these calculations show a partial delocalization of the triplet spin density onto the biphenyl units of ^3^**P1** and ^3^**P2** but no triplet delocalization over both sites of ^3^**P2** as expected from the experimental data. This localization is consistent with previous reports of localized triplet states in porphyrin nanostructures with a near-orthogonal arrangement of the neighboring porphyrin units at 20 K in MTHF.^100,101^ To investigate whether the triplet delocalization in ^3^**P2** can be increased at higher temperatures, the transient EPR spectra of ^3^**P1** and ^3^**P2** were measured in a PVK film at 250 K (Figure 8b). The approximately identical *D*-parameters and spectral widths of ^3^**P1** and ^3^**P2** suggest that the triplet state remains localized on one porphyrin unit in ^3^**P2**. The smaller values of |*D*| in the PVK film compared to MTHF could be the result of slightly different equilibrium geometries in the two environments. The absence of an increasing triplet state delocalization in ^3^**P2** probably results from a combination of the limited structural flexibility of the porphyrin dimer in the solid film environment and the intrinsically strong coupling between the two electrons comprising the exciton state; in other words, the reorganization energy is too high.

The extent of triplet-state delocalization in ^3^**P1**_**Sym**_, ^3^**P1**, and ^3^**P2** was further probed using proton hyperfine interactions measured by orientation-selective Mims ENDOR spectroscopy at the canonical field positions *X*^–^, *Y*^–^, and *Z*^–^ at 20 K in MTHF (Figure 8c). Previous studies of ^3^**P1**_**Sym**_ revealed that the largest hyperfine interactions are observed for the β_1_ protons closest to alkyne bonds, consistent with DFT calculations (SI Figure S37).^95,96^ The transition selection (*m*_*s*_ = 0 ↔ *m*_*s*_ = +1 or *m*_*s*_ = −1 ↔ *m*_*s*_ = 0) during the ^1^H ENDOR measurements provides information about the relative sign of the hyperfine interaction and the ZFS *D*-value. In the weak coupling regime, the triplet ENDOR spectrum is asymmetric around the Larmor frequency: in addition to a peak at the Larmor frequency from the *T*_0_ state, for a positive *D*-value, a second peak is observed at ^1H^ν_*L*_ + *A*_*i*_ and ^1H^ν_*L*_ – *A*_*i*_ for the *T*_0_ ↔ *T*_–_ and *T*_0_ ↔ *T*_+_ transitions, respectively.^91^ The sign of the β_1_ hyperfine interactions in ^3^**P1**_**Sym**_ is negative following the assignment by Tait et al.^95^ This is in agreement with DFT-calculated hyperfine tensors at the B3LYP/EPR-II level of theory that also predict negative hyperfine interactions to the β_1_ and *ortho*-biphenyl nuclei in ^3^**P1** and ^3^**P2**.

The observation of the prominent hyperfine interactions in ^3^**P1**_**Sym**_, ^3^**P1**, and ^3^**P2** at rf-frequencies smaller than ^1H^ν_L_ at negative canonical positions is consistent with a positive *D*-value in all investigated systems. Simulations of the ^1^H ENDOR spectra of ^3^**P1**_**Sym**_, ^3^**P1**, and ^3^**P2** using the DFT-calculated hyperfine tensors are broadly in agreement with the experimental spectra, although the magnitude of the hyperfine interactions with nuclei on the biphenyl linker is strongly exaggerated (SI Figure S37). This suggests an over-delocalization of triplet spin density onto the biphenyl linker by DFT, analogous to the over-delocalization of the radical cation spin density (*vide supra*). The lack of a substantial reduction of the largest hyperfine tensors between ^3^**P1** and ^3^**P2** is additional evidence for the localization of the radical spin density on one-half of ^3^**P2** (SI Figure S36, Table S17).

## Conclusions

The electron and spin distribution of the radical cation and photogenerated triplet states of the biphenyl-linked porphyrin dimer **P2** were investigated by a combination of EPR and optical spectroscopy, DFT calculations, and theoretical modeling. Fluid-phase variable-temperature cw-EPR spectroscopy of **P2**^***•+***^ in CD_2_Cl_2_/toluene-*d*_8_/THF-*d*_8_ 1:1:1 and MTHF between 298 and 225 K reveals a thermally activated, intramolecular electron transfer between the two degenerate sites of **P2**^***•+***^, consistent with a Robin–Day class II mixed valence compound in the nonadiabatic regime. We introduced a robust approach to quantify hyperfine interactions that cannot be resolved by cw-EPR spectroscopy, but are nevertheless crucial to simulate the electron transfer dynamics in **P2**^***•+***^, by investigating the ^1^H hyperfine interactions of reference compound **P1**^***•+***^ by ^1^H Mims ENDOR spectroscopy and supporting simulations. This enables the quantification of the electron transfer dynamics in porphyrin nanostructures by fitting the variable-temperature cw-EPR spectra to a two-site exchange model, which was previously only possible by artificially introducing nuclei with large hyperfine interactions.^48−50^ The kinetic parameters that govern the intramolecular electron transfer in **P2**^***•+***^ were quantified by investigating the temperature dependence of the electron transfer rate, *k*_ex_. The activation enthalpies, Δ^‡^*H*, that govern the electron transfer in **P2**^***•+***^ highlight the presence of a large barrier between the porphyrin sites, which is promising for achieving high tunneling magnetoresistance ratios in a molecular spin valve.^42^ At the same time, the observation of intramolecular electron transfer between the two sites of **P2**^***•+***^ in CD_2_Cl_2_/toluene-*d*_8_/THF-*d*_8_ 1:1:1 and MTHF suggests that the electronic coupling remains sufficient to sustain an electric current. This makes **P2** an excellent candidate for the backbone of a single-molecule spin valve. Future work will focus on the design and synthesis of a heterobimetallic analogue of **P2** as a molecular spin valve, by inserting paramagnetic lanthanide cations into the porphyrins and investigating the molecular conductance as a function of magnetic field.

Investigation of the spin density distribution of the photogenerated triplet state of **P2** using transient EPR spectroscopy and ^1^H Mims ENDOR spectroscopy at 20 K in MTHF confirms the localization of the triplet excitons on one-half of the dimer. This localization is preserved at 250 K in a PVK film, confirming that the orthogonal arrangement of the porphyrin units in **P2** prevents the delocalization of the triplet state over a wide range of temperatures and in different sample environments. This makes **P2** a versatile platform for the exploration of high-multiplicity spin states by introducing a π-radical or paramagnetic metal center such as Cu(II) on the porphyrin unit adjacent to the photogenerated triplet state.

Our results demonstrate that chemical modifications of the electronic coupling in porphyrin nanostructures can be used to control the spin distribution of organic radicals and photogenerated triplet states in a solid matrix: butadiyne-linked porphyrins dimers with strong interporphyrin electronic coupling exhibit coherent delocalization of their radical cations and triplet states in a frozen solution,^36,95,96^ whereas the weakly coupled biphenyl-linked porphyrin dimer **P2** localizes doublet and triplet spin densities on one porphyrin unit under analogous conditions. Porphyrin nanostructures with weak electronic coupling between individual porphyrin units fulfill important design criteria for novel spintronic materials and are promising as molecular platforms for quantum information processing.

## Supporting Information Available

DFT Cartesian coordinates (ZIP)
Details on synthetic procedures, spectroscopy, theoretical calculations, data analysis and simulation methods, additional UV–vis–NIR, fluorescence, and EPR spectra, NMR and mass spectra of novel compounds (PDF)
