# Supplementary material for: Charge and Spin Transfer Dynamics in a Weakly Coupled Porphyrin Dimer
Source: J Am Chem Soc. 2024 Jul 23;146(31):21476–89. doi: 10.1021/jacs.4c04186 (PMC11311228; doi:10.1021/jacs.4c04186)
Supplement: Supplementary file 2 — ja4c04186_si_002.pdf [file ja4c04186_si_002.pdf]

Supporting Information for

Charge and spin transfer dynamics in a weakly coupled porphyrin dimer

Sebastian M. Kopp, Ashley J. Redman, Igor Rončević, Lisa Schröder, Lapo Bogani, Harry L. Anderson, and Christiane R. Timmel

Centre for Advanced Electron Spin Resonance, Department of Chemistry, University of Oxford, Oxford OX1 3QR, UK
Chemistry Research Laboratory, Department of Chemistry, University of Oxford, Oxford OX1 3TA, UK
Department of Material, University of Oxford, OX1 3PH, Oxford, UK

Correspondence to: harry.anderson@chem.ox.ac.uk; christiane.timmel@chem.ox.ac.uk.

Table of Contents

1. General Methods **2**
2. Synthetic Procedures **3**
3. cw-EPR Spectroscopy **5**
4. Distributed Point-Dipole Model for Calculating Hyperfine Interactions **11**
5. ^{1}H Mims ENDOR Spectroscopy **15**
6. Simulation of the Electron Transfer Dynamics **17**
7. DFT Modelling **29**
8. Absorption Spectroscopy and TD-DFT Calculations **32**
9. Transient EPR of Photogenerated Triplet States **41**
10. NMR and Mass Spectra of Novel Compounds **45**
11. References **54**

1. General Methods

1.1 Synthesis and Purification

All reagents were purchased as reagent grade and used without further purification. Solvents for column chromatography were HPLC grade. Dry toluene and diisopropylamine (*i*-Pr_2NH) were obtained from the solvent drying system MBraun MBSPS-5-BenchTop under nitrogen atmosphere (H_2O content < 20 ppm as determined by Karl-Fischer titration). Flash column chromatography was carried out using SiO_2 (60 Å, 230–400 mesh) under positive pressure. Analytical thin-layer chromatography was carried out on aluminum-backed silica gel 60 F254 plate. Evaporation of solvents was performed at 25–80 °C and 10–900 mbar. Semi-preparative gel-permeation chromatography (GPC) was carried out on a Shimadzu recycling GPC system equipped with a LC-20 AD pump, SPD-20A UV detector and a set either of JAIGEL 3H (20 × 600 mm) and JAIGEL 4H (20 × 600 mm) columns using toluene/pyridine (99:1) as eluent at a flow rate of 3.5 mL/min. Reported yields refer to spectroscopically and chromatographically pure compounds that were dried under high vacuum (0.01–0.005 mbar) before analytical characterization.

1.2 NMR Spectroscopy

NMR spectra were recorded in $CDCl_3$ at 298 K using a Bruker AVIII HD 500 spectrometer. ^1H NMR chemical shifts are reported in ppm, calibrated to residual $CHCl_3$ at 7.26 ppm. Coupling constants are given in Hertz (Hz), to the nearest 0.1 Hz. Multiplicity (s = singlet, d = doublet, t = triplet, q = quartet, m = multiplet) and coupling constants were reported whenever possible. ^1H NMR signals were assigned based on comparison between compounds, chemical shifts, integrals, coupling constants and 2D NMR spectroscopy.

1.3 Mass Spectrometry

MALDI-ToF mass spectra were measured using a Bruker MALDI Autoflex Speed spectrometer. *Trans*-2-[3-(4-*tert*-butylphenyl)-2-methyl-2-propenylidene]-malononitrile (DCTB) was employed as matrix.

1.4 Optical Spectroscopy

UV-vis-NIR absorbance measurement was recorded with a Perkin-Elmer Lambda 20 spectrophotometer or a Jasco V770 spectrophotometer using an Infrasil® Quartz 1 cm cuvette. Measurements were carried out at 298 K under ambient conditions.

1.5 Chemical Oxidation

Samples of $\mathbf{P1_{Sym}}^{\bullet+}$, $\mathbf{P1}^{\bullet+}$, and $\mathbf{P2}^{\bullet+}$ for EPR measurements were prepared in CD_2Cl_2:toluene-d_8:THF-d_8 1:1:1 or 2-methyltetrahydrofuran and degassed using three freeze-pump-thaw cycles. For chemical oxidation, ~0.5 equiv. of tris-(4-bromophenyl)ammoniumyl hexachloroantimonate (BAHA) (for $\mathbf{P1_{Sym}}$ and **P1**) or ~0.25 equiv. of BAHA (for **P2**) (2.0 mM in CD_2Cl_2) were added to a solution of the porphyrin oligomer (~200mM in CD_2Cl_2:toluene-d_8:THF-d_8 1:1:1 or 2-methyltetrahydrofuran) under argon just before the EPR experiments. All radical cation samples were prepared directly either in X-band EPR tubes (3.8 mm o.d.) or Q-band EPR tubes (2.0 mm o.d.). For low-temperature measurements the samples were then frozen in liquid nitrogen.

1.6 DFT Calculations

All DFT calculations for this work were performed using the ORCA software package.[1] The relevant computational details for the calculation of the molecular properties are given in the relevant sections of this supplemental information. Geometry optimization of the $\mathbf{P1_{Sym}}$, **P1**, **P2**, $\mathbf{P1_{Sym}}^{\bullet+}$, $\mathbf{P1}^{\bullet+}$, $\mathbf{P2}^{\bullet+}$, $^3\mathbf{P1_{Sym}}$, $^3\mathbf{P1}$, and $^3\mathbf{P2}$ structures were performed with B3LYP as functional and the 6-31G* basis set. The stationary nature of the converge geometries was confirmed by the absence of imaginary frequencies in the DFT calculated vibration spectra.

2. Synthetic Procedures

Porphyrin monomers **P1**$_{\textbf{Sym}}$[2] and **1**[2], dimer ***b*-P2**[2], and biphenyl linker **2**[3] were synthesized as reported previously (Figure S1).

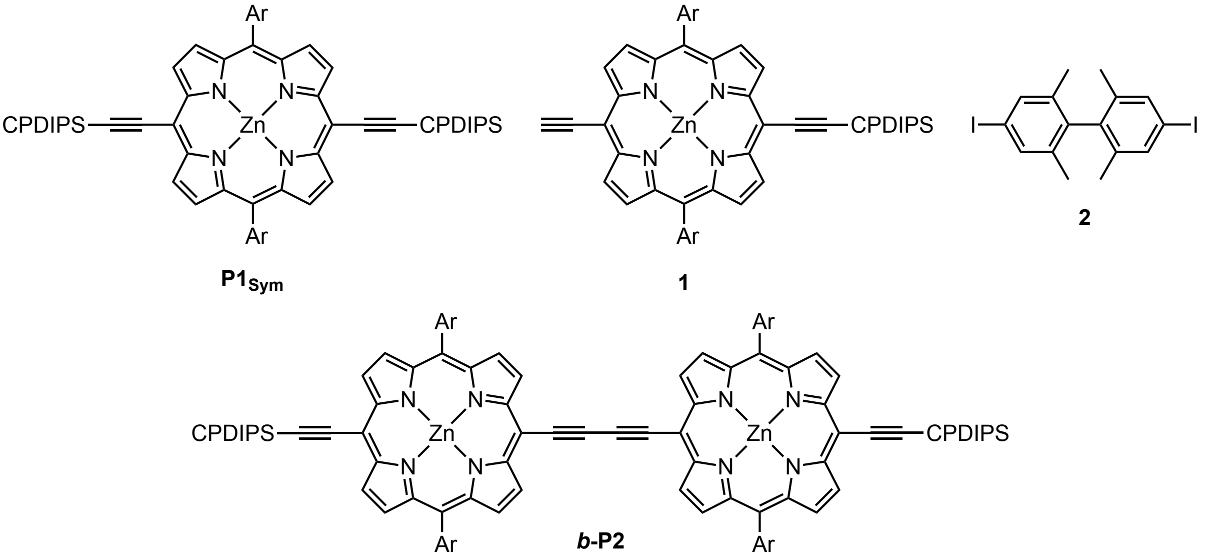

Figure S1. Compounds prepared following literature reported procedures.

3:

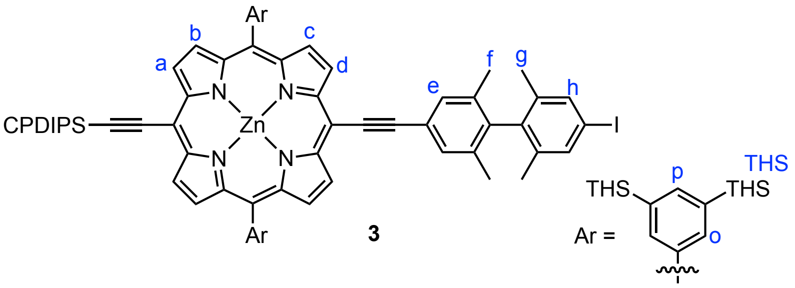

In a Schlenk flask, porphyrin monomer **1** (100 mg, 1 equiv., 53 µmol) and 4,4'-diiodo-2,2',6,6'-tetramethyl-1,1'-biphenyl **2** (22.1 mg, 0.9 equiv., 47.7 µmol) were dissolved in dry toluene (10 mL) and *i*-Pr_2NH (3 mL) and subjected to two freeze-pump-thaw cycles. While frozen, CuI (0.2 mg, 1 µmol) and XPhos Pd G4 (2.74 mg, 3.2 µmol) were added from a stock solution in toluene followed by three additional freeze-pump-thaw cycles. After reacting for 3 hours at 45 °C, the reaction mixture was cooled to room temperature, passed through a short pad of silica ($CHCl_3$) and a size-exclusion column (toluene + 1% pyridine) and purified by recycling GPC (toluene + 1% pyridine) to afford the mono-coupled product **3** (41.4 mg) in sufficient purity.

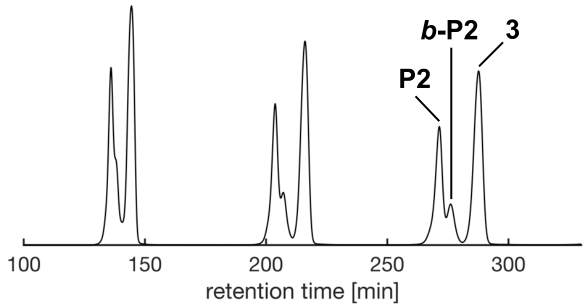

Figure S2. Part of the recycling GPC trace (toluene + 1% pyridine, λ = 570 nm) for the synthesis of **3**.

^{1}H NMR (500 MHz, $CDCl_3$, 298K) δ_H = 9.82 (d, J = 4.6 Hz, 2H, a), 9.69 (d, J = 4.5 Hz, 2H, d), 8.94 (d, J = 4.5 Hz, 2H, c), 8.93 (d, J = 4.7 Hz, 2H, b), 8.26 (s, 4H, o), 8.00 (s, 2H, p), 7.81 (s, 2H, e), 7.57 (s, 2H, h), 2.55 (t, J = 6.8 Hz, 2H,CPDIPS-CH_2), 2.22–2.17 (m, 2H,CPDIPS-CH_2), 2.05 (s, 6H,f), 1.97 (s, 6H,g), 1.57–0.79 (m, 170H, THS + CPDIPS-CH + CPDIPS-CH_3), 1.18–1.15 (m, 2H, CPDIPS-CH_2) ppm.

P1:

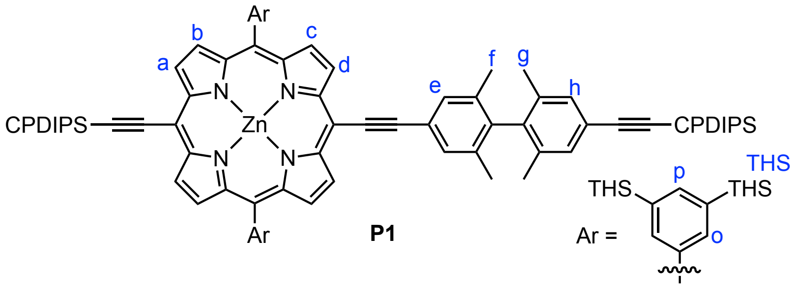

Without further purification, **3** (35.8 mg, 1 equiv., 16.1 µmol), CuI (614 µg, 3.2 µmol), and $Pd(PPh_3)_2Cl_2$ (2.3 mg, 3.2 µmol) were dissolved in dry toluene (2 mL) and *i*-Pr_2NH (0.4 mL) and subjected to three freeze-pump-thaw cycles before cyanopropyldiisopropylsilylacetylene (13.4 mg, 0.01 mL, 4 equiv., 64.5 µmol) was added by syringe. The reaction mixture was stirred at 45 °C for 2 hour and passed through a silica pad ($CHCl_3$). The product was purified by flash column chromatography (20 % CH_2Cl_2/PE to 40% CH_2Cl_2/PE) to afford **P1** (23.4 mg, 10.2 µmol, 63 %) as a green solid.

^{1}H NMR (500 MHz, $CDCl_3$, 298K) δ_H = 9.83 (d, J = 4.6 Hz, 2H, a), 9.69 (d, J = 4.6 Hz, 2H, d), 8.94 (d, J = 4.6 Hz, 2H, c), 8.93 (d, J = 4.6 Hz, 2H, b), 8.26 (s, 4H, o), 8.00 (s, 2H, p), 7.81 (s, 2H, e), 7.34 (s, 2H, h), 2.55 (t, J = 6.8 Hz, 2H,CPDIPS-CH_2), 2.47 (t, J = 7.0 Hz, 2H,CPDIPS-CH_2), 2.23–2.17 (m, 2H, CPDIPS-CH_2), 2.05 (s, 6H, f), 2.00 (s, 6H, g), 1.96–1.89 (m, 2H, CPDIPS-CH_2), 1.50–0.88 (m, 188H, THS + CPDIPS-CH + CPDIPS-CH_3 + CPDIPS-CH_2) ppm. **MALDI-ToF** *m/z* 2298.840 (calculated for $[C_{146}H_{226}N_6Si_6Zn]^+$ = $[$**P1**$]^+$: 2299.313). **UV-vis** ($CDCl_3$) *l* [nm] (ε [μM^{-1} cm^{-1}]): 633 (0.063), 581 (0.017), 538 (0.005), 444 (0.458).

P2:

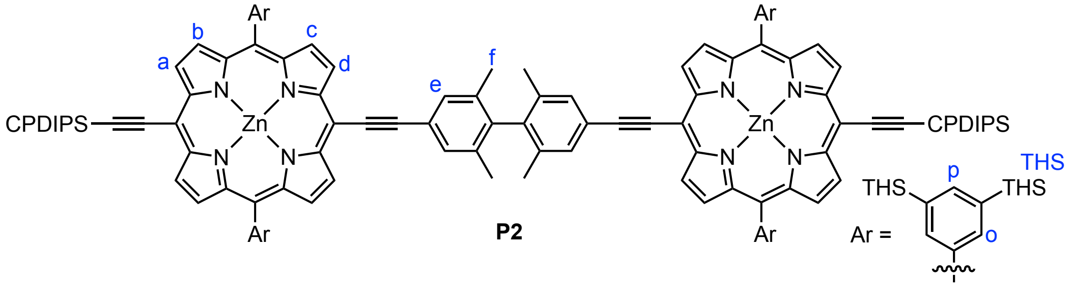

In a Schlenk flask, porphyrin monomer **1** (76 mg, 2 equiv., 40.3 µmol) and 4,4'-diiodo-2,2',6,6'-tetramethyl-1,1'-biphenyl **2** (9.34 mg, 1 equiv., 20.2 µmol) were dissolved in dry toluene (8 mL) and *i*-Pr_2NH (3 mL) and subjected to two freeze-pump-thaw cycles. While frozen, CuI (0.12 mg, 0.6 µmol) and XPhos Pd G4 (2.00 mg, 2.3 µmol) were added from a stock solution in toluene followed by three additional freeze-pump-thaw cycles. After reacting for 4 hours at 45 °C, the reaction mixture was cooled to room temperature, passed through a short pad of silica ($CHCl_3$), and purified by flash column chromatography (20 % CH_2Cl_2/PE to 30% CH_2Cl_2/PE) to afford **P2** (36.0 mg, 9.1 µmol, 45 %) as a green solid.

^{1}H NMR (500 MHz, $CDCl_3$, 298 K) δ_H = 9.87 (d, J = 4.6 Hz, 4H, a), 9.70 (d, J = 4.6 Hz, 4H, d), 8.95 (d, J = 4.6 Hz, 8H, b+c), 8.28 (d, J = 1.2 Hz, 8H, o), 8.01 (d, J = 1.2 Hz, 4H, p), 7.89 (s, 4H, e), 2.56 (t, J = 6.8 Hz, 4H, CPDIPS-CH_2), 2.24–2.17 (s+m, 16H, f + CPDIPS-CH_2), 1.20–1.16 (m, 4H, CPDIPS-CH_2) 1.55–0.81 (m, 340H, THS + CPDIPS-CH + CPDIPS-CH_3) ppm. **MALDI-ToF** *m/z* 3977.696 (calculated for $[C_{252}H_{396}N_{10}Si_{10}Zn_2]^+$ = $[$**P2**$]^+$: 3977.569). **UV-vis** ($CDCl_3$) *l* [nm] (ε [μM^{-1} cm^{-1}]): 635 (0.134), 581 (0.031), 537 (0.010), 447 (0.908).

3. cw-EPR Spectroscopy

3.1 Acquisition parameters

The continuous wave (cw) X-band EPR spectra were acquired on a Bruker EMXmicro spectrometer equipped with a high sensitivity Bruker probe head and an Oxford Instruments helium-flow cryostat ESR900. EPR spectra were recorded at a microwave frequency of ca. 9.40 GHz with a modulation amplitude and frequency of 100 mT and 100 kHz, respectively. The microwave power was scanned to rule out spectral distortions due to saturation effects. The optimal microwave power used in the room temperature cw-EPR spectra was 2.0 mW corresponding to an attenuation of 20 dB on our spectrometer. The temperature during variable temperature cw-EPR experiments was controlled with an Oxford Instruments digital temperature controller ITC503s using liquid nitrogen as cryogen. A waiting period of 1–1.5 hours between temperature changes or sample insertion and acquisition of the variable temperature cw-EPR spectra was used to ensure that thermal equilibrium was reached before the measurements. The reported spectra were obtained by combining several individually recorded scans to confirm that the signal shape remained constant over time.

3.2 Lineshape analysis and Norris equation

The initial analysis of the room temperature cw-EPR spectra was performed by fitting the first derivative of the following Gaussian normal distribution to the normalized data:

$$G(B) = \sqrt{\frac{2}{\pi}}\frac{1}{\Delta B_N}\mathrm{e}^{-2\left(\frac{B-B_{\mathrm{cf}}}{\Delta B_N}\right)} \quad \text{(S1)}$$

where B, B_{cf}, and ΔB_N are the magnetic field, the center field, and the Gaussian spectral envelope width, respectively. The fitting was performed using the inbuilt MATLAB function *lsqnonlin*. The resulting values of ΔB_N for **P1**$^{\bullet+}$ and **P2**$^{\bullet+}$ in CD_2Cl_2:toluene-*d*$_8$:THF-*d*$_8$ 1:1:1 and 2-methyltetrahydrofuran are summarized in Table S1 and plotted against $1/\sqrt{N}$ in Figure S3 to compare the experimental trend with the ideal Norris trend describe by equation 2 in the main manuscript.

Table S1. Summary of the Gaussian spectral envelope widths ΔB_N of the room temperature cw-EPR spectra of **P1**$^{\bullet+}$ and **P2**$^{\bullet+}$ in CD_2Cl_2:toluene-*d*$_8$:THF-*d*$_8$ 1:1:1 and 2-methyltetrahydrofuran.

	**P1**$^{\bullet+}$		**P2**$^{\bullet+}$	
solvent	CD_2Cl_2:toluene-*d*$_8$:THF-*d*$_8$ 1:1:1	MTHF	CD_2Cl_2:toluene-*d*$_8$:THF-*d*$_8$ 1:1:1	MTHF
ΔB_N / mT	0.66	0.69	0.48	0.50

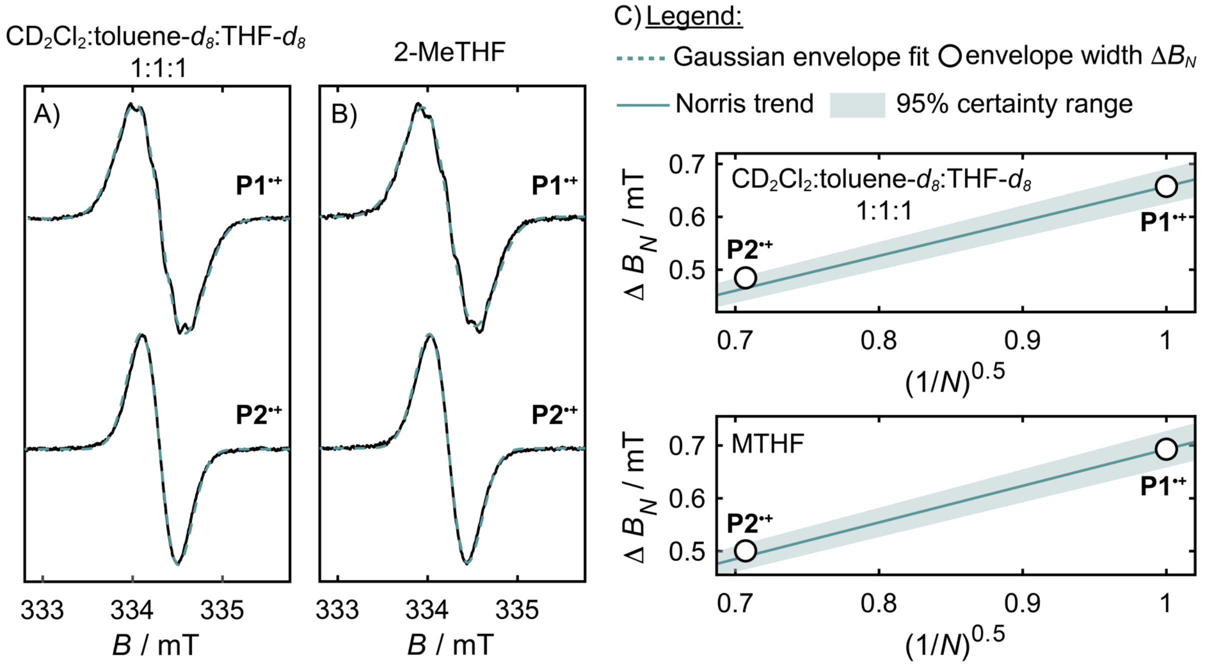

Figure S3. Spectral envelope width analysis of the room temperature (298 K) cw-EPR spectra (black lines) of **P1**$^{\bullet+}$ and **P2**$^{\bullet+}$ at X-band frequencies in A) CD_2Cl_2:toluene-*d*$_8$:THF-*d*$_8$ 1:1:1 and B) 2-methyltetrahydrofuran using a derivate Gaussian function to model the spectral envelope (dashed green lines). C) The agreement of the experimental spectral envelope trend with the Norris relationship is investigated by plotting the best-fit Gaussian envelope width ΔB_N in CD_2Cl_2:toluene-*d*$_8$:THF-*d*$_8$ 1:1:1 and 2-methyltetrahydrofuran against $(1/N)^{0.5}$. The theoretical predicted Norris trend described by equation 2 in the main manuscript is indicated by the green trend line and only dependent on the spectral envelope width ΔB_1 of **P1**$^{\bullet+}$. The light green shaded area represents the 95% confidence interval around the Norris trend based on a ±5% uncertainty in the monomer spectrum linewidth ΔB_1.

The cw-EPR spectra of **P1**$^{\bullet+}$ and **P2**$^{\bullet+}$ are well described by derivative Gaussian envelope functions which indicates that the dominant contribution to the spectral shapes are unresolved hyperfine interactions. The spectral envelope widths ΔB_N of both systems are slightly higher in MTHF compared to CD_2Cl_2:toluene-*d*$_8$:THF-*d*$_8$ 1:1:1 which is likely the result of small changes in the relaxation behavior and therefore the intrinsic Lorentzian linewidth Γ_N between the two solvent systems. The trend of the best-fit Gaussian spectra envelopes of **P1**$^{\bullet+}$ and **P2**$^{\bullet+}$ in both solvent systems follows closely the ideal Norris trend which indicates a uniform distribution of the radical spin density between both sides of the biphenyl-linked porphyrin dimer at 298 K in solution (Figure S3C). The Norris trendline in Figure S3C is determined by the spectral envelope width ΔB_1 of **P1**$^{\bullet+}$.

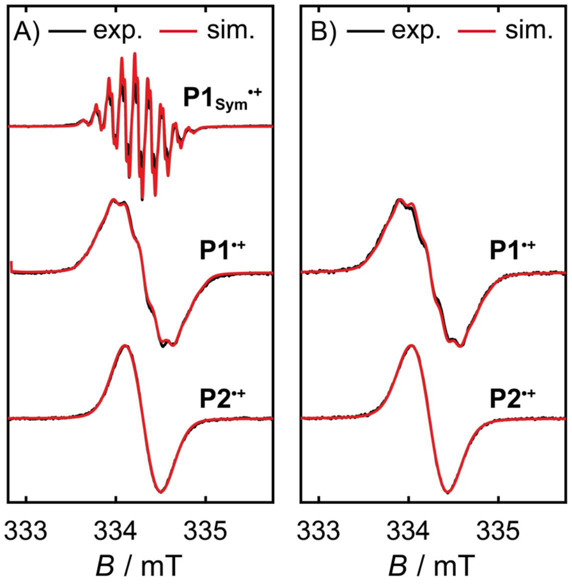

C)

CD_2Cl_2:toluene-d_8:THF-d_8 1:1:1	$^{14N}A_{iso}$ / MHz	^{14N}n	*lwpp*Gaussian / mT	*lwpp*Lorentzian / mT
**P1**$_{\mathbf{Sym}}^{\bullet+}$	3.99	4	0.00	0.03
**P1**$^{\bullet+}$	3.99	4	0.14	0.05
**P2**$^{\bullet+}$	2.00	8	0.14	0.05

MTHF	$^{14N}A_{iso}$ / MHz	^{14N}n	*lwpp*Gaussian / mT	*lwpp*Lorentzian / mT
**P1**$_{\mathbf{Sym}}^{\bullet+}$				
**P1**$^{\bullet+}$	3.99	4	0.13	0.08
**P2**$^{\bullet+}$	2.00	8	0.13	0.08

Figure S4. Numerical simulations (red) of the cw-EPR spectra of **P1**$_{\mathbf{Sym}}^{\bullet+}$, **P1**$^{\bullet+}$, and **P2**$^{\bullet+}$ (black) measured at 298 K at X-band frequencies in A) CD_2Cl_2:toluene-d_8:THF-d_8 1:1:1 and B) 2-methyltetrahydrofuran. C) Summary of the relevant simulation parameters. The best fit to the cw-EPR spectra of **P1**$_{\mathbf{Sym}}^{\bullet+}$ and **P1**$^{\bullet+}$ was found by least-squares fitting of the isotropic nitrogen hyperfine interaction $^{14N}A_{iso}$, the Gaussian and Lorentzian contributions to the Voigtian peak-to-peak linewidth, and in the case of **P1**$_{\mathbf{Sym}}^{\bullet+}$ the isotropic hydrogen hyperfine interaction $^{1H}A_{iso}$. Dimer **P2**$^{\bullet+}$ was simulated in agreement with the Norris relationship assuming a complete and uniform distribution of spin density over both sides with $^{14N}A_{iso}$(**P2**$^{\bullet+}$) = $^{14N}A_{iso}$(**P1**$^{\bullet+}$)/2.

3.3 Variable-temperature cw-EPR spectroscopy

The temperature dependence of the reversible intramolecular electron transfer in **P2**$^{•+}$ was investigated by variable-temperature (VT) cw-EPR spectroscopy of **P1**$^{•+}$ and **P2**$^{•+}$ between 298 K and 100 K in CD_2Cl_2:toluene-*d*$_8$:THF-*d*$_8$ 1:1:1 and 2-methyltetrahydrofuran (Figures S5, S6). The VT cw-EPR spectra of the butadiyne-linked porphyrin dimer cation ***b*-P2**$^{•+}$ were measured as a reference to differentiate between dynamic lineshape effects that result from the intramolecular electron transfer in **P2**$^{•+}$ and anisotropic broadenings due to slower tumbling rates at low temperatures (Figures S5D, S6D).

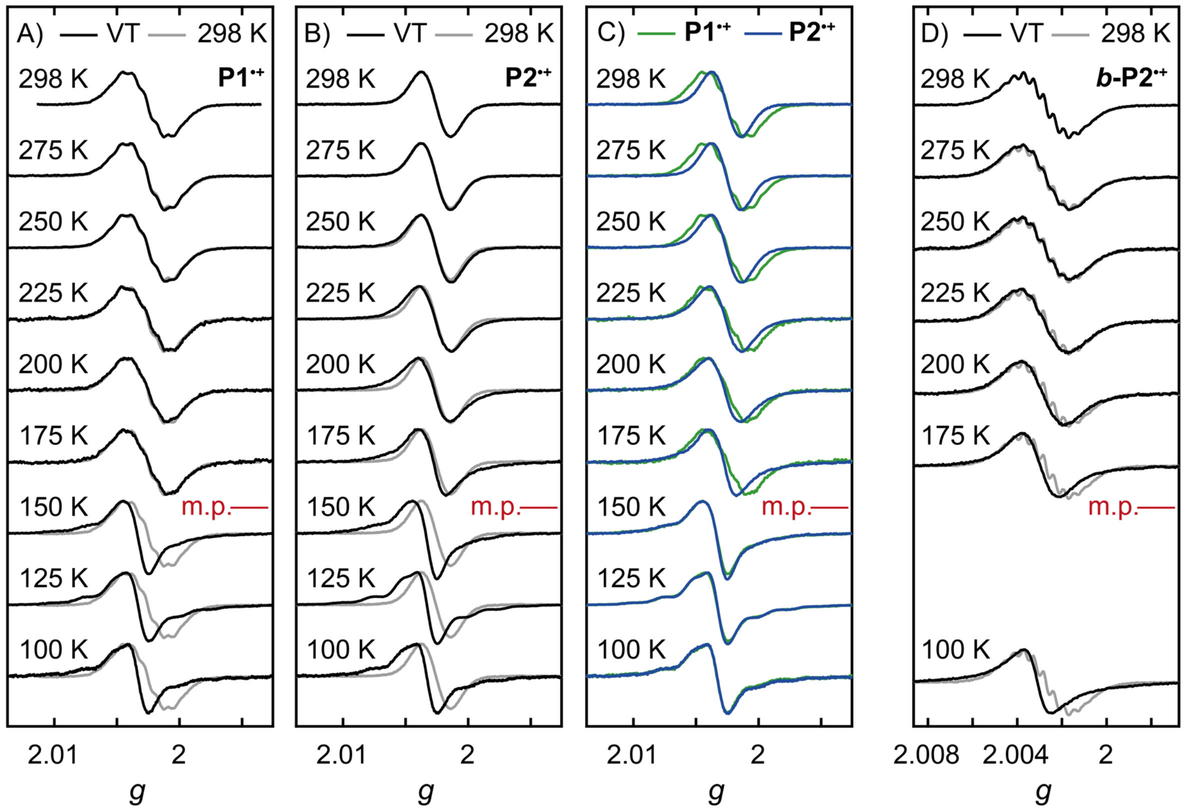

Figure S5. Variable temperature cw-EPR spectra of A) **P1**$^{•+}$, B) **P2**$^{•+}$, and D) ***b*-P2**$^{•+}$ in CD_2Cl_2:toluene-*d*$_8$:THF-*d*$_8$ 1:1:1 between 298 K and 100 K at X-band frequencies. At each temperature, the variable temperature spectrum (black) is compared to the corresponding spectrum at 298 K (gray). C) Comparison of the variable temperature cw-EPR spectra of **P1**$^{•+}$ (green) and **P2**$^{•+}$ (blue). All spectra at a temperature below the melting point (m.p.) of CD_2Cl_2:toluene-*d*$_8$:THF-*d*$_8$ 1:1:1 (ca. 160 K) were recorded in a frozen glass.

The variable temperature cw-EPR spectra of **P1**$^{•+}$, **P2**$^{•+}$, and ***b*-P2**$^{•+}$ were investigated in 2-methyltetrahydrofuran which has a larger accessible temperature window in the liquid state than CD_2Cl_2:toluene-*d*$_8$:THF-*d*$_8$ 1:1:1 (Figure S6). Between 298 K and 225 K the cw-EPR spectra of **P1**$^{•+}$ are unchanged which is consistent with temperature independent hyperfine interactions and sufficient tumbling to dynamically average the hyperfine and ***g*** anisotropies. Upon further cooling, the solution state cw-EPR spectra of **P1**$^{•+}$ exhibit increasing anisotropic broadening and spectral shapes that gradually approximate the anisotropic cw-EPR spectrum of **P1**$^{•+}$ in a frozen glass at 100 K. Noticeably, the cw-EPR spectra of **P1**$^{•+}$ in CD_2Cl_2:toluene-*d*$_8$:THF-*d*$_8$ 1:1:1 at 200 K and 175 K remain isotropic which confirms that the broadening in 2-methyltetrahydrofuran is influenced by the solvent properties. The spectra of **P2**$^{•+}$ continuously broaden with decreasing temperature. Comparison with the variable temperature spectra of ***b*-P2**$^{•+}$ shows that the linewidth changes in **P2**$^{•+}$ between 298 K and 225 K stem from the reversible intramolecular electrons transfer process whereas at lower temperatures anisotropic broadening substantially contributes to the spectral shape. The comparison of the variable temperature spectra of **P1**$^{•+}$ and **P2**$^{•+}$ shows that the electron transfer in **P2**$^{•+}$ remains faster than the EPR time scale

between 298 K and 200 K in 2-methyltetrahydrofuran. However, the solution state spectra of **P1**$^{\bullet+}$ and **P2**$^{\bullet+}$ below 200 K are identical which is consistent with a localization of the radical spin density in **P2**$^{\bullet+}$ on the EPR time scale. This is probably the combined result of the decreasing thermal energy and increasing viscosity of the solvent system which increases the outer reorganization energy associated with the electron transfer. The spectra of **P1**$^{\bullet+}$ and **P2**$^{\bullet+}$ in a frozen glass of 2-methyltetrahydrofuran at 100 K demonstrate that the radical in **P2**$^{\bullet+}$ remains localized on the EPR time scale which is consistent with the simulation of these cw-EPR spectra (Figure S8B).

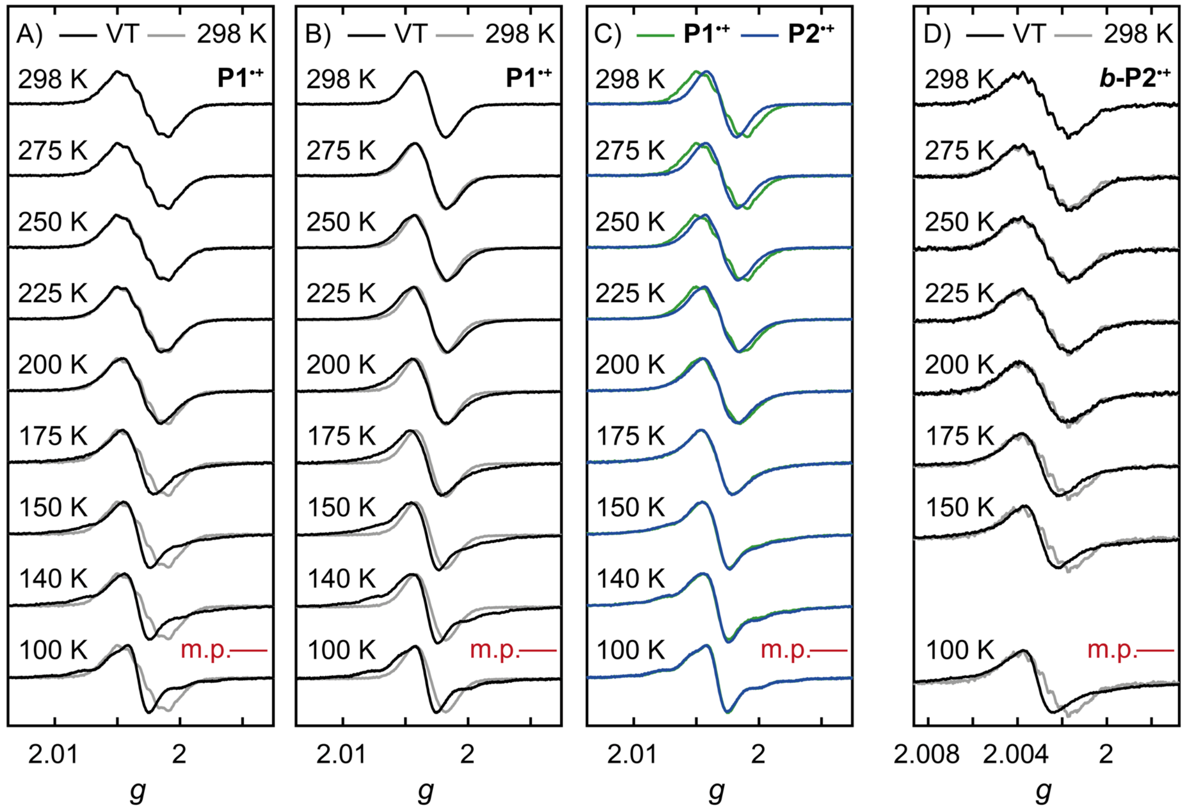

Figure S6. Variable temperature X-band cw-EPR spectra of A) **P1**$^{\bullet+}$, B) **P2**$^{\bullet+}$, and D) ***b*****-P2**$^{\bullet+}$ in 2-methyltetrahydrofuran between 298 K and 100 K. At each temperature, the variable temperature spectrum (black) is compared to the corresponding spectrum at 298 K (gray). C) Comparison of the variable temperature cw-EPR spectra of **P1**$^{\bullet+}$ (green) and **P2**$^{\bullet+}$ (blue). The spectra at 100 K were recorded in a frozen glass.

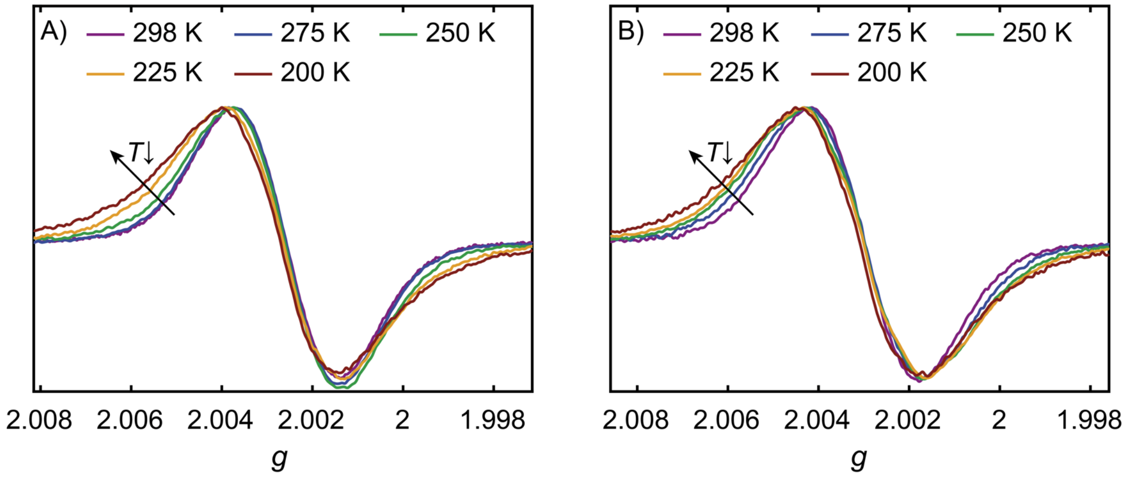

Figure S7. Superimposed variable temperature cw-EPR spectra of **P2**$^{\bullet+}$ in A) CD_2Cl_2:toluene-d_8:THF-d_8 1:1:1 and B) 2-methyltetrahydrofuran to highlight the increasing exchange broadening.

The frozen solution X-band cw-EPR spectra of **P1**$^{\bullet+}$ and **P2**$^{\bullet+}$ in CD_2Cl_2:toluene-*d8*:THF-*d8* 1:1:1 and 2-methyltetrahydrofuran at 100 K were simulated with the EasySpin function *pepper* using an anisotropic ***g***-tensor, the anisotropic ^{14}N hyperfine interactions to two representative nuclei calculated via the distributed point dipole model (*vide infra*), the quadrupole coupling of the ^{14}N nuclei calculated by DFT at the B3LYP/EPR-II level of theory (Q_1 = 2.36 MHz, η_1 = 0.60, Q_2 = 2.39 MHz, η_2 = 0.56), and an anisotropic linewidth. The good agreement between the experimental cw-EPR spectra and their simulations using ^{14}N hyperfine interactions calculated via the distributed point-dipole approach strengthens the validity of these calculations. In addition, all spectra could be simulated with the same set of hyperfine couplings which is evidence for the localization of the radical spin density on one porphyrin unit in **P2**$^{\bullet+}$ and the independence of the hyperfine interactions from the solvent system.

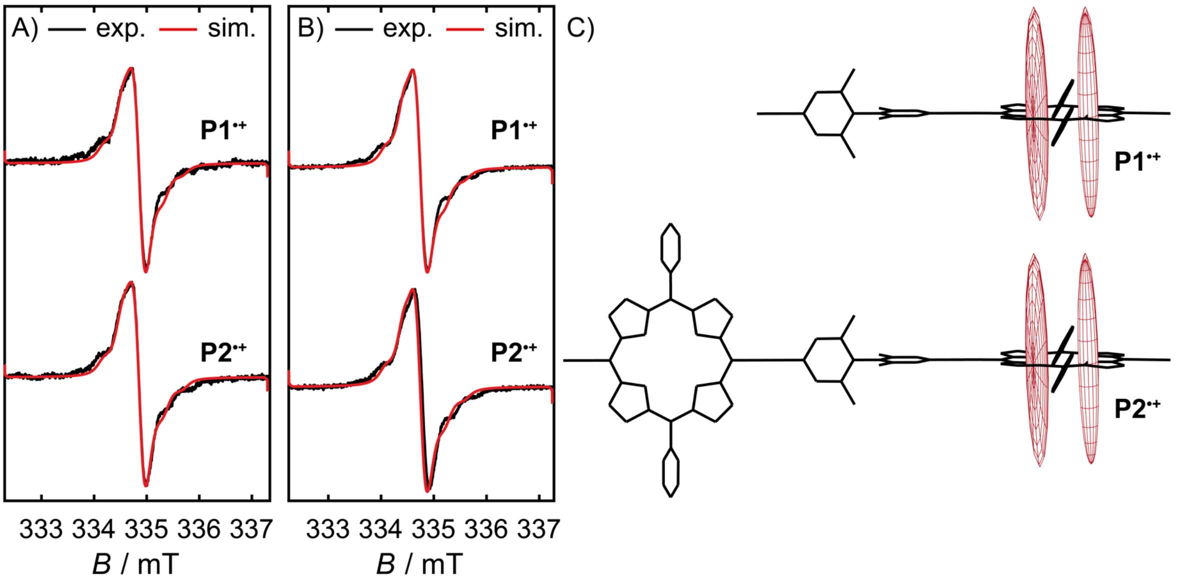

Figure S8. Experimental (black) and simulated (red) cw-EPR spectra of **P1**$^{\bullet+}$ and **P2**$^{\bullet+}$ at 100 K and X-band frequencies in a frozen glass of A) CD_2Cl_2:toluene-*d8*:THF-*d8* 1:1:1 and B) 2-methyltetrahydrofuran. C) Pictorial representation of the ^{14}N hyperfine coupling tensors $^{14N}\boldsymbol{A}$ of **P1**$^{\bullet+}$ and **P2**$^{\bullet+}$ used to simulate the cw-EPR spectra. The tensors are shown as ellipsoids centered at the corresponding ^{14}N nuclei with elliptic radii proportional to the eigenvalues of $^{14N}\boldsymbol{A}$ and an orientation in the molecular frame given by their Euler angles.

4. Distributed Point-Dipole Model for Calculating Hyperfine Interactions

The simulation of the Q-band ^{1}H Mims ENDOR spectra of **P1$^{•+}$** and **P2$^{•+}$** presented in Section 5 using DFT calculated hyperfine tensors is not feasible due to an over-delocalization of the radical spin density from the porphyrin part to the biphenyl part of **P1$^{•+}$** for a wide range of DFT functionals with different range-separation parameters and amounts of exact Hartree–Fock exchange. All hyperfine tensor calculations were performed on the optimized geometry of **P1$^{•+}$** with the EPR-II basis set. The inaccurate prediction of the radical spin density distribution results in unreliable hyperfine tensor calculations as evident from the stark exaggeration of the largest anisotropic hyperfine contributions A_{max} in the simulated ^{1}H Mims ENDOR spectra of **P1$^{•+}$**, which arises from the aromatic biphenyl protons (Figure S9).

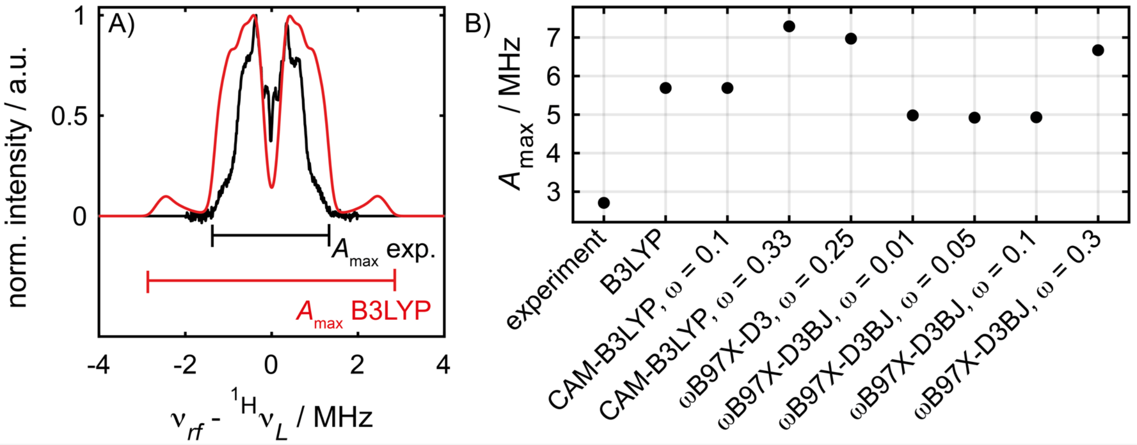

Figure S9. A) Experimental (black) and simulated (red) ^{1}H Mims ENDOR spectra of **P1$^{•+}$** at Q-band frequencies in CD_2Cl_2:toluene-*d*$_8$:THF-*d*$_8$ 1:1:1 at 80 K. The largest anisotropic hyperfine contributions A_{max} to the ENDOR spectra are determined from their width. B) Comparison of the experimental and simulated largest hyperfine contributions A_{max} to the **P1$^{•+}$** ENDOR spectra using hyperfine tensors calculated with a range a DFT functionals.

To overcome this limitation, we employed a distributed point-dipole model to calculate the ^{1}H and ^{14}N hyperfine interactions in **P1$^{•+}$** for a range of spin density distributions between its porphyrin and biphenyl parts. The hyperfine interactions that resulted in the best simulation of the experimental ^{1}H ENDOR spectrum of **P1$^{•+}$** were then used to simulate the Mims ENDOR spectra of **P1$^{•+}$** and **P2$^{•+}$** in CD_2Cl_2:toluene-*d*$_8$:THF-*d*$_8$ 1:1:1 and 2-methyltetrahydrofuran, and the exchange broadened variable temperature cw-EPR spectra of **P2$^{•+}$**. The procedure used to calculate the hyperfine tensors is detailed below.

Step 1: assignment of the spin density distribution

To systematically adjust the spin density distribution in **P1$^{•+}$**, the monomer is formally divided in a porphyrin and biphenyl subunit (Figure S10A). For each subunit, the relative spin density distributions χ_i were calculated using DFT predicted Mulliken spin populations following equation S2:

$$\chi_i = \frac{\rho_i}{\rho_{\mathrm{subunit}}} \quad \text{(S2)}$$

We define χ_i as the amount of spin density assigned to an individual atom i relative to the total amount of spin density assigned collectively to all atoms in the subunit ρ_{subunit}. This definition allows for a redistribution of spin density between the porphyrin and biphenyl parts of **P1$^{•+}$** without modifying how the spin density is distributed to each individual atom. For a robust assignment of the relative spin density distributions, χ_i was separately calculated using Mulliken spin populations obtained from DFT calculations with B3LYP, CAM-B3LYP ω = 0.1, ωB97X-B3LYP ω = 0.01, ωB97X-B3LYP ω = 0.05, and ωB97X-B3LYP ω = 0.1 as functionals and the EPR-II basis set. The functionals were chosen because the ^{1}H Mims ENDOR simulation of **P1$^{•+}$** using hyperfine interactions calculated with these functionals deviated least from the experimental spectrum (Figure S9B). For each atom the relative spin density distribution calculated for the five different DFT functionals was averaged to give the final relative spin

density distribution $\chi_{i,\mathrm{av}}$ employed in the point-dipole model. Encouragingly, the relative spin densities of all atoms show only subtle deviations when calculated with Mulliken population obtained with different DFT functionals (Figure S10B).

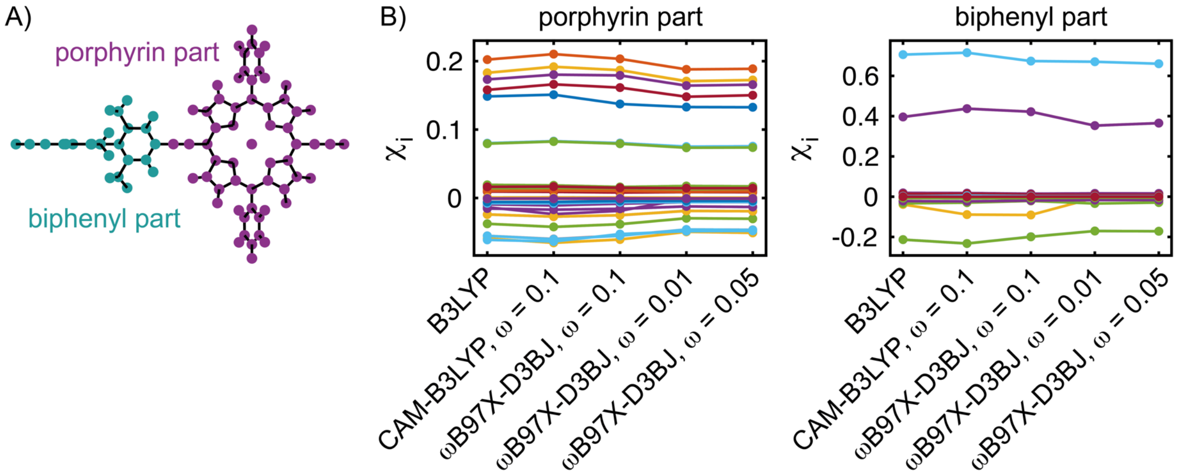

Figure S10. A) Schematic visualization of the separation of **P1**$^{\bullet+}$ in a porphyrin (purple) and biphenyl (green) fragment. B) Trend in the relative spin density distribution χ_i for each atom in the porphyrin and biphenyl part of **P1**$^{\bullet+}$ calculated using Mulliken spin populations obtain with different DFT functionals. The connections between points of the same color are a guide to the eye for the functional dependence of χ_i for a given atom.

We define the spin density ratio Δ as quantitative measure of the radical delocalization in **P1**$^{\bullet+}$ using equation S3:

$$\Delta = \frac{\rho(\text{porphyrin})}{\rho(\text{porphyrin})+\rho(\text{biphenyl})} \tag{S3}$$

where ρ(porphyrin) and ρ(biphenyl) are the total spin populations on the porphyrin and biphenyl subunits, respectively. Modification of the spin density ratio Δ followed by the distribution of the spin density assigned to the porphyrin and biphenyl subunits of **P1**$^{\bullet+}$ to individual atoms using $\chi_{i,\mathrm{av}}$ allows for a systematic screening of radical delocalization with a single fitting parameter.

To account for the π-radical character of **P1**$^{\bullet+}$, spin densities assigned to sp^2-hybridized atoms were modelled to be localized in in *p*-type orbitals and split above and below the respective aromatic plane of the molecule. This is exemplarily shown for a spin density ratio Δ = 86.3% predicted at the B3LYP/EPR-II level of theory in Figure S11. The distance between the spin density and aromatic plane was set to ±52 pm in agreement with previous point-dipole simulations of porphyrin oligomers.

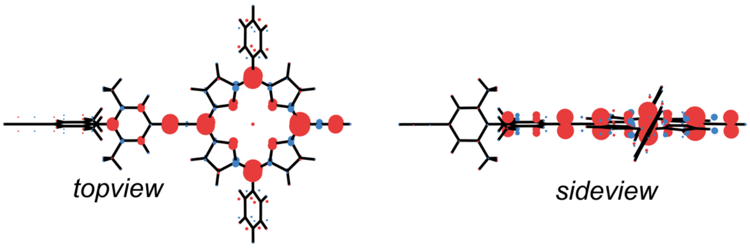

Figure S11. Visualization of the π-orbital type spin centers in **P1**$^{\bullet+}$ for a spin density ratio Δ = 86.3% predicted by DFT calculations at the B3LYP/EPR-II level of theory. The spin density at each point in space is represented by a sphere centered on the position of the spin center with a radius proportional to the magnitude of its assigned spin density. Red spheres represent excess spin-up and light-blue spheres indicate excess spin-down density.

Step 2: calculation of the anisotropic dipolar hyperfine coupling contributions

Using the spin density distributions established in step 1, the dipolar hyperfine tensors $\boldsymbol{T}_i$ of each ^{1}H and ^{14}N nucleus in **P1$^{•+}$** were calculated following equation S4:

$$\boldsymbol{T}_i = \frac{\mu_0}{4\pi\hbar} g_\mathrm{e}\beta_\mathrm{e} g_\mathrm{n}\beta_\mathrm{n} \sum_{\mathrm{k}\neq i} \rho_\mathrm{k} \frac{(3\boldsymbol{n}_\mathrm{k}\boldsymbol{n}_\mathrm{k}^\mathrm{T} - \boldsymbol{1})}{R_\mathrm{k}^3} \quad \text{(S4)}$$

with a sum over all spin centers k not located on the nucleus i with a spin density ρ_k and a distance R_k from nucleus i. The unit vector $\boldsymbol{n}_\mathrm{k}$ defines the orientation of R_k in the molecular frame. This point-dipole approach approximates the dipolar hyperfine contributions as through space dipole-dipole interactions between nucleus i and discrete spin centers localized at each atom.[4,5]

Step 3: calculation of the isotropic hyperfine coupling contribution

The isotropic contributions to the hyperfine tensors $A_{\mathrm{iso},i}$ for each ^{1}H nucleus i are calculated based on the McConnell relationship following equation S5:

$$A_{\mathrm{iso},i} = c_i \cdot \rho_i \quad \text{(S5)}$$

where ρ_i is the spin density assigned to nuclei i in step 1 and c is the McConnell proportionality constant.[6] While McConnell's seminal work related the isotopic hyperfine interactions of aromatic protons to the amount of spin density on the carbon atom they are attached to, we approximated this relationship by directly assuming a proportional relationship between $^{1\mathrm{H}}A_{\mathrm{iso},i}$ and $^{1\mathrm{H}}\rho_i$.[6] The proportionality constant c_i was calculated for each ^{1}H nucleus in **P1$^{•+}$** using its DFT precited Mulliken spin populations and isotropic hyperfine interactions with B3LYP, CAM-B3LYP ω = 0.1, ωB97X-B3LYP ω = 0.01, ωB97X-B3LYP ω = 0.05, and ωB97X-B3LYP ω = 0.1 as functionals and the EPR-II basis set. The final proportionality constant $c_{i,\mathrm{av}}$ employed in the point-dipole model was obtained by averaging the McConnell constants for the different functionals. The isotropic hyperfine coupling to the ^{14}N nuclei $^{14\mathrm{N}}A_\mathrm{iso}$ = 3.99 MHz was obtained from least square fitting of the room temperature cw-EPR spectra of **P1$^{•+}$** (Figure S4).

Step 4: calculation of the full hyperfine tensors

By combining the dipolar and isotropic hyperfine contributions $\boldsymbol{T}_i$ and $A_{\mathrm{iso},i}$ derived in steps 2 and 3, we can now calculate the full hyperfine interaction tensors $\boldsymbol{A}_i$ following equation S6:

$$\boldsymbol{A}_i = A_{\mathrm{iso},i} \begin{pmatrix} 1 & 0 & 0 \\ 0 & 1 & 0 \\ 0 & 0 & 1 \end{pmatrix} + \boldsymbol{T}_i \quad \text{(S6)}$$

Step 5: fitting of the spin density ratio in **P1$^{•+}$** to the experimental ^{1}H Mims ENDOR spectrum

The optimal spin density ratio to describe **P1$^{•+}$** was determined by comparison of the experimental Q-band ^{1}H Mims ENDOR spectrum of **P1$^{•+}$** with a range of simulated spectra using anisotropic ^{1}H hyperfine tensors $^{1\mathrm{H}}\boldsymbol{A}$ calculated via the distributed point dipole model for a series of spin density ratios Δ (Figure S12). The best agreement between the experimental and simulated spectra was found for a spin density ratio Δ = 96.1% as judged by the root-mean-square deviation (*rmsd*) between the two spectra (Figure S12B).

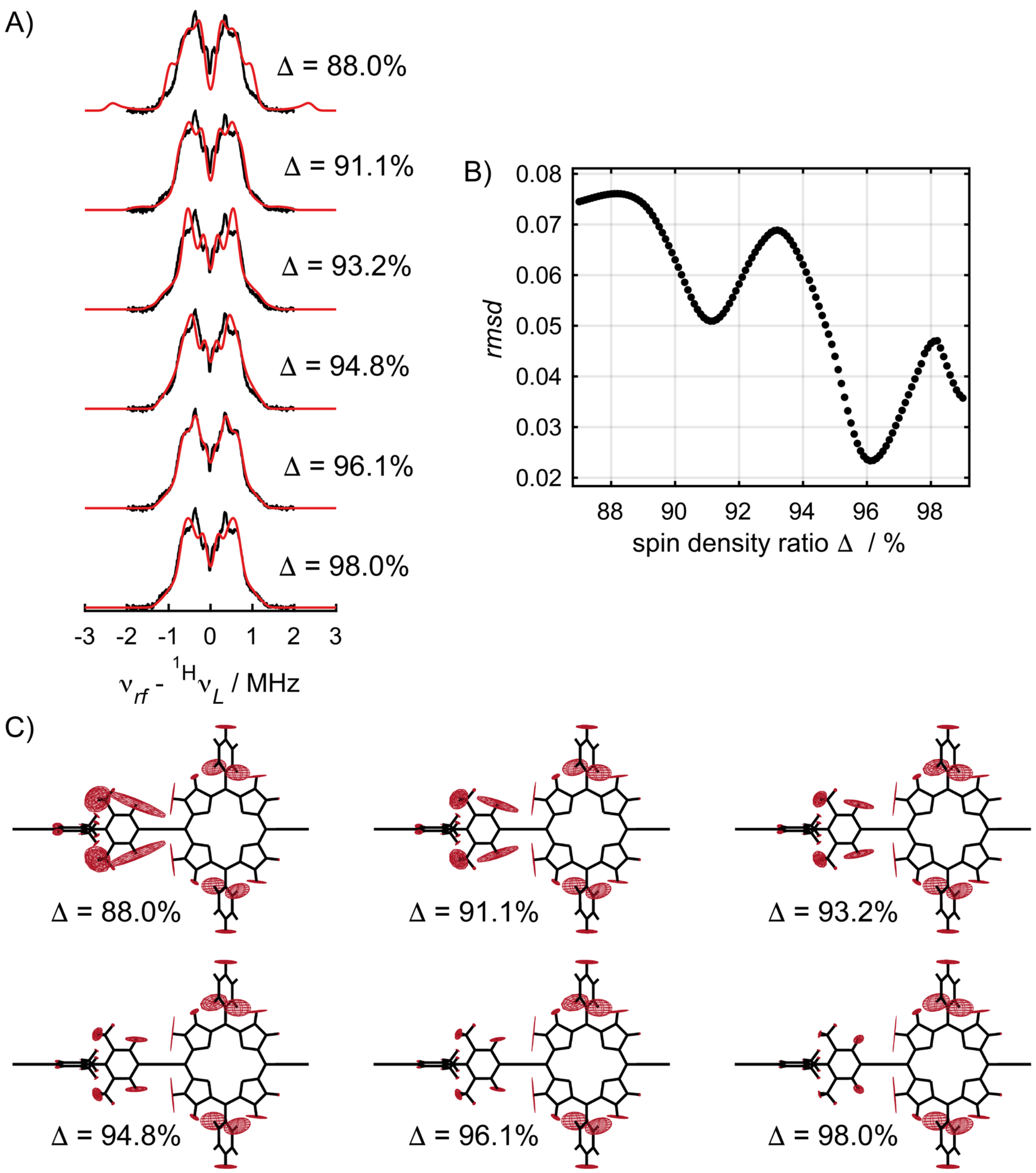

Figure S12. A) Experimental (black) and simulated (red) ^{1}H Mims ENDOR spectra of **P1$^{\bullet+}$** at Q-band frequencies in CD_2Cl_2:toluene-d_8:THF-d_8 1:1:1 at 80 K. The simulated spectra were obtained with anisotropic ^{1}H hyperfine tensors $^{1H}\boldsymbol{A}$ calculated via the distributed point-dipole model for the respective spin density ratios Δ. B) Root-mean-square deviation (*rmsd*) between the experimental and simulated ^{1}H Mims ENDOR spectra of **P1$^{\bullet+}$** as a function of Δ. C) Pictorial representation of $^{1H}\boldsymbol{A}$ for the respective spin density ratios Δ. The tensors are shown as ellipsoids centered at the corresponding ^{1}H nuclei with elliptic radii proportional to the eigenvalues of $^{1H}\boldsymbol{A}$ and an orientation in the molecular frame given by their Euler angles.

5. ^{1}H Mims ENDOR Spectroscopy

The ^{1}H Mims ENDOR spectra of **P1**$_{\mathbf{Sym}}^{\bullet+}$, **P1**$^{\bullet+}$, and **P2**$^{\bullet+}$ were recorded at Q-band frequencies at 80 K in CD_2Cl_2:toluene-d_8:THF-d_8 1:1:1 or 2-methyltetrahydrofuran with the pulse sequence $\pi/2-\tau-\pi/2-T-\pi/2-\tau-echo$ with a microwave pulse length $t_{\pi/2}$ = 16 ns and a radiofrequency pulse length t_{rf} = 18 µs at the field position with the largest signal intensity (corresponding to $g_{x,y}$) . All ^{1}H ENDOR spectra were acquired with three different τ-values (τ = 240 ns, 300 ns, and 360 ns) to rule out distortions arising from the blind-spot characteristics of the Mims ENDOR sequence. Before each ENDOR measurement, the power of the radiofrequency pulses was adjusted to obtain a π-pulse using a Rabi nutation experiment at the ^{1}H Larmor frequency $^{1\mathrm{H}}\nu_L$ (ca. 51.5 MHz). The ^{1}H Mims ENDOR spectrum of **P1**$_{\mathbf{Sym}}^{\bullet+}$ was simulated using the hyperfine tensors $^{1\mathrm{H}}\boldsymbol{A}$ obtained from DFT calculations with B3LYP as functional and the EPR-II basis set (see Table S2 for principal hyperfine interactions). The hyperfine tensors for the simulation of **P1**$^{\bullet+}$ and **P2**$^{\bullet+}$ were obtained from the distributed point-dipole model described in Section 4 with a spin density ratio Δ = 96.1% and reported in Table S2. The ENDOR simulations were performed separately for each ^{1}H nucleus with the EasySpin function *saffron* using the relevant experimental τ-values (240 ns, 300 ns, and 360 ns), microwave pulse lengths $t_{\pi/2}$ (16 ns) and experimental field positions, as well as an inherent linewidth of 0.25 MHz. The principal values of the anisotropic ***g***-tensors employed in the ENDOR simulations of **P1**$_{\mathbf{Sym}}^{\bullet}$ and **P1**$^{\bullet+}$ are [2.0023 2.0027 2.0030] and [2.0025 2.0025 2.0019], respectively. Appropriate Euler angle rotations obtained from DFT and/or point-dipole calculations were employed in all simulations to allow for the accurate description of orientation selection effects arising from the relative orientation of the *g*- and hyperfine interaction frameworks.

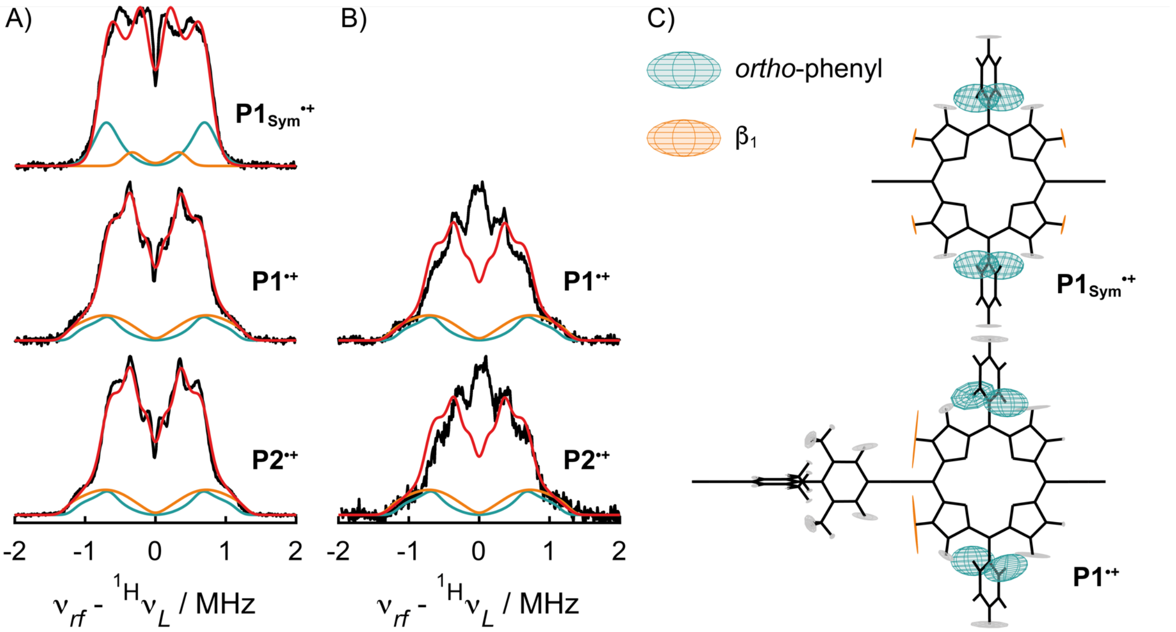

Figure S13. Experimental (black) and simulated (red) ^{1}H Mims ENDOR spectra of **P1**$_{\mathbf{Sym}}^{\bullet+}$, **P1**$^{\bullet+}$, and **P2**$^{\bullet+}$ recorded at Q-band frequencies in A) CD_2Cl_2:toluene-d_8:THF-d_8 1:1:1 and B) 2-methyltetrahydrofuran at 80 K. The spectrum of **P1**$_{\mathbf{Sym}}^{\bullet+}$ was simulated using anisotropic ^{1}H hyperfine tensors $^{1H}\boldsymbol{A}$ obtained from DFT calculations at the B3LYP/EPR-II level of theory. The spectra of **P1**$^{\bullet+}$ and **P2**$^{\bullet+}$ were simulated with identical simulation parameters using hyperfine tensors calculated *via* the distributed point-dipole model for **P1**$^{\bullet+}$ with a spin density ration Δ = 96.1%. C) Schematic representation of the ^{1}H hyperfine tensors of **P1**$_{\mathbf{Sym}}^{\bullet+}$ and **P1**$^{\bullet+}$. The tensors of the *ortho*- and β_1-protons that give rise to the highlighted transitions in A) are shown in green and orange, respectively; additional tensors are shown

Table S2. Summary of the DFT (B3LYP/EPR-II) and point-dipole predicted isotropic contributions, A_{iso}, and principal values of the dipolar contributions, A_{dip}, to the largest ^{1}H hyperfine interactions in **P1$_{Sym}$$^{•+}$** and **P1$^{•+}$**. The point-dipole calculations were performed for a spin density ratio Δ = 96.1%.

**P1$_{Sym}$$^{•+}$**	**DFT (B3LYP/EPR-II)**		**point-dipole model**	
	A_{iso} / MHz	A_{dip} / MHz	A_{iso} / MHz	A_{dip} / MHz
ortho-phenyl	–1.19	[1.36 –0.22 –1.14]	–	–
para-phenyl	–0.94	[0.81 –0.17 –0.64]	–	–
β_1	0.07	[0.00 –0.79 0.78]	–	–
β_2	0.25	[–0.07 –0.71 0.77]	–	–
**P1$^{•+}$**	**DFT (B3LYP/EPR-II)**		**point-dipole model**	
	A_{iso} / MHz	A_{dip} / MHz	A_{iso} / MHz	A_{dip} / MHz
ortho-phenyl	–1.25	[1.07 –0.13 –0.94]	–1.32	[0.95 –0.01 0.95]
para-phenyl	–1.17	[0.90 –0.14 –0.76]	–1.16	[1.33 –0.43 –0.90]
β_1	0.93	[–0.89 –0.50 1.40]	0.95	[–0.90 –0.74 1.64]
β_2	–0.65	[0.46 0.14 –0.60]	–0.66	[0.81 –0.06 –0.75]
ortho-biphenyl	–4.03	[2.32 –0.87 –1.45]	–1.11	[1.10 –0.10 –1.00]
CH_3-biphenyl	–2.30	[0.52 –0.16 –0.36]	–0.62	[0.24 –0.08 –0.16]

6. Simulation of the Electron Transfer Dynamics

6.1 General simulation procedure

The isotropic variable temperature cw-EPR spectra of **P1**$^{•+}$ and **P2**$^{•+}$ in CD_2Cl_2:toluene-d_8:THF-d_8 1:1:1 and 2-methyltetrahydrofuran between 298 K and 200 K were simulated with a chemical exchange model implemented by Stoll and Kozhanov in MATLAB based on the EasySpin software package.[7,8,9] Simulations were performed using the Liouville simulation method for a general multisite exchange with identical inherent linewidths Γ for the exchanging sides. The magnetic interactions of the radical electron on the redox centers involved in the reversible intramolecular electron transfer in **P2**$^{•+}$ are analogous to the magnetic interactions in **P1**$^{•+}$. All simulations were performed with an isotropic *g*-value and isotropic hyperfine interactions obtained either from least-squares fitting of the cw-EPR spectrum or from the point-dipole simulations of the ^{1}H Mims ENDOR spectrum of **P1**$^{•+}$. The inherent linewidth Γ at each temperature was determined by simulating the cw-EPR spectra of **P1**$^{•+}$ using the chemical exchange model in the slow exchange limit (k_{ex} = 10^{-10} MHz) and was subsequently used to describe the two redox sites in the simulation of the variable temperature cw-EPR spectra of **P2**$^{•+}$ at this temperature in the same solvent system. The cw-EPR spectra of **P2**$^{•+}$ were simulated for a range of electron transfer rates k_{ex} and the best-fit simulation was identified from the smallest root-mean-square deviation (*rmsd*) between the experimental and simulated spectra. The error margins of k_{ex} were determined as the electron transfer rates with a 5% deviation of their *rmsd* from the minimum *rmsd*. The simulations of all variable temperature cw-EPR spectra of **P1**$^{•+}$ and **P2**$^{•+}$ were performed by calling the chemical exchange model by Stoll and Kozhanov from a custom written MATLAB routine that allowed the simultaneous simulation of multiple spectra using a *parfor*-loop included in the MATLAB parallel computing toolbox. All exchange simulations were performed on a high-performance computing cluster.

6.2 Fundamental aspects of exchange broadening in isotropic cw-EPR spectra

The influence of the reversible intramolecular electron transfer between the two degenerate redox sites of **P2**$^{•+}$ on the cw-EPR spectra of the system can be modelled as a two-state chemical exchange process in which the radical spin density is either distributed over one half or the other half of **P2**$^{•+}$ (Figure S14).

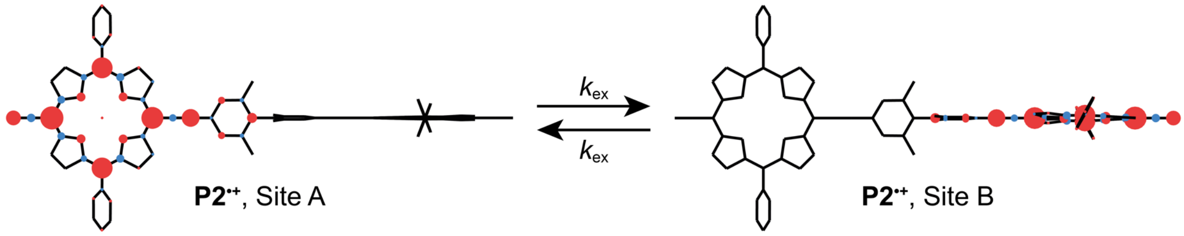

Figure S14. Schematic visualization of the redistribution of spin density during the reversible intramolecular electron transfer in **P2**$^{•+}$. The spin population on each nucleus is represented by a sphere centered on the atom with a radius proportional to the magnitude of its assigned spin population. Red spheres represent excess spin-up and light-blue spheres indicate excess spin-down populations.

The effects of a modulation of hyperfine interactions for dynamically equivalent nuclei on the cw-EPR spectrum of the system are exemplarily discussed below for a simple model system using numerical linewidth equations reported in an account by Fraenkel[10] and derived from relaxation-matrix theory[11] and the generalized Bloch equations[12,13,14]. In the simplified model system, we consider a free electron that alternatingly interacts with two ^{14}N nuclei with an isotropic hyperfine interaction of 30 MHz (Figure S15A). The simulations of the exchange broadened EPR spectra were performed with a magnetic field strength B = 320 mT and an intrinsic transverse relaxation time $T_{2,0} = 1 \cdot 10^{-7}$ s. Exchange induced frequency shifts of the EPR transitions are negligible in this simple example and were therefore omitted in the following discussions. We also assume an identical nuclear-spin quantum number m on both atoms before and after the electron transfer.

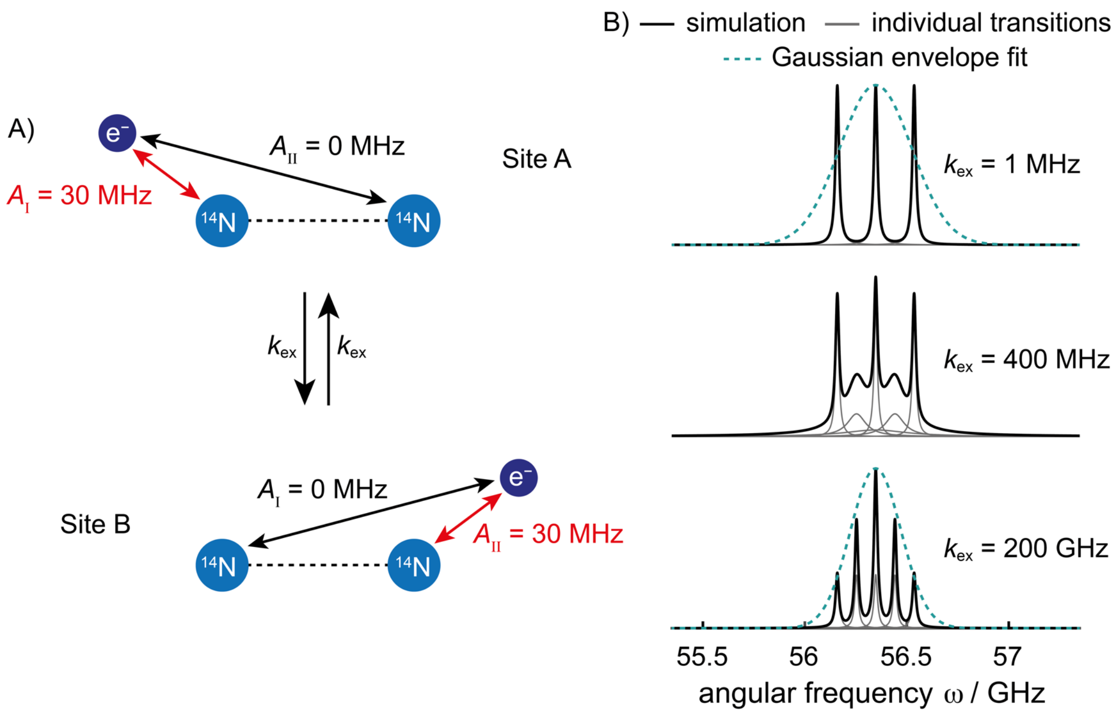

Figure S15. A) Pictorial representation of a simple model system of a reversible intramolecular electron transfer with a modulation of hyperfine interaction. The free electron e^- alternatingly interacts with two ^{14}N nuclei with an isotropic hyperfine interaction A = 30 MHz and a transfer rate k_{ex} between the nuclei. B) Numerical simulations of the absorptive EPR spectra of the model system in A) for transfer rates k_{ex} in the slow (top), intermediate (middle), and fast (bottom) exchange regime. The grey spectra indicate the individual transitions for each quantum number M, and their sum gives rise to the simulated spectra (black). The Gaussian spectral envelopes were obtained by least-squares fitting of a Gaussian normal distribution to the simulated spectra.

The angular frequencies $\omega(M)$ of the one-electron-two-nuclei model system are to first order given by equation S7:

$$\omega(M) = \omega_0 + |\gamma_e| M \frac{(A_I + A_{II})}{2} \quad \text{(S7)}$$

where γ_e is the gyromagnetic ration of the free electron, $\omega_0/2\pi$ is the electron-spin Larmor frequency, and A_I and A_{II} are the instantaneous hyperfine interactions on site A or B of the two-site exchange process. The quantum numbers M of the transitions are all possible combinations of the nuclear quantum numbers m of the two ^{14}N nuclei: $M = m_I + m_{II}$. The Lorentzian line shape $I_M(\omega)$ of the individual transitions M is given by equation S8.

$$I_M(\omega) = \frac{T_2(M)}{\pi}\{1 + T_2^2(M)[\omega - \omega(M)]^2\}^{-1} \quad \text{(S8)}$$

The transverse relaxation time $T_2(M)$ of each transition depends on the rate of the exchange process following equation S9.

$$T_2^{-1}(M) = T_2^{-1}(m_I, m_{II}) = {\gamma_e}^2 \tau \langle(\delta A)^2\rangle (m_I - m_{II})^2 + T_{2,0}^{-1} \quad \text{(S9)}$$

where $\langle(\delta A)^2\rangle = 0.25(A_I - A_{II})^2$ is the mean square deviation of the hyperfine splitting and $T_{2,0}$ is the intrinsic transverse relaxation time of the system and independent from the nuclear transition.

The lifetime τ depends on the exchange rate k_{ex} between sites A and B and is calculated following equation S10.

$$\tau = \frac{1}{2k_{ex}} \quad \text{(S10)}$$

As shown in the numerical simulations in Figure S15B, the appearance of the EPR spectra of the simple model system is determined by the rate of the electron transfer k_{ex} between the two nuclei. In the slow exchange limit ($\tau > (|\gamma_e||\delta A|)^{-1}$) the spectrum consists of a triplet with identical amplitudes for each transition as expected for a hyperfine coupling pattern for an *I* = 1 nucleus. For an intermediate electron transfer rate, the simulated spectrum is exchange broadened and exhibits an alternating line-width effect.[10] In the fast exchange limit ($\tau^2 < (\gamma_e{}^2\langle(\delta A)^2\rangle)^{-1}$), a five line spectrum with relative amplitudes 1:2:3:2:1 is observed and the Gaussian envelope width of the spectrum is reduced by a factor of about $2^{-0.5}$ compared to the envelope width in the slow exchange limit.

6.3 Simulation of P1•+ without phenomenological broadening

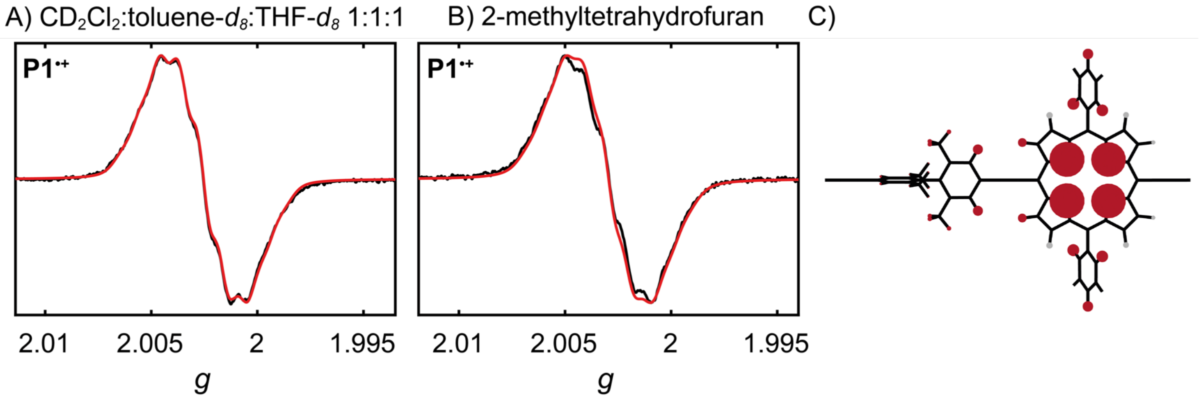

Figure S16. Experimental (black) and simulated (red) cw-EPR spectra of **P1•+** at 298 K and X-band frequencies in A) CD_2Cl_2:toluene-*d8*:THF-*d8* 1:1:1 and B) 2-methyltetrahydrofuran. Simulations were performed with the isotropic hyperfine interactions in Table S3 and a Lorentzian peak-to-peak linewidth of 0.05 mT in CD_2Cl_2:toluene-*d8*:THF-*d8* 1:1:1 and 0.07 mT in 2-methyltetrahydrofuran. C) Pictorial representation of the isotropic hyperfine interactions A_{iso} used to simulations of the cw-EPR spectra. The hyperfine interaction to each ^{1}H and ^{14}N nucleus is represented by a sphere centered on the atom with a radius proportional to the magnitude of A_{iso}. Hyperfine interactions not included in the simulations are shown in grey as reference.

The isotropic ^{1}H hyperfine couplings included in the simulations of **P1•+** and **P2•+** without phenomenological Gaussian broadening are listed in Table S3 and schematically visualized in Figure S16C and correspond to the interactions with the *ortho*- and *para*-protons of the aryl sidechains, the *ortho*-phenyl and CH_3 protons of the biphenyl linker, and the porphyrin β_1-protons. It should be noted that the three individual ^{1}H nuclei in the biphenyl CH_3 group are not equivalent in the point-dipole calculations. To account for the fast dynamic averaging of the CH_3 protons through rapid rotation of the CH_3 group in solution we have averaged their isotropic ^{1}H hyperfine interactions to obtain the value used for the cw-EPR simulations.

Table S3. Summary of the ^{1}H and ^{14}N isotropic hyperfine interactions A_{iso} and the number of equivalent nuclei *n* for each hyperfine interaction used to simulate the cw-EPR spectra of **P1•+** and the exchange broadened variable temperature cw-EPR spectra of **P2•+**.

Δ = 96.1%	^{14}N	*ortho*-aryl	*para*-aryl	β_1	*ortho*-biphenyl	CH_3-biphenyl
A_{iso} / MHz	3.99	1.23	1.16	1.11	0.95	0.44
n	4	4	2	2	2	6

6.4 Simulation of the variable temperature cw-EPR spectra of $P1^{\bullet+}$ and $P2^{\bullet+}$

The variable temperature cw-EPR spectra of **P1**$^{\bullet+}$ and **P2**$^{\bullet+}$ in CD_2Cl_2:toluene-d_8:THF-d_8 1:1:1 and 2-methyltetrahydrofuran between 298 K and 200 K were simulated with a two-site chemical exchange model following the approach detailed in Section 6.1 (Figures S17, S19). Due to the virtually identical cw-EPR spectra at 100 K and ^{1}H Mims ENDOR spectra of **P1**$^{\bullet+}$ in both solvent systems we use the same hyperfine interactions calculated with the point-dipole model for a spin density ratio Δ = 96.1% to simulate the variable temperature cw-EPR spectra in CD_2Cl_2:toluene-d_8:THF-d_8 1:1:1 and 2-methyltetrahydrofuran (Table S3). The best-fit peak-to-peak linewidths for simulating the cw-EPR spectra of **P1**$^{\bullet+}$ and electron transfer rates k_{ex} for the simulation of **P2**$^{\bullet+}$ in CD_2Cl_2:toluene-d_8:THF-d_8 1:1:1 are summarized in Tables S4 and S5, respectively. The analogous values used to simulate the variable temperature cw-EPR spectra of **P1**$^{\bullet+}$ and **P2**$^{\bullet+}$ in 2-methyltetrahydrofuran are given in Tables S6 and S7. The root-mean-square deviation between the experimental and simulated spectra of **P2**$^{\bullet+}$ for a range of electron transfer rates was used to determine the best simulation of the experimental spectra with the *rmsd* curves as a function of k_{ex} in CD_2Cl_2:toluene-d_8:THF-d_8 1:1:1 and 2-methyltetrahydrofuran shown in Figures S18 and S20, respectively. The intensity of all experimental and simulated spectra was normalized before calculating the *rmsd*.

Table S4. Summary of the peak-to-peak linewidths *lwpp* resulting in the best-fit simulation of the variable temperature cw-EPR spectra of **P1**$^{\bullet+}$ in CD_2Cl_2:toluene-d_8:THF-d_8 1:1:1 with the root-mean-square deviations (*rmsd*) between the experimental and simulated spectra. The linewidths were also used for the two redox sites in the simulation of the variable temperature cw-EPR spectra of **P2**$^{\bullet+}$.

**P1**$^{\bullet+}$	**298 K**	**275 K**	**250 K**	**225 K**	**200 K**
***lwpp* / mT**	0.04	0.03	0.03	0.04	0.05
rmsd	0.0198	0.0272	0.0267	0.0329	0.0312

Table S5. Summary of the electron transfer rates k_{ex} with their lower and upper uncertainty boundaries resulting in the best-fit simulation of the variable temperature cw-EPR spectra of **P2**$^{\bullet+}$ in CD_2Cl_2:toluene-d_8:THF-d_8 1:1:1. The uncertainty boundaries of k_{ex} were determined as the electron transfer rates resulting in root-mean-square deviations (*rmsd*) 5% larger than the minimum *rmsd*.

**P2**$^{\bullet+}$	**298 K**	**275 K**	**250 K**	**225 K**	**200 K**
**k_{ex} / MHz**	53.9	40.7	23.1	12.0	10.0
rmsd	0.0110	0.0105	0.0142	0.0240	0.0440
**k_{ex} lower boundary / MHz**	47.4	36.9	20.7	9.95	6.80
rmsd	0.0116	0.0110	0.0150	0.0252	0.0462
**k_{ex} upper boundary / MHz**	61.9	45.1	25.9	14.4	14.1
rmsd	0.0116	0.0110	0.0150	0.0252	0.0462

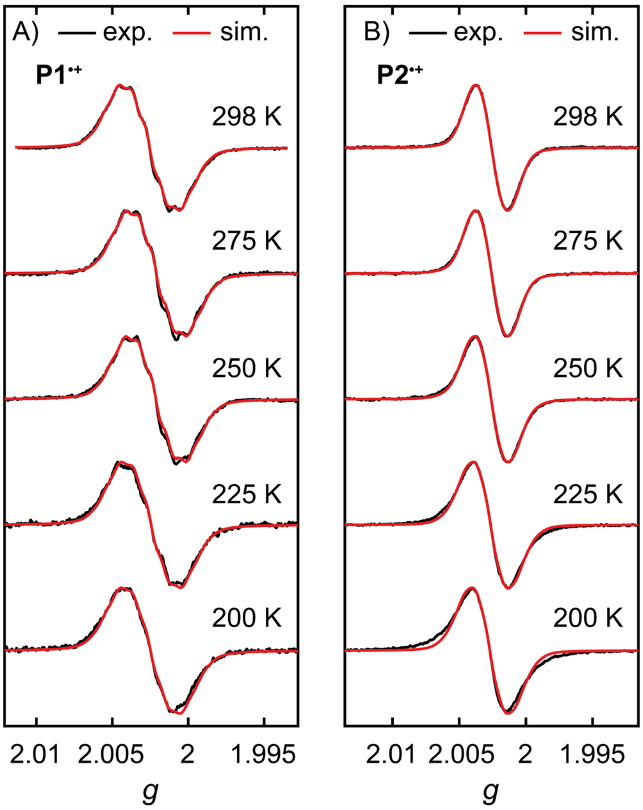

Figure S17. Comparison of the experimental (black) and simulated (red) variable temperature cw-EPR spectra of a) **P1$^{\bullet+}$** and B) **P2$^{\bullet+}$** at X-band frequencies in CD_2Cl_2:toluene-d_8:THF-d_8 1:1:1 between 298 K and 200 K. Simulations were performed with a two-site chemical exchange model using the isotropic hyperfine interactions in Table S3 and the peak-to-peak linewidths and electron transfer rates k_{ex} in Tables S4 and S5. The monomer spectra were simulated in the slow exchange limit with k_{ex} = 10^{-10} MHz.

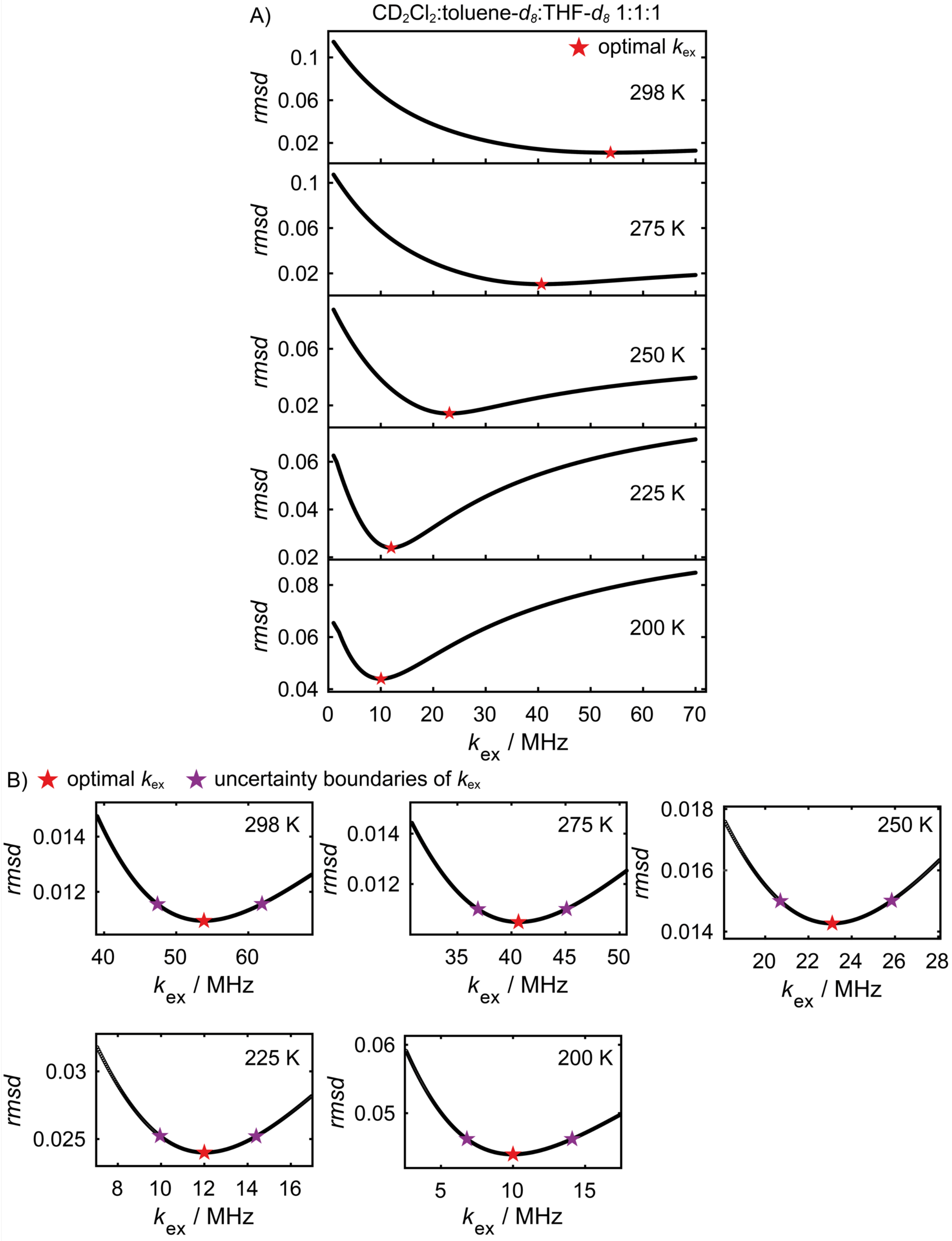

Figure S18. A) Trend in the root-mean-square deviation (*rmsd*) between the experimental and simulated cw-EPR spectra of **P2**$^{•+}$ at X-band frequencies in CD_2Cl_2:toluene-d_8:THF-d_8 1:1:1 between 298 K and 200 K as a function of the electron transfer rate k_{ex}. B) Enlarged regions of the *rmsd* curves in A) around their minima. The optimal electron transfer rates with the smallest root mean square deviations and their uncertainty boundaries with *rmsd* values 5% larger than the minimum *rmsd* are highlighted with red and purple stars, respectively.

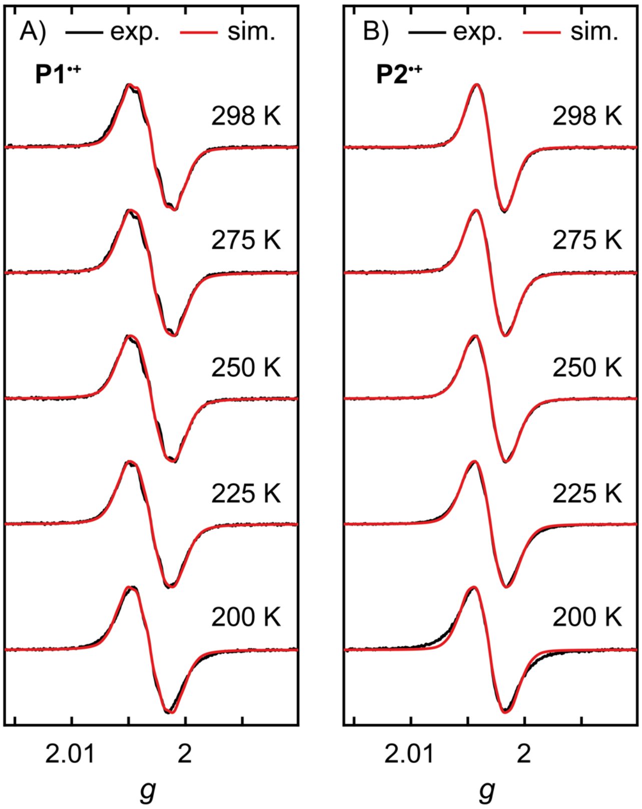

Figure S19. Comparison of the experimental (black) and simulated (red) variable temperature cw-EPR spectra of a) **P1•+** and B) **P2•+** at X-band frequencies in 2-methyltetrahydrofuran between 298 K and 200 K. Simulations were performed with a two-site chemical exchange model using the isotropic hyperfine interactions in Table S3 and the peak-to-peak linewidths and electron transfer rates k_{ex} in Tables S6 and S7. The monomer spectra were simulated in the slow exchange limit with $k_{ex} = 10^{-10}$ MHz.

Table S6. Summary of the peak-to-peak linewidths *lwpp* resulting in the best-fit simulation of the variable temperature cw-EPR spectra of **P1•+** in 2-methyltetrahydrofuran with the root-mean-square deviations (*rmsd*) between the experimental and simulated spectra. The linewidths were also used for the two redox sites in the simulation of the variable temperature cw-EPR spectra of **P2•+**.

**P1•+**	**298 K**	**275 K**	**250 K**	**225 K**	**200 K**
***lwpp* / mT**	0.04	0.07	0.08	0.08	0.05
rmsd	0.0304	0.0293	0.0286	0.0211	0.0311

Table S7. Summary of the electron transfer rates k_{ex} with their lower and upper uncertainty boundaries resulting in the best-fit simulation of the variable temperature cw-EPR spectra of **P2•+** in 2-methyltetrahydrofuran. The uncertainty boundaries of k_{ex} were determined as the electron transfer rates resulting in root-mean-square deviations (*rmsd*) 5% larger than the minimum *rmsd*.

**P2•+**	**298 K**	**275 K**	**250 K**	**225 K**	**200 K**
**k_{ex} / MHz**	27.9	22.0	14.8	10.2	7.16
rmsd	0.0113	0.0115	0.0112	0.0181	0.0422
**k_{ex} lower boundary / MHz**	25.6	20.0	13.5	8.64	4.66
rmsd	0.0118	0.0121	0.0117	0.0190	0.0443
**k_{ex} upper boundary / MHz**	30.5	24.2	16.1	11.9	10.3
rmsd	0.0118	0.0121	0.0117	0.0190	0.0443

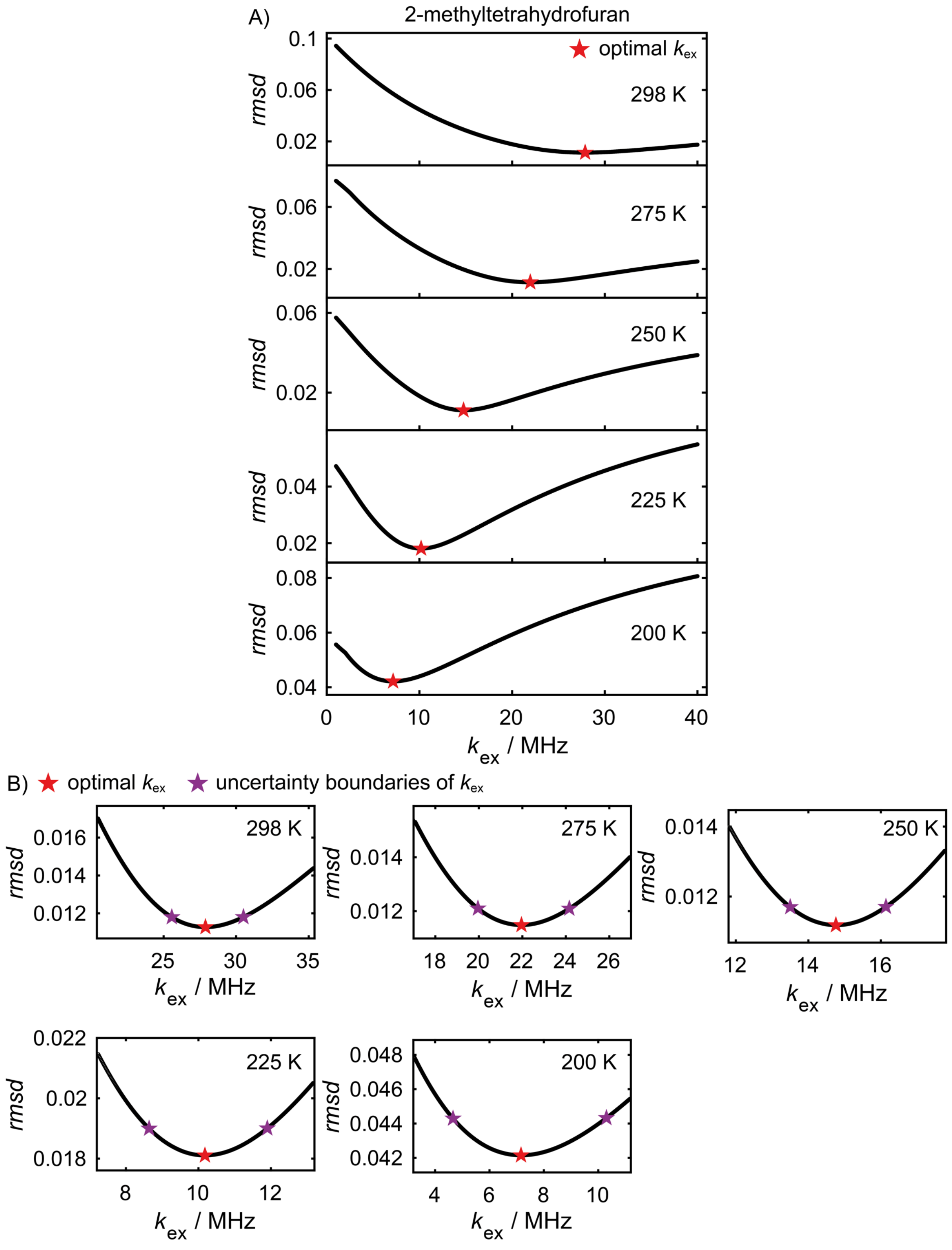

Figure S20. A) Trend in the root-mean-square deviation (*rmsd*) between the experimental and simulated cw-EPR spectra of **P2**$^{•+}$ at X-band frequencies in 2-methyltetrahydrofuran between 298 K and 200 K as a function of the electron transfer rate k_{ex}. B) Enlarged regions of the *rmsd* curves in A) around their minima. The optimal electron transfer rates with the smallest root mean square deviations and their uncertainty boundaries with *rmsd* values 5% larger than the minimum *rmsd* are highlighted with red and purple stars, respectively.

6.5 Simulation of the variable temperature cw-EPR spectra of $P1^{\bullet+}$ and $P2^{\bullet+}$ without ^{1}H hyperfine interactions

The variable temperature cw-EPR spectra of $\mathbf{P1}^{\bullet+}$ and $\mathbf{P2}^{\bullet+}$ in CD_2Cl_2:toluene-d_8:THF-d_8 1:1:1 between 298 K and 200 K were simulated analogous to the approach detailed in Section 6.1 using only four equivalent ^{14}N hyperfine interactions $^{14\mathrm{N}}A_{\mathrm{iso}}$ = 3.99 MHz. The best-fit peak-to-peak linewidths for simulating the cw-EPR spectra of $\mathbf{P1}^{\bullet+}$ and electron transfer rates k_{ex} for the simulation of $\mathbf{P2}^{\bullet+}$ are summarized in Tables S8 and S9, respectively. The root-mean-square deviation between the experimental and simulated spectra of $\mathbf{P2}^{\bullet+}$ for a range of electron transfer rates was used to determine the best simulation of the experimental spectra with the *rmsd* curves as a function of k_{ex} shown in Figure S21. If no ^{1}H hyperfine interactions are included in the simulations, substantially larger peak-to-peak linewidths are necessary to simulate the variable temperature cw-EPR spectra of $\mathbf{P1}^{\bullet+}$, resulting in an overall poorer agreement between the experimental and simulated spectra as evident from the larger *rmsd*. No reasonable fits were obtained for the variable temperature cw-EPR spectra of $\mathbf{P2}^{\bullet+}$. This highlights the importance of accounting for all hyperfine interactions in the exchange rate simulations.

Table S8. Summary of the peak-to-peak linewidths *lwpp* resulting in the best-fit simulation of the variable temperature cw-EPR spectra of $\mathbf{P1}^{\bullet+}$ in CD_2Cl_2:toluene-d_8:THF-d_8 1:1:1 with the root-mean-square deviations (*rmsd*) between the experimental and simulated spectra. Only ^{14}N hyperfine interactions were included in the simulations. The linewidths were also used for the two redox sites in the simulation of the variable temperature cw-EPR spectra of $\mathbf{P2}^{\bullet+}$.

$\mathbf{P1}^{\bullet+}$	298 K	275 K	250 K	225 K	200 K
***lwpp* / mT**	0.16	0.16	0.16	0.16	0.16
rmsd	0.0632	0.0614	0.0572	0.0469	0.0532

Table S9. Summary of the electron transfer rates k_{ex} resulting in the best-fit simulation of the variable temperature cw-EPR spectra of $\mathbf{P2}^{\bullet+}$ in CD_2Cl_2:toluene-d_8:THF-d_8 1:1:1. Only ^{14}N hyperfine interactions were included in the simulations.

$\mathbf{P2}^{\bullet+}$	298 K	275 K	250 K	225 K	200 K
**k_{ex} / MHz**	> 100000	> 100000	> 100000	218	67
rmsd	–	–	–	0.0118	0.0261

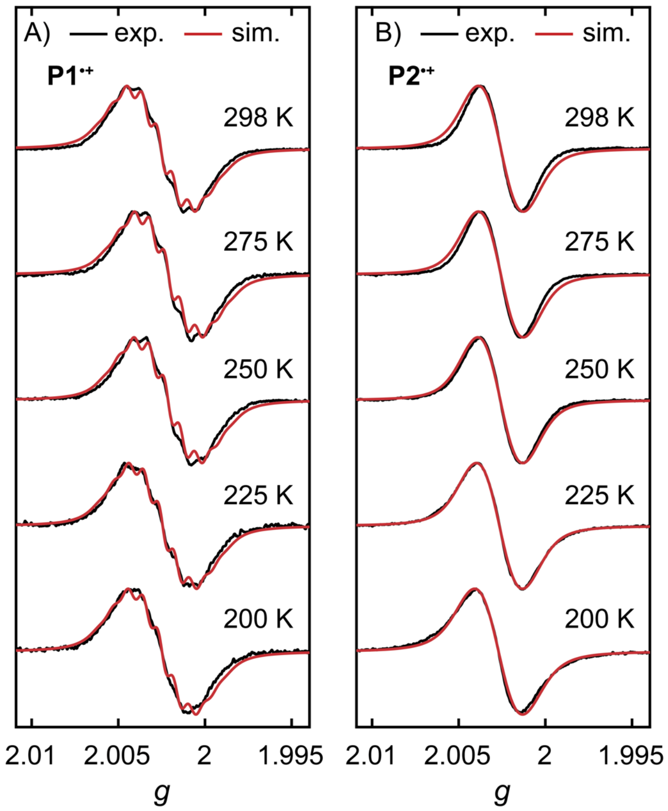

Figure S21. Comparison of the experimental (black) and simulated (red) variable temperature cw-EPR spectra of a) **P1$^{•+}$** and B) **P2$^{•+}$** at X-band frequencies in CD_2Cl_2:toluene-d_8:THF-d_8 1:1:1 between 298 K and 200 K. Simulations were performed with a two-site chemical exchange model using only isotropic ^{14}N hyperfine interactions and the peak-to-peak linewidths and electron transfer rates k_{ex} in Tables S8 and S9. The monomer spectra were simulated in the slow exchange limit with k_{ex} = 10^{-10} MHz.

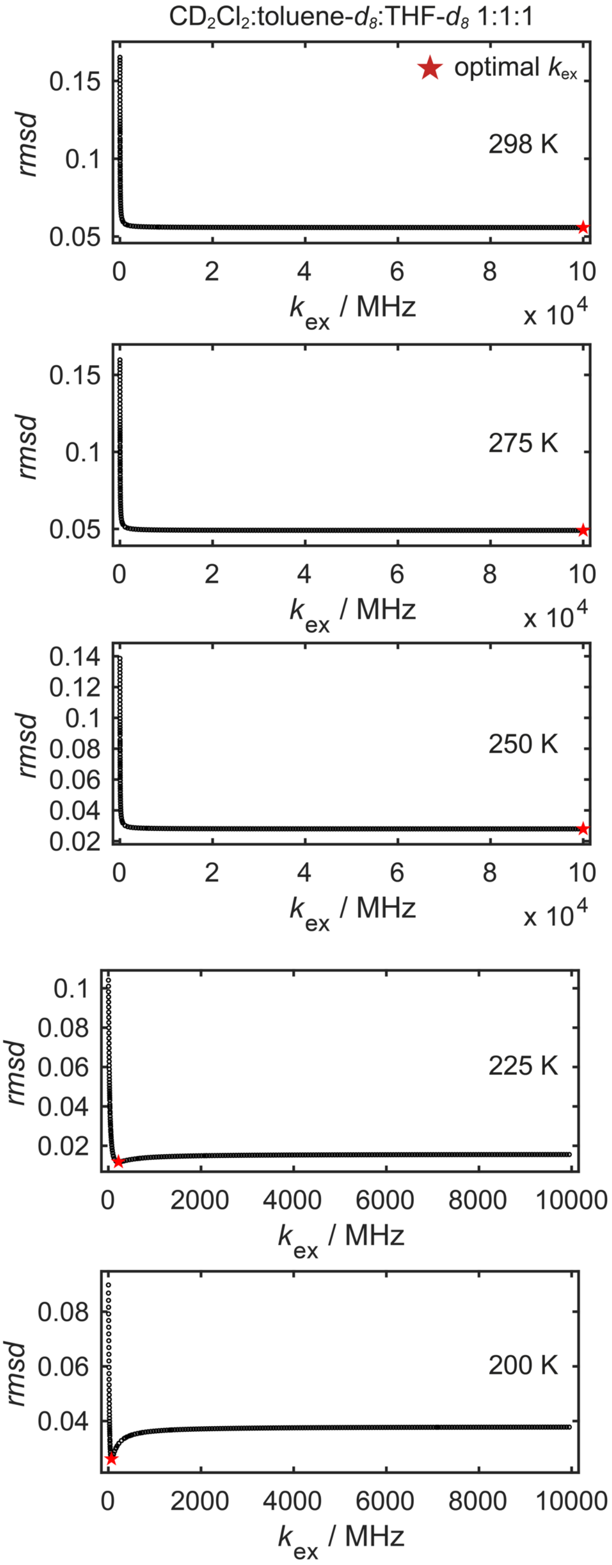

Figure S22. Trend in the root-mean-square deviation (*rmsd*) between the experimental and simulated cw-EPR spectra of **P2**$^{\bullet+}$ at X-band frequencies in CD_2Cl_2:toluene-d_8:THF-d_8 1:1:1 between 298 K and 200 K as a function of the electron transfer rate k_{ex}. Only isotropic ^{14}N hyperfine interactions are included in the simulations. The simulations show that that the variable temperature electron transfer dynamics in **P2**$^{\bullet+}$ cannot be modelled accurately without accounting for the ^{1}H hyperfine interactions.

6.6 Eyring analysis of the electron transfer kinetics

To determine the activation enthalpy $\Delta^{\ddagger}H$ and activation entropy $\Delta^{\ddagger}S$ that govern the reversible intramolecular electron transfer in **P2**$^{\bullet+}$, the temperature dependence of the electron transfer rate k_{ex} in CD_2Cl_2:toluene-d_8:THF-d_8 1:1:1 and 2-methyltetrahydrofuran was investigated using the Eyring relationship in equation 3 of the main manuscript. Plots of ln(k_{ex} T^{-1}) versus 1/T for **P2**$^{\bullet+}$ in CD_2Cl_2:toluene-d_8:THF-d_8 1:1:1 and 2-methyltetrahydrofuran are shown in Figure S23 and were used to determine $\Delta^{\ddagger}H$ and $\Delta^{\ddagger}S$ from the slope and intercept of the trendline between 298 K and 225 K, respectively. The activation enthalpy was calculated with a transmission coefficient κ = 0.035 obtained from DFT calculations (see SI Section 7.2). The error margins of $\Delta^{\ddagger}H$ and $\Delta^{\ddagger}S$ were determined from trendlines through the lower and upper uncertainty boundaries of the electron transfer rates.

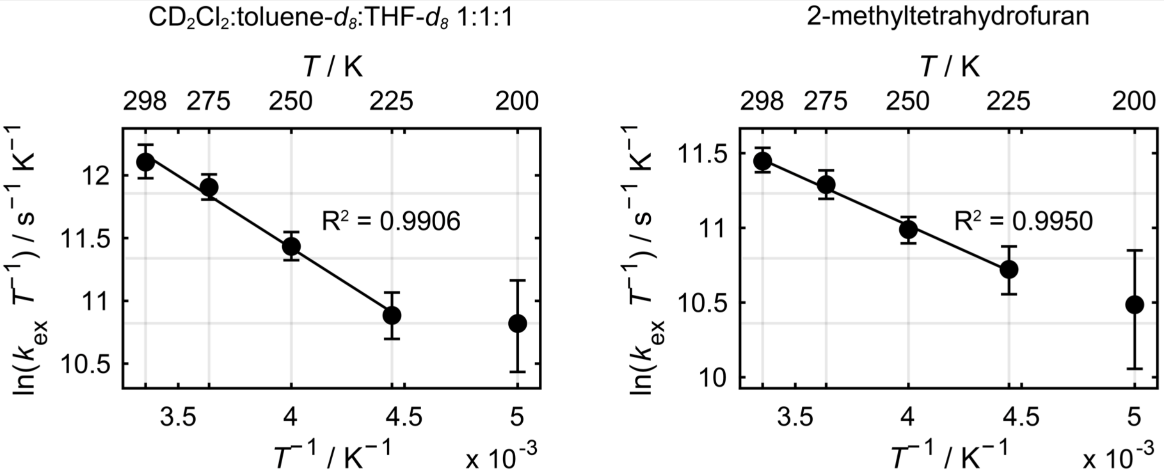

Figure S23. Eyring-equation plots of ln(k_{ex} T^{-1}) versus T^{-1} for **P2**$^{\bullet+}$ in CD_2Cl_2:toluene-d_8:THF-d_8 1:1:1 and 2-methyltetrahydrofuran. The data points at 200 K were omitted from the linear regression analysis because the experimental cw-EPR spectra at this temperature exhibit anisotropic broadening.

7. DFT Modelling

7.1 Calculation of the dihedral angle distribution in $P1^{•+}$

The relative spin density distribution Δ between the porphyrin and biphenyl parts of **P1**$^{•+}$ and therefore the hyperfine tensors ${}^{1\mathrm{H}}\boldsymbol{A}$ and ${}^{14\mathrm{N}}\boldsymbol{A}$ depend on the π-conjugation between the two segments which is modulated by their dihedral angle Φ (Figure S24A).

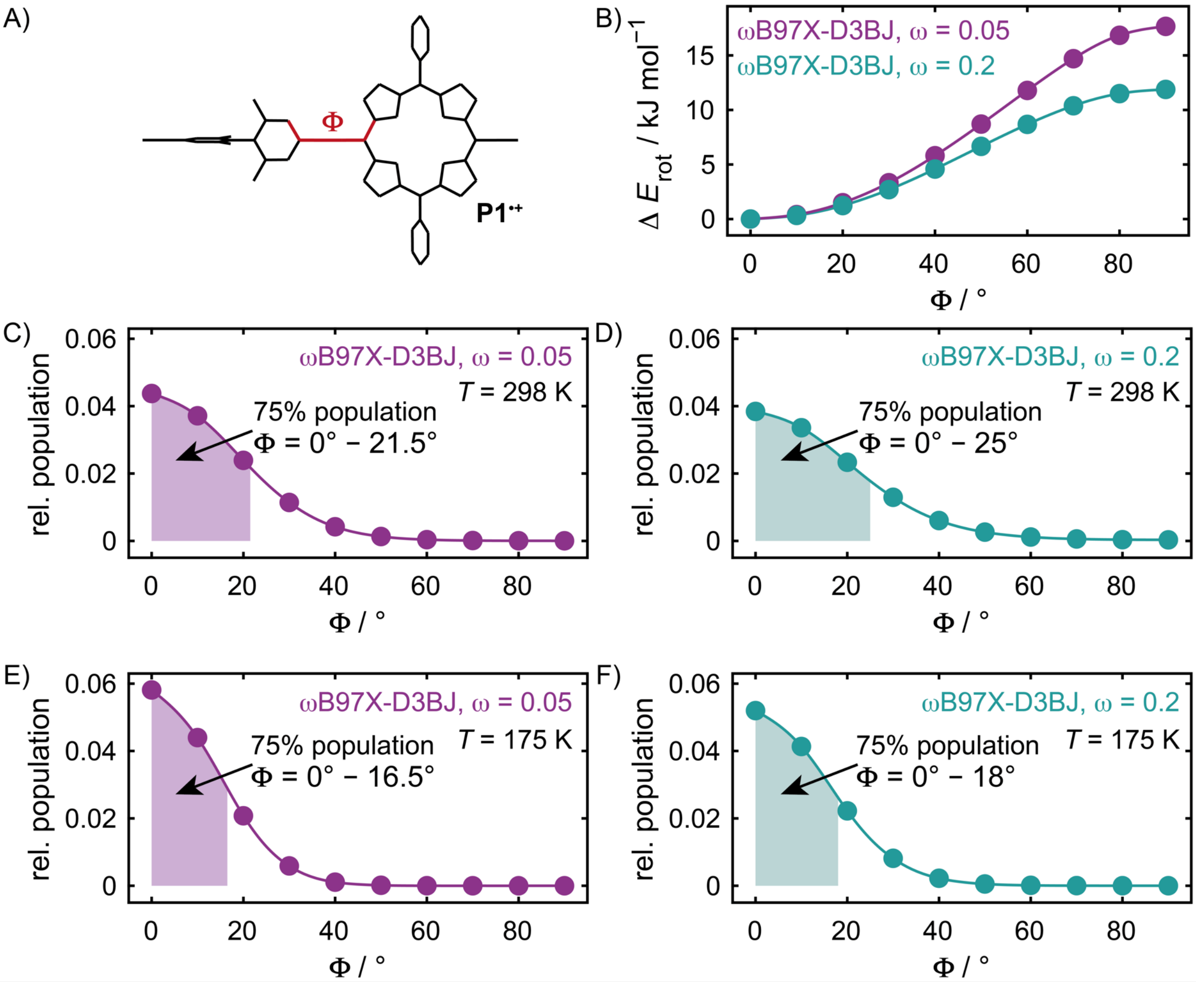

Figure S24. A) Schematic depiction of the dihedral angle Φ between the porphyrin and biphenyl parts of **P1**$^{•+}$. DFT calculated rotation barriers ΔE_{rot} for different dihedral angles Φ in **P1**$^{•+}$ calculated with ωB97X-D3BJ as functional with range-separation parameters ω = 0.05 and ω = 0.2 and the 6-31G* basis set. C)–F) Boltzmann weighted distributions of dihedral angles in **P1**$^{•+}$ at 298 K and 175 K obtained from the DFT calculated rotation barriers. The shaded areas indicate the dihedral angles that account for 75% of the populated angles. DFT calculations for the different geometries of **P1**$^{•+}$ with constrained dihedral angles were performed in 10° steps (indicated by the solid markers) and interpolated to obtain the trends presented here.

To determine if different dihedral angle distributions need to be taken into consideration for the analysis of **P1**$^{•+}$, the rotation barrier ΔE_{rot} between the biphenyl and porphyrin parts was calculated for a range of dihedral angles Φ following equation S11

$$\Delta E_{\mathrm{rot}} = E(\Phi) - E(0°) \tag{S11}$$

where $E(\Phi)$ is the energy of the DFT optimized structure of **P1**$^{•+}$ with a constrained dihedral angle Φ and $E(0°)$ is the energy minimum corresponding to the optimized structure with Φ = 0°. The geometry optimizations of **P1**$^{•+}$ were performed with ωB97X-D3BJ as functional with range separation parameters ω = 0.05 and ω =0.2 and the 6-31G* basis set to account for different extents of radical delocalization

from the porphyrin onto the biphenyl fragment in the subsequent analysis. Larger rotation barriers ΔE_{rot} were obtained with ω = 0.05 as range separation parameter (Figure S24B).

Using the Boltzmann relationship in equation S12, the population ratio of dihedral angles was calculated at 298 K and 175 K, just above the glass formation temperature of CD_2Cl_2:toluene-d_8:THF-d_8 1:1:1, using the DFT calculated rotation barriers.

$$\frac{P(\Phi)}{P(0°)} = \mathrm{e}^{\frac{-\Delta E_{\mathrm{rot}}}{RT}} \tag{S12}$$

The relative populations were subsequently normalized following equation S13 and are shown in Figure S24C–F. The integration was performed using the trendlines obtained from interpolation of the DFT calculated values.

$$\text{relative population } (\Phi) = \frac{P(\Phi)/P(0°)}{\int_{\Phi=0°}^{\Phi=90°} P(\Phi)/P(0°)} \tag{S13}$$

The relative population of dihedral angles varies slightly depending on the range separation parameter employed in the DFT calculations but gives a similar qualitative picture of the thermal population of Φ. At 298 K, the dihedral angles between Φ = 0° – 21.5° using ω = 0.05 as range separation parameter and Φ = 0° – 25° for ω = 0.2 account for 75% of the populated dihedral angles (Figure S24C+D). This distribution is slightly narrowed to Φ = 0° – 16.5° for ω = 0.05 and Φ = 0° – 18° for ω = 0.2 at 175 K which is representative of the dihedral angle distribution in frozen CD_2Cl_2:toluene-d_8:THF-d_8 1:1:1 (Figure S24E+F). An increased angle Φ from 0° to 20° only increases the DFT predicted relative spin density distribution Δ by 1 % for both range separation parameters and therefore at most results in a slight change during the assignment of the optimal spin density distribution in **P1**$^{\bullet+}$ using the point-dipole model in Section 4. While the different geometries of **P1**$^{\bullet+}$ might result in changes to the anisotropic hyperfine couplings due to different dipolar interactions, this will likely not affect the experimental spectra beyond a slight broadening of their transitions as only a small fraction of dihedral angles substantially deviates from the coplanar arrangement of the porphyrin and biphenyl units. This suggests that simulations based on a single geometry with Φ = 0° are sufficient to model the experimental spectra of **P1**$^{\bullet+}$.

7.2 Calculation of the orbital overlap in P2$^{\bullet+}$

The rate of the electron transfer k_{ex} in **P2**$^{\bullet+}$ is determined by the overlap integral S_{AB} between Sites A and B of the two-state chemical exchange process that are characterized by the distribution of the radical spin density over one half or the other half of **P2**$^{\bullet+}$ (Figure S25). In the equilibrium geometry of **P2**$^{\bullet+}$, the two phenyls of the tetramethylbiphenyl linker are virtually orthogonal ($\Theta_{eq} \approx 90°$), resulting in a low overlap S_{AB} between the two symmetric charge-localized states.

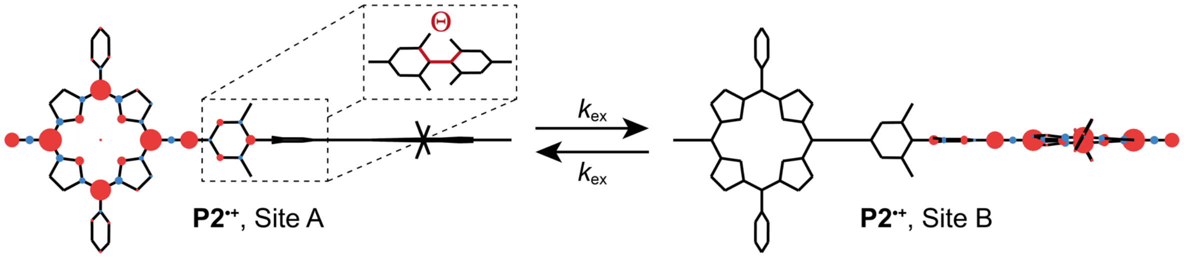

Figure S25. Schematic visualization of the redistribution of spin density during the reversible intramolecular electron transfer in **P2**$^{\bullet+}$. The spin population on each nucleus is represented by a sphere centered on the atom with a radius proportional to the magnitude of its assigned spin population. Red spheres represent excess spin-up and light-blue spheres indicate excess spin-down populations. The torsion angle Θ between the phenyl parts of the tetramethylbiphenyl linker is shown in the insert.

Changes in the torsional angle Θ, which occur through a vibration with the nuclear frequency ν_n = 90 cm^{-1} increase S_{AB} and thus also the rate of electron transfer k_{ex}. The degree of coupling between electron and nuclear frequencies k_{ex} and ν_n can be quantified by calculating the electron transmission coefficient κ using the Landau-Zener approach:[15,16]

$$\kappa = 1 - \frac{\exp(-2\pi\gamma)}{2-\exp(-2\pi\gamma)} \tag{S14}$$

where γ is the adiabatic parameter calculated in equation S15 from the electronic coupling H_{AB} and the reorganization energy λ.

$$\gamma = \frac{{H_{AB}}^2}{2h\nu_n}\sqrt{\frac{\pi}{\lambda k_B T}} \tag{S15}$$

An electron transfer in the adiabatic regime results from a large overlap S_{AB} between the two states leading to electron transfer rates faster than the nuclear motion (k_{ex} >> ν_n), a large adiabatic parameter γ (γ >> 1) and a transmission coefficient $\kappa \approx 1$. In the nonadiabatic regime with a small overlap S_{AB} the electron transfer proceeds slower than the nuclear motions ($k_{ex} < \nu_n$) and is characterized by a small adiabatic parameter (γ << 1) and transmission coefficient ($\kappa < 1$).

All parameters in equation S15 can be estimated using DFT. Following Gajdos, we determine the electronic coupling between the two charge-localized states H_{AB} from their frontier orbital overlap (H_{AB} = 3.5 eV S_{AB}).[17] We estimate the reorganization energy (λ = 0.19 eV) from radical cation and neutral energies,[18] and consider only the coupling between the torsion (ν_n = 90 cm^{-1}) and the electron transfer. With these approximations, we obtain $\kappa \approx 0.035$, strongly indicating that electron transfer occurs in the nonadiabatic regime. The transmission coefficient κ obtained from DFT calculations in essentially temperature independent between 200 K and 298 K.

As the temperature increases, a broader range of Θ becomes available, increasing the rate of charge transfer k_{ex}, which can be qualitatively accounted for using Marcus theory:[19,20]

$$k_{ex} = \frac{{H_{AB}}^2}{\hbar}\sqrt{\frac{\pi}{\lambda k_B T}}\exp\left(-\frac{\lambda}{4k_B T}\right) \tag{S16}$$

In equation S16, H_{AB} is calculated from the expectation value of Θ for each temperature, which produces a qualitatively correct rate dependance with temperature, even though only a single vibration is considered. All calculations were done at the LC-ωPBE/def2-SVP level of theory.[21]

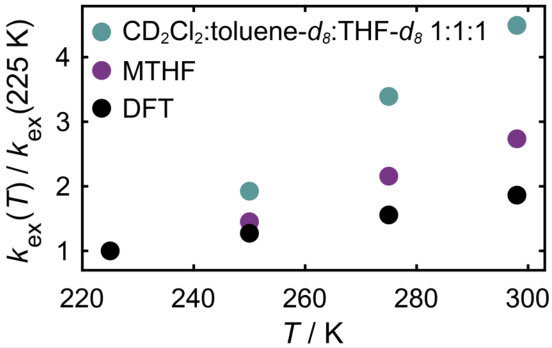

Figure S26. Temperature dependence of the experimental relative charge transfer rates k_{ex} in CD_2Cl_2:toluene-d_8:THF-d_8 1:1:1 (green) and 2-methyltetrahydrofuran (purple) and the predicted transfer rates using Marcus equation (black).

8. Absorption Spectroscopy and TD-DFT Calculations

The UV-vis-NIR absorption spectra of **P1$_{Sym}$**$^{•+}$, **P1**$^{•+}$, and **P2**$^{•+}$ were obtained from chemical oxidation titrations in $CHCl_3$ with BAHA (Figure S27). The titrations were performed under ambient atmosphere by stepwise addition of BAHA (in CH_2Cl_2) to the porphyrin solutions in a quartz glass cuvette.

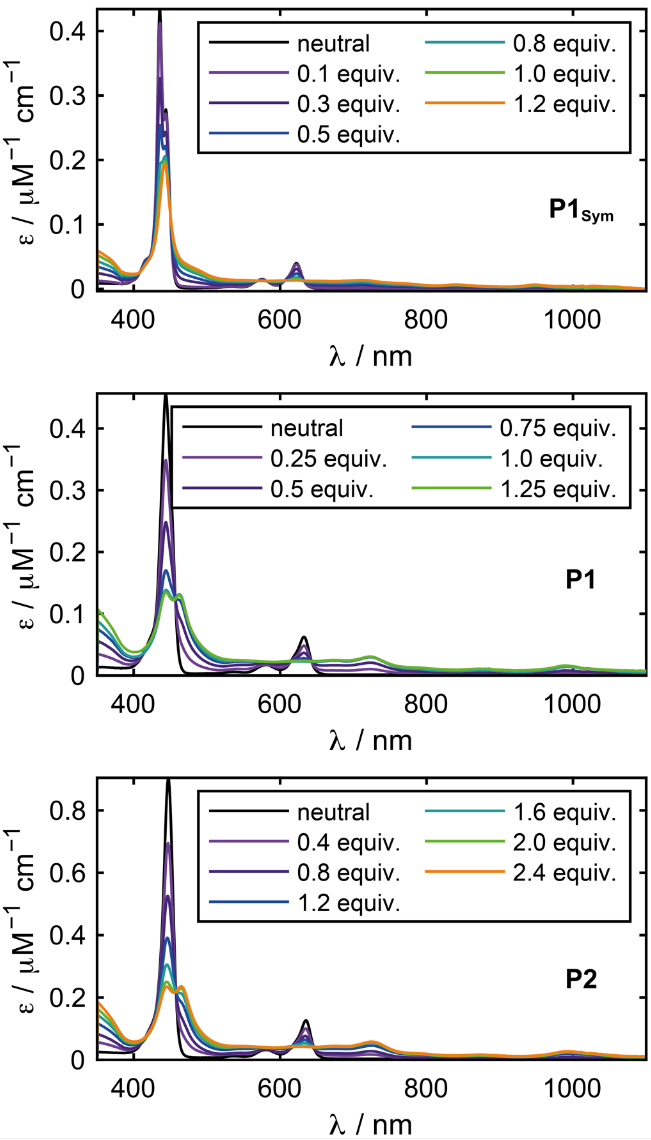

Figure S27. UV-vis-NIR absorption spectra of the oxidation titrations of **P1$_{Sym}$**, **P1**, and **P2** in $CHCl_3$ with BAHA. The oxidant was added to the porphyrin solutions from a stock solution in CH_2Cl_2 due to its limited solubility in $CHCl_3$.

Analogous oxidation titrations of **P1$_{Sym}$**, **P1**, and **P2** with a larger detection window in the near infrared (1000–2500 nm) confirm that no intervalence charge-transfer band (IV-CT) is observed experimentally for **P2**$^{•+}$ (Figure S28).

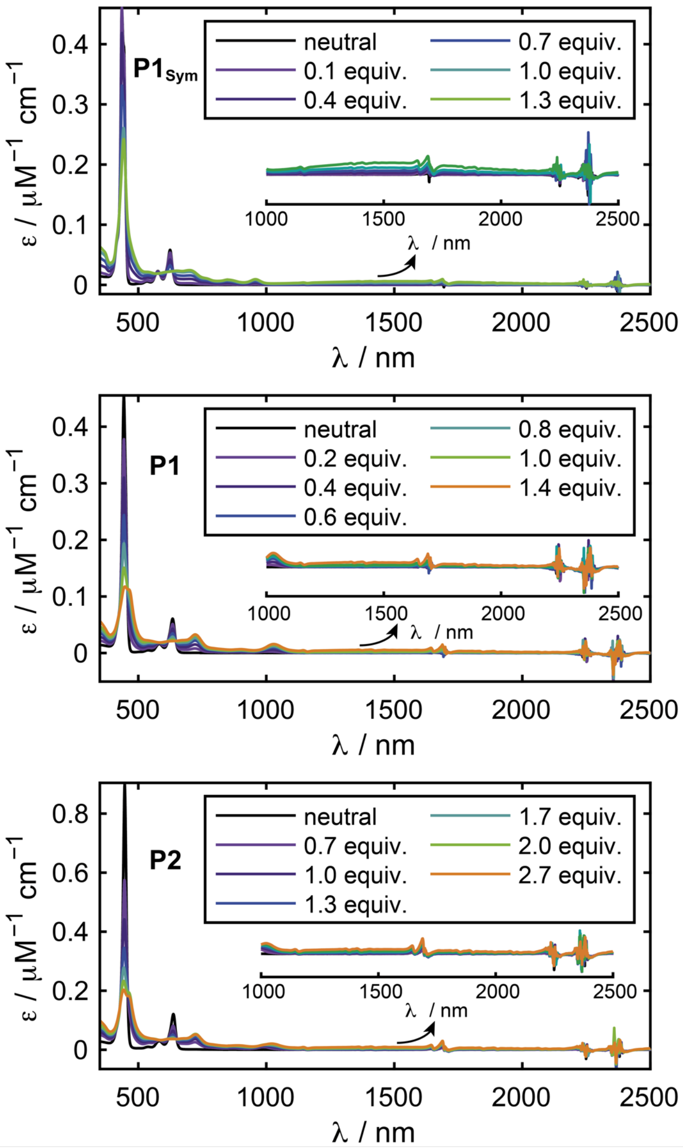

Figure S28. UV-vis-NIR absorption spectra of the oxidation titrations of **P1$_{Sym}$**, **P1**, and **P2** in CH_2Cl_2 with BAHA.

The TD-DFT calculations of neutral and oxidized **P1$_{Sym}$**, **P1**, and **P2** were performed on their optimized geometries using the ORCA software package with LC-ωPBE (ω= 0.1) as functional and the 6-31G* basis set with the conductor-like polarizable continuum model (CPCM) with chloroform as solvent. The calculated absorption spectra of **P1$_{Sym}$**, **P1$_{Sym}^{•+}$**, **P1**, **P1$^{•+}$**, **P2**, and **P2$^{•+}$** are shown in Figure S29 and the individual transitions and oscillator strengths are summarized in Tables S10–S15.

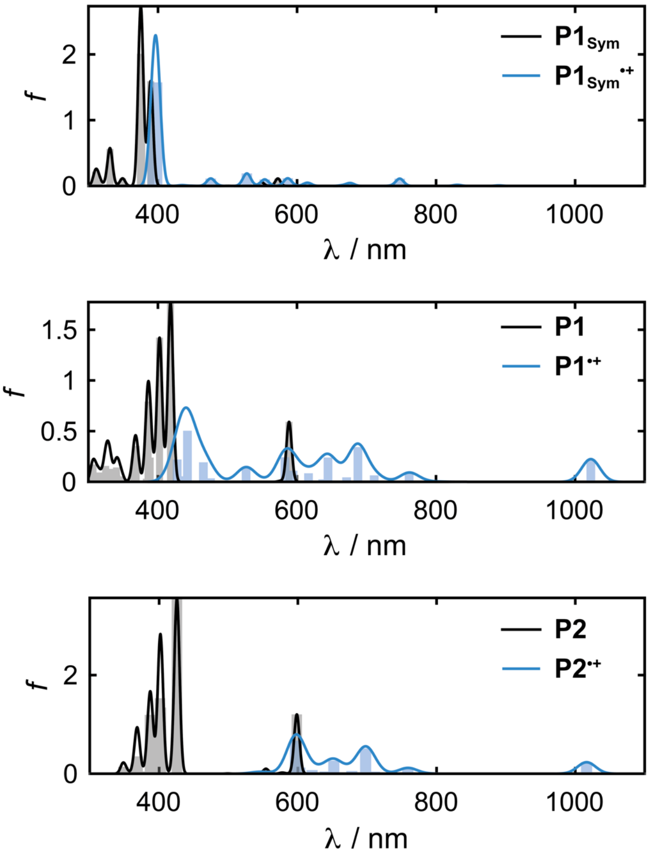

Figure S29. TD-DFT calculated absorption spectra of neutral (black) and oxidized (blue) **P1$_{Sym}$**, **P1**, and **P2** in chloroform using LC-ωPBE, ω =0.1 as functional with the 6-31G* basis set.

Table S10. TD-DFT excitation wavelengths λ, excitation energies E_{abs}, and oscillator strengths f for **P1$_{Sym}$** in $CHCl_3$ calculated with LC-ωPBE (ω= 0.1) as functional and the 6-31G* basis set.

**P1$_{Sym}$**, excited state	λ / nm	E_{abs} / eV	f
1	572	2.17	0.1129
2	564	2.20	0.0047
3	549	2.26	0.0582
4	500	2.48	0.0024
5	389	3.18	1.5917
6	387	3.20	0.0000
7	385	3.22	0.0000
8	377	3.29	0.7996
9	375	3.31	2.0081
10	361	3.43	0.0000
11	349	3.55	0.1148
12	345	3.59	0.0000
13	344	3.61	0.0002
14	343	3.62	0.0000
15	341	3.64	0.0077
16	331	3.75	0.5620
17	330	3.76	0.0000
18	327	3.79	0.0205
19	326	3.80	0.0000
20	324	3.83	0.0078
21	324	3.83	0.0000
22	323	3.83	0.0008
23	315	3.93	0.0006
24	315	3.93	0.0902
25	313	3.96	0.0000
26	310	4.00	0.2173
27	308	4.02	0.0000
28	308	4.02	0.0008
29	300	4.13	0.0006
30	281	4.41	0.0066
31	281	4.41	0.0001
32	280	4.43	0.0000

Table S11. TD-DFT excitation wavelengths λ, excitation energies E_{abs}, and oscillator strengths f for **P1$_{Sym}$$^{•+}$** in $CHCl_3$ calculated with LC-ωPBE (ω= 0.1) as functional and the 6-31G* basis set.

**P1$_{Sym}$$^{•+}$**, excited state	λ / nm	E_{abs} / eV	f
1	2384	0.52	5.2000E-08
2	1362	0.91	0.0000E+00
3	890	1.39	8.4303E-03
4	831	1.49	1.6984E-02
5	828	1.50	2.0763E-05
6	748	1.66	1.1054E-01
7	736	1.69	3.5205E-03
8	694	1.79	0.0000E+00
9	677	1.83	4.3374E-02
10	670	1.85	6.5016E-04
11	665	1.86	8.8577E-03
12	650	1.91	0.0000E+00
13	615	2.02	4.9011E-02
14	587	2.11	1.1653E-01
15	554	2.24	9.8839E-02
16	528	2.35	1.9027E-01
17	509	2.44	0.0000E+00
18	506	2.45	2.0860E-05
19	485	2.56	4.2372E-03
20	484	2.56	3.6991E-04
21	483	2.57	0.0000E+00
22	476	2.61	1.1217E-01
23	448	2.77	3.3983E-03
24	439	2.82	0.0000E+00
25	435	2.85	1.2145E-02
26	433	2.86	1.6070E-05
27	409	3.03	3.2380E-06
28	407	3.05	1.1487E-02
29	405	3.06	0.0000E+00
30	399	3.11	1.5739E+00
31	395	3.14	1.6570E-06
32	393	3.16	1.0495E+00

Table S12. TD-DFT excitation wavelengths λ, excitation energies E_{abs}, and oscillator strengths f for **P1** in $CHCl_3$ calculated with LC-ωPBE (ω= 0.1) as functional and the 6-31G* basis set.

**P1**, excited state	λ / nm	E_{abs} / eV	f
1	589	2.11	0.5915
2	576	2.15	0.0097
3	558	2.22	0.0001
4	500	2.48	0.0000
5	418	2.97	1.7724
6	402	3.08	1.4213
7	389	3.19	0.2390
8	386	3.21	0.7908
9	383	3.24	0.0305
10	369	3.36	0.3531
11	365	3.39	0.1428
12	353	3.52	0.0000
13	347	3.57	0.0379
14	346	3.58	0.0539
15	345	3.59	0.0007
16	341	3.64	0.0645
17	340	3.64	0.1419
18	339	3.65	0.0033
19	339	3.66	0.0003
20	331	3.75	0.1280
21	331	3.75	0.0620
22	328	3.78	0.1061
23	327	3.79	0.0096
24	327	3.79	0.0104
25	325	3.82	0.1570
26	324	3.83	0.0444
27	318	3.90	0.0032
28	315	3.94	0.0924
29	313	3.96	0.0146
30	309	4.02	0.0434
31	308	4.02	0.0001
32	307	4.04	0.1711

Table S13. TD-DFT excitation wavelengths λ, excitation energies E_{abs}, and oscillator strengths f for **P1**$^{•+}$ in $CHCl_3$ calculated with LC-ωPBE (ω= 0.1) as functional and the 6-31G* basis set.

**P1**$^{•+}$, excited state	λ / nm	E_{abs} / eV	f
1	2063	0.60	0.0006
2	1452	0.85	0.0000
3	1023	1.21	0.2243
4	806	1.54	0.0018
5	788	1.57	0.0000
6	781	1.59	0.0068
7	761	1.63	0.0899
8	711	1.74	0.0640
9	703	1.76	0.0000
10	688	1.80	0.3427
11	671	1.85	0.0445
12	645	1.92	0.2395
13	637	1.95	0.0245
14	634	1.96	0.0001
15	633	1.96	0.0003
16	617	2.01	0.0847
17	594	2.09	0.1098
18	583	2.13	0.2423
19	570	2.18	0.0000
20	547	2.27	0.0054
21	527	2.35	0.1447
22	502	2.47	0.0000
23	502	2.47	0.0000
24	483	2.57	0.0002
25	476	2.61	0.0346
26	465	2.66	0.1926
27	461	2.69	0.0001
28	443	2.80	0.5038
29	439	2.82	0.0541
30	437	2.84	0.0078
31	435	2.85	0.0045
32	428	2.90	0.2204

Table S14. TD-DFT excitation wavelengths λ, excitation energies E_{abs}, and oscillator strengths f for **P2** in $CHCl_3$ calculated with LC-ωPBE (ω= 0.1) as functional and the 6-31G* basis set.

**P2**, excited state	λ / nm	E_{abs} / eV	f
1	599	2.07	1.2027
2	588	2.11	0.0035
3	578	2.14	0.0112
4	577	2.15	0.0111
5	554	2.24	0.0764
6	553	2.24	0.0256
7	499	2.48	0.0021
8	499	2.49	0.0025
9	426	2.91	3.5655
10	411	3.01	0.0001
11	411	3.02	0.0000
12	410	3.02	0.0034
13	403	3.08	1.3426
14	401	3.09	1.5303
15	389	3.19	0.2373
16	389	3.19	0.2443
17	387	3.21	1.1929
18	385	3.22	0.0128
19	384	3.23	0.0305
20	383	3.24	0.0440
21	369	3.36	0.3324
22	369	3.36	0.3520
23	367	3.38	0.1956
24	366	3.39	0.0932
25	361	3.43	0.0000
26	360	3.45	0.0000
27	354	3.50	0.0310
28	352	3.52	0.0000
29	349	3.56	0.1393
30	348	3.57	0.0765
31	346	3.59	0.0013
32	345	3.59	0.0001

Table S15. TD-DFT excitation wavelengths λ, excitation energies E_{abs}, and oscillator strengths f for **P2**$^{•+}$ in $CHCl_3$ calculated with LC-ωPBE (ω= 0.1) as functional and the 6-31G* basis set.

**P2**$^{•+}$, excited state	λ / nm	E_{abs} / eV	f
1	2033	0.61	0.0006
2	1508	0.82	0.0000
3	1237	1.00	0.0076
4	1016	1.22	0.2328
5	910	1.36	0.0000
6	859	1.44	0.0000
7	799	1.55	0.0006
8	776	1.60	0.0042
9	759	1.63	0.1161
10	713	1.74	0.0000
11	711	1.74	0.0413
12	707	1.75	0.0001
13	702	1.77	0.0000
14	698	1.78	0.5138
15	689	1.80	0.0000
16	678	1.83	0.0543
17	676	1.83	0.0000
18	652	1.90	0.2661
19	640	1.94	0.0441
20	634	1.95	0.0009
21	631	1.97	0.0000
22	621	2.00	0.0726
23	603	2.06	0.1228
24	597	2.08	0.6690
25	594	2.09	0.0000
26	588	2.11	0.0000
27	580	2.14	0.0042
28	571	2.17	0.0000
29	563	2.20	0.0000
30	546	2.27	0.0203
31	545	2.27	0.0155
32	544	2.28	0.0039

9. Transient EPR of Photogenerated Triplet States

<u>Basic optical characterization:</u>

Steady-state UV–vis absorption spectra of **P1$_{Sym}$**, **P1**, and **P2**, prepared as toluene solutions, were recorded at room temperature using a Cary60 UV–vis spectrophotometer, Figure S30. Emission spectra were recorded using an Edinburgh Instruments FS5 Fluorimeter. All emission data were acquired using dilute solutions with a Soret band maximum steady-state absorption of ≈50 mOD. The steady-state excitation spectra (730 nm detection) and steady-state emission spectra (450 nm excitation) for **P1** and **P2** are presented in Figure S30. Time-resolved fluorescence decay traces were recorded for **P1** and **P2** using the time-correlated single-photon counting method and are presented in Figure S31, alongside the instrument response. The decay curves were analyzed using the Edinburgh Instruments FAST software package, the kinetics of the fluorescence decay are independent of the chosen emission wavelength and consists of a single exponential with a lifetime of around 1.5 ns, for both **P1** and **P2**.

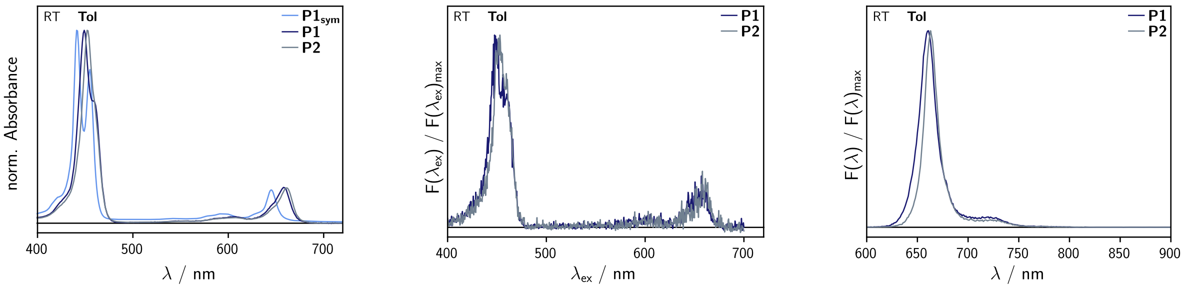

Figure S30. Comparison of the room temperature steady-state UV–vis absorption spectra of **P1$_{Sym}$**, **P1**, and **P2** (*left*), steady-state excitation spectra of **P1** and **P2** with emission detected at 730 nm (*middle*), and steady-state fluorescence emission spectra for **P1** and **P2** following excitation at 450 nm (*right*).

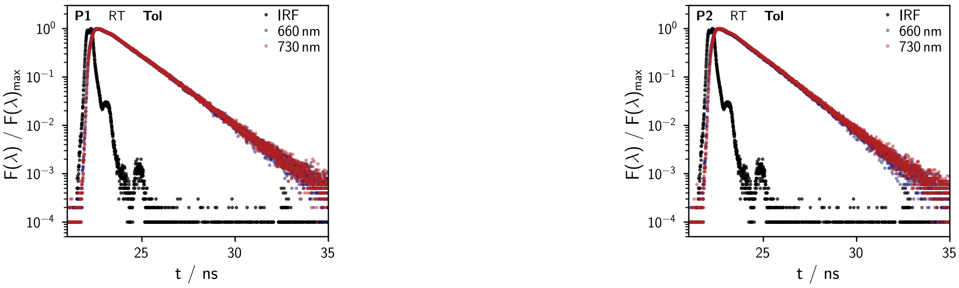

Figure S31. Fluorescence decay traces recorded at different emission wavelengths, as indicated, for **P1** and **P2** following photoexcitation at 450 nm.

<u>EPR acquisition parameters:</u>

Samples for transient EPR spectroscopy were prepared to a concentration of 150 μM in 2-methyltetrahydrofuran in X-band EPR tubes (3.8 mm o.d.). For the doped PVK-film preparation, stock solutions of PVK and porphyrin were prepared in toluene at 20 mg mL^{-1} and 1.5 mg mL^{-1}, respectively. The PVK stock solution was stirred at 40 °C until it was dissolved. The porphyrin and PVK stock solutions were combined in a ratio of 1:5 in an X-band EPR tube. The solvent was removed in vacuo to create a film on the inside of the tube.

Photoexcitation of **P1$_{Sym}$**, **P1**, and **P2** was performed with depolarized light at 532 nm using either an Opotek Opolette He 355 LD optical parametric oscillator pumped by the third harmonic of a ND:YAG LASER or a Continuum Minilite Nd:YAG. The excitation energy was ~1–2 mJ/pulse at a repetition rate of 20 Hz or 15 Hz. The LASER light was directed to the sample directly though the optical window of the cryostat. The X-band transient EPR spectra were recorded at 20 K and 250 K on a X-/Q-band Bruker Elexsys 580 spectrometer equipped with a helium gas flow cryostat from Oxford Instruments with a microwave power of 0.2–1.5 mW. The proton hyperfine interactions of the photogenerated triplet

states were investigated at 20 K using transient Mims ENDOR spectroscopy with $t_{\pi/2}$ = 16 ns and t_{rf} = 15 μs or 18 μs. All ^{1}H ENDOR spectra were acquired with three different τ-values (τ = 120 ns, 180 ns, and 240 ns or 150 ns, 200 ns, and 250 ns) to rule out distortions arising from the blind-spot characteristics of the Mims ENDOR sequence. All ENDOR spectra were obtained by LASER excitation at 532 nm.

The experimental continuous wave transient EPR spectra were baseline corrected in both dimensions using the pre-LASER spectrum and the off-resonant transients. Simulations of the spin-polarized triplet state spectra were performed using the EasySpin function pepper.[9]

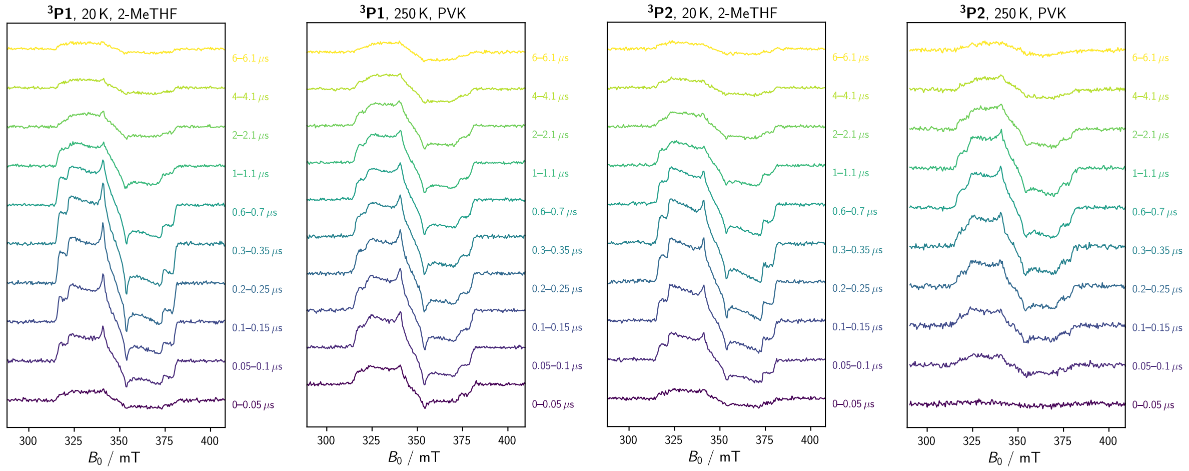

Figure S32. TrEPR time-slice spectra, averaged for several delay after LASER excitation timegates (as indicated), for **P1** and **P2** at 20 K for toluene solution and 250 K for doped PVK film.

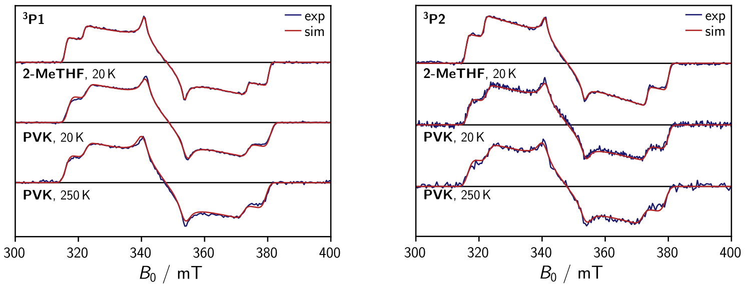

Figure S33. Experimental time-slice (300–400 ns) trEPR spectra for **P1** (*left*) and **P22** (*right*) prepared as either a toluene solution or a doped PVK film recorded at either 20 K or 250 K, as indicated. Numerical simulations, performed in MATLAB using the EasySpin pepper function, are presented alongside the experimental data, relavant simulation parameters are presented in Table S16.

Table S16. trEPR simulation parameters: ZF interaction parameters (D and η_D) and relative triplet sublevel populations. The sublevel populations have been rescaled and normalized such that the smallest value is zero and the sum equals unity.

	D / MHz	η_D	P_X	P_Y	P_Z
^{3}**P1,** MTHF, 20 K	904	0.2	0.07	0	0.93
^{3}**P1,** PVK, 20 K	894	0.20	0.11	0	0.89
^{3}**P1,** PVK, 250 K	891	0.19	0.10	0	0.90
^{3}**P2,** MTHF, 20 K	900	0.2	0	0.05	0.95
^{3}**P2,** PVK, 20 K	886	0.20	0.06	0	0.94
^{3}**P2,** PVK, 250 K	875	0.19	0.04	0	0.96

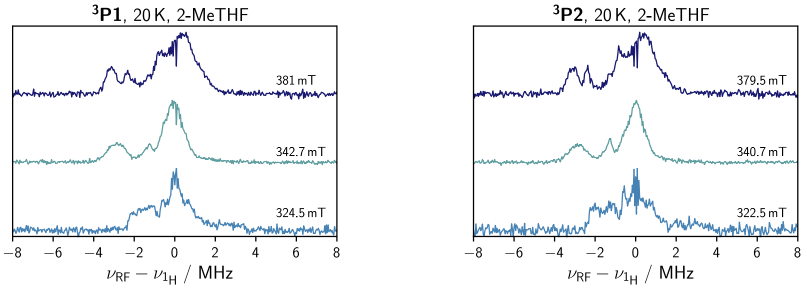

Figure S34. Experimental ^{1}H Mims ENDOR spectra of **P1** and **P2**, *left* and *right*, respectively. For each systems the Mims ENDOR spectra were acquired at three magnetic-field positions, corresponding to the Z^-, Y^-, and X^- canonical orientation triplet transitions for *top*, *middle*, and *bottom*, respectively.

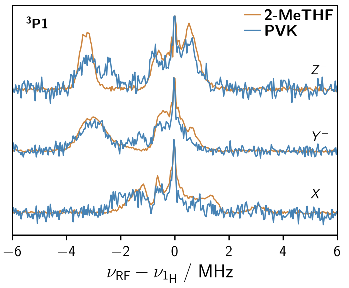

Figure S35. Experimental ^{1}H Mims ENDOR spectra recorded at 20 K for **P1** prepared as either a 2-methyltetrahydrofuran solution or a PVK doped film, *left* and *right*, respectively. For each systems the Mims ENDOR spectra were acquired at three magnetic-field positions, corresponding to the Z^-, Y^-, and X^- canonical orientation triplet transitions for *top*, *middle*, and *bottom*, respectively.

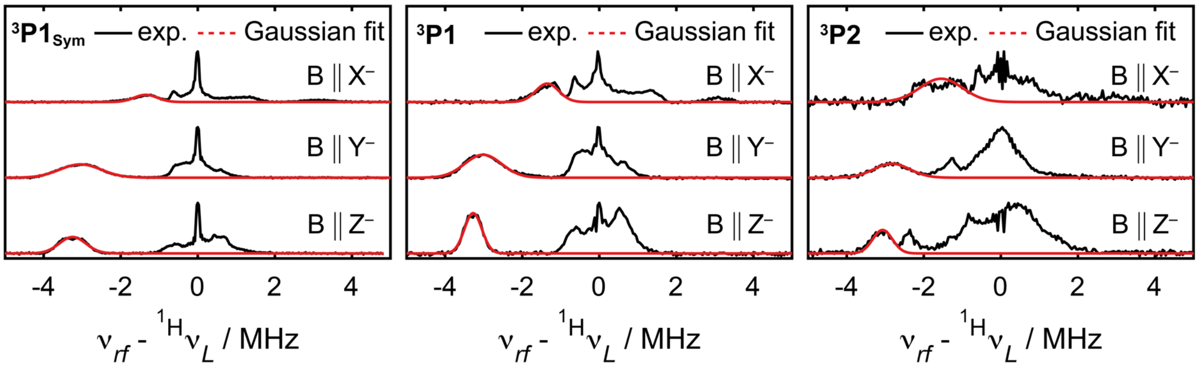

Figure S36. Experimental ^{1}H Mims ENDOR spectra of **^{3}P1$_{Sym}$**, **^{3}P1**, and **^{3}P2** recorded at the X⁻, Y⁻, and Z⁻ field positions at 20 K in 2-methyltetrahydrofuran at X-band frequencies. The largest negative hyperfine couplings along the X, Y, and Z axes of the zero-field splitting tensor were determined by fitting a Gaussian to the principal hyperfine peak of the ENDOR spectra with the distribution of the hyperfine interactions determined from the full width at half maximum of the Gaussian. The fitted values are given in Table S17.

Table S17. Summary of the largest negative hyperfine couplings ${}^{1\mathrm{H}}A$ along the X, Y, and Z directions of the zero field splitting tensors in **^{3}P1$_{Sym}$**, **^{3}P1**, and **^{3}P2** determined from fitting a Gaussian to the principal hyperfine peaks. The distribution of the hyperfine interactions was determined from the full width at half maximum of the Gaussian.

	**^{3}P1$_{Sym}$**	**^{3}P1**	**^{3}P2**
${}^{1\mathrm{H}}A_X$ / MHz	−1.35 ± 0.35	−1.35 ± 0.35	−1.55 ± 0.65
${}^{1\mathrm{H}}A_Y$ / MHz	−3.04 ± 0.64	−3.01 ± 0.56	−2.83 ± 0.55
${}^{1\mathrm{H}}A_Z$ / MHz	−3.25 ± 0.41	−3.27 ± 0.27	−3.07 ± 0.30

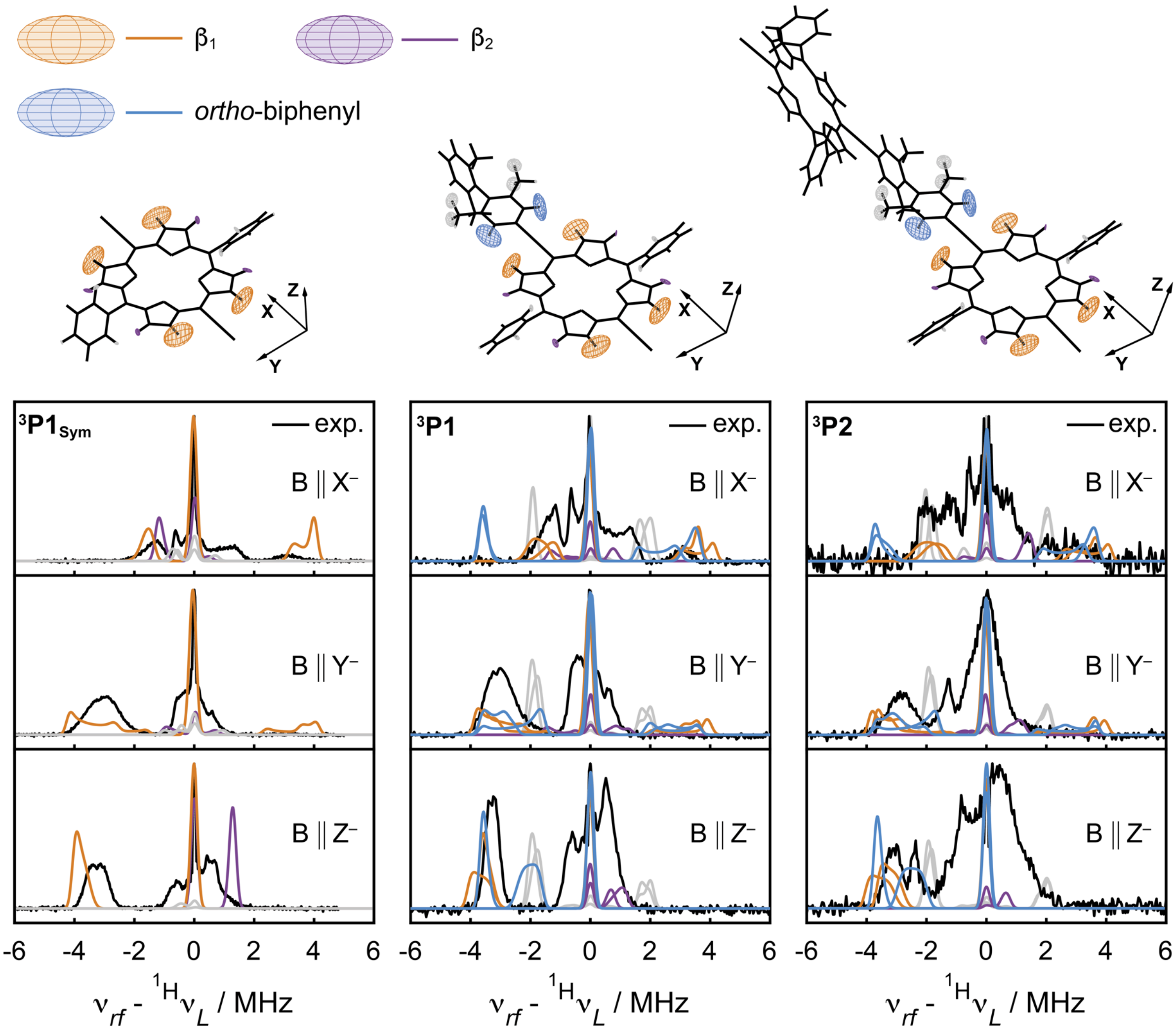

Figure S37. Comparison of the experimental [1]H Mims ENDOR spectra of **[3]P1Sym**, **[3]P1**, and **[3]P2** recorded at the X^-, Y^-, and Z^- field positions at 20 K in 2-methyltetrahydrofuran at X-band frequencies with the simulated ENDOR contributions of individual ^{1}H nuclei. Simulations were performed with anisotropic hyperfine tensors $^{1H}\boldsymbol{A}$ obtained from DFT calculations at the B3LYP/EPR-II level of theory and approximately identical orientations of the zero-field splitting tensors indicated in the schematic visualization at the top. Schematic representation of the DFT calculated hyperfine tensors are shown above the simulations. The tensors of the β_1, β_2, and *ortho*-biphenyl protons that give rise to the highlighted simulated transitions are shown in orange, purple, and blue, respectively; additional tensors and simulated ENDOR contributions are shown in grey as a reference.

10. NMR and Mass Spectra of Novel Compounds

3:

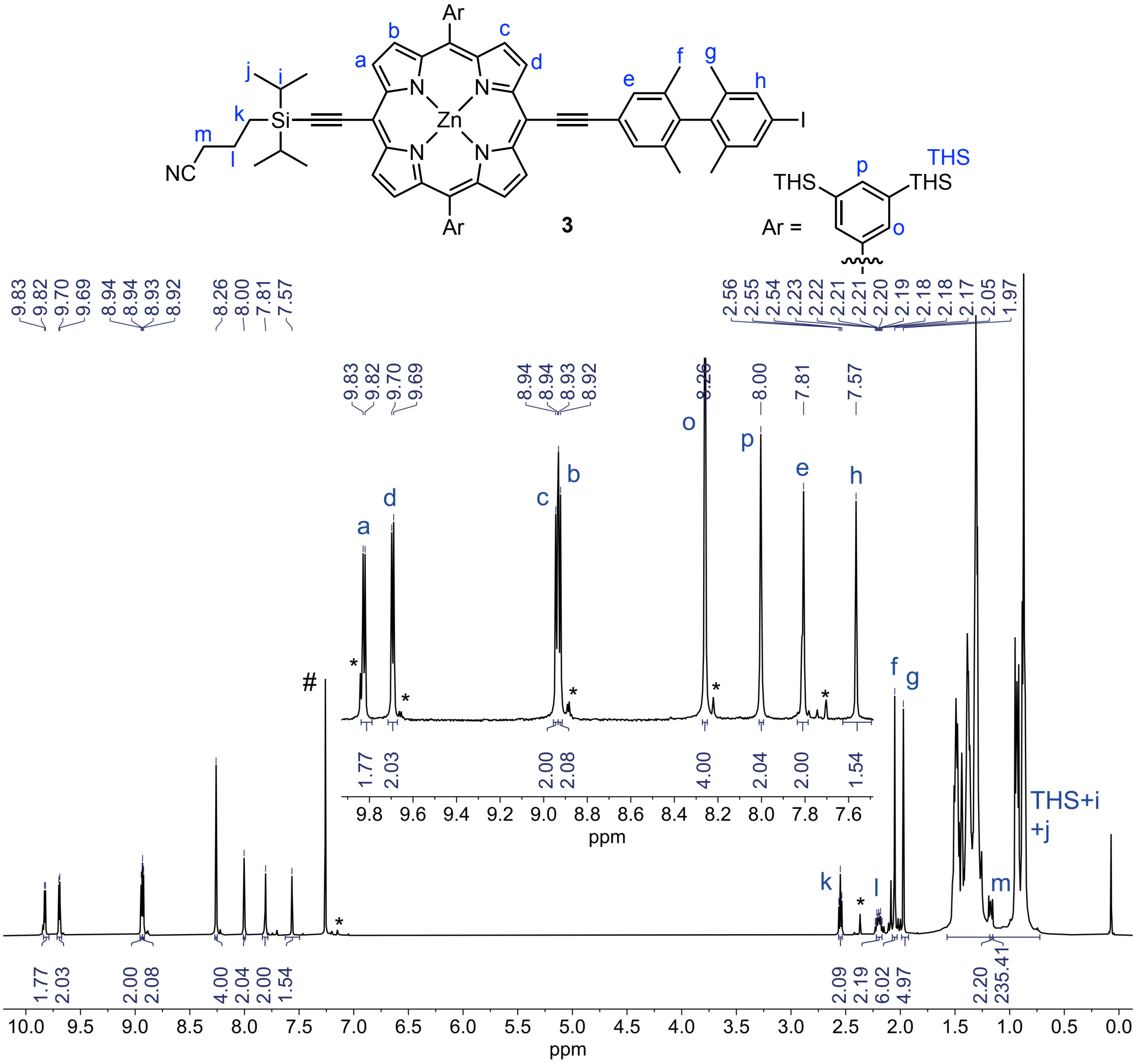

Figure S38. Labeled structure and ^{1}H NMR spectrum of compound **3** (500 MHz, $CDCl_3$, 298 K). # = $CHCl_3$; * = trace impurity.

Table S18. NMR assignment and correlations for **3**.

#	Assign.	^{1}H	Mult. *J* (Hz)	^{1}H–^{1}H COSY	^{1}H–^{1}H NOESY
1	a	9.82 (2H)	d, *J* = 4.6	4	4
2	d	9.69 (2H)	d, *J* = 4.5	3	3
3	c	8.94 (2H)	d, *J* = 4.5	2	2,5
4	b	8.93 (2H)	d, *J* = 4.7	1	1,5
5	o	8.26 (4H)	s	6	3,4
6	p	8.00 (2H)	p	5	14
7	e	7.81 (2H)	s	11	11
8	h	7.57 (2H)	s	12	12
9	k	2.55 (2H)	d, *J* = 6.8	10	10,13
10	l	2.22–2.17 (2H)	m	9, 13	9,13
11	f	2.05 (6H)	f	7	7
12	g	1.97 (6H)	g	8	8
13	m	1.18–1.15 (2H)	m	10	9,10
14	THS+i+j	1.57–0.79 (170H)	m	14	6,14

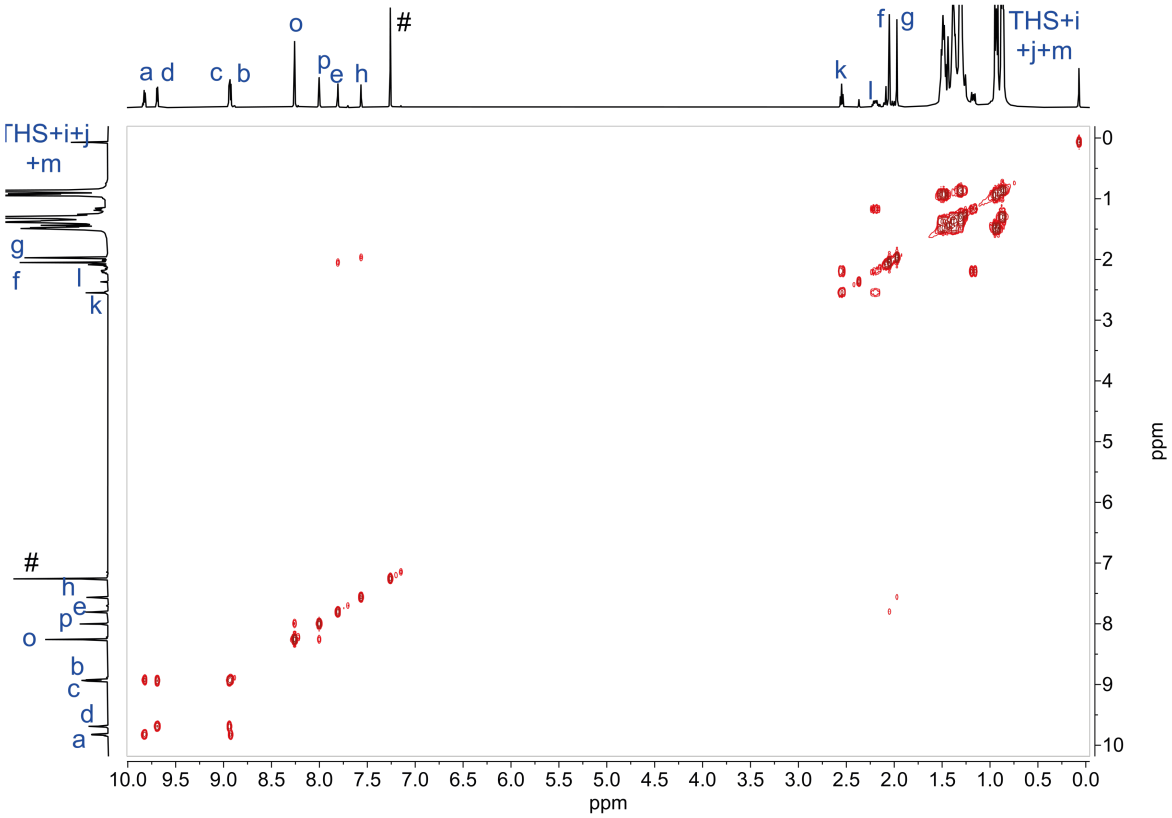

Figure S39. ^{1}H-^{1}H COSY spectrum of **3** (500 MHz, $CDCl_3$, 298 K).

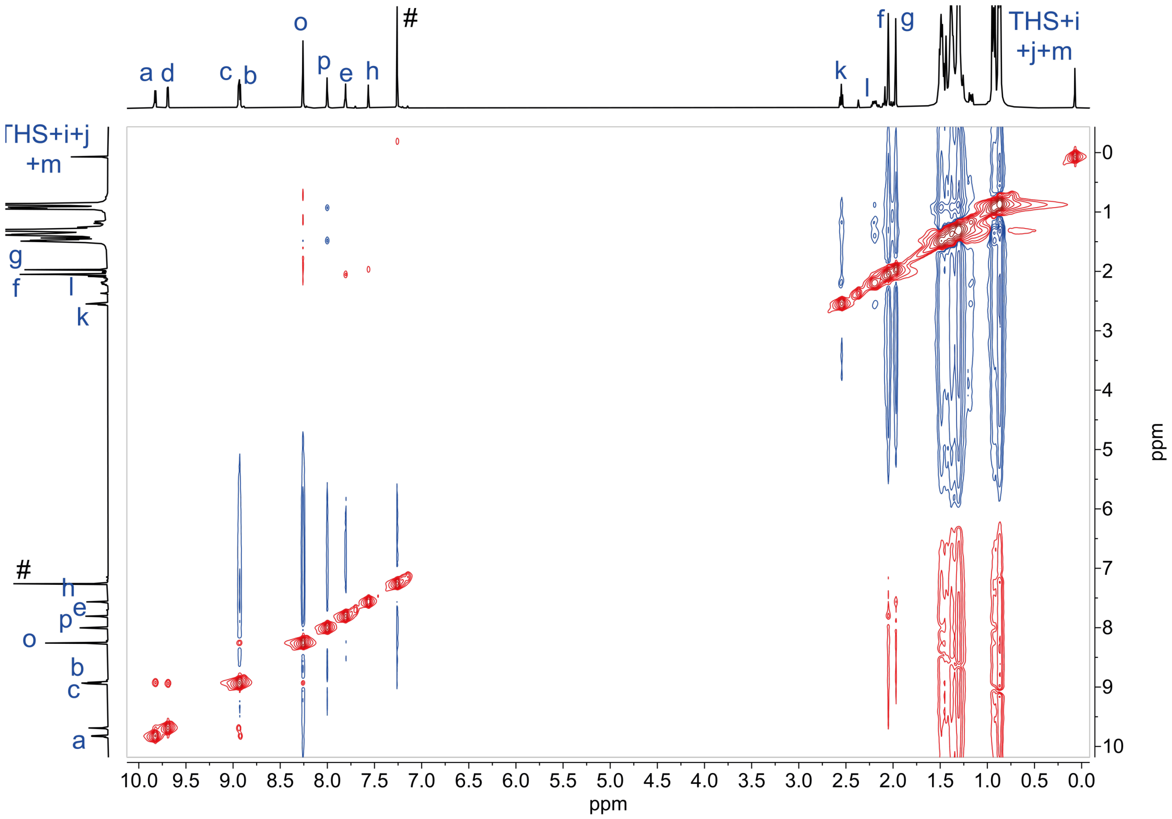

Figure S40. ^{1}H-^{1}H NOESY spectrum of **3** (500 MHz, $CDCl_3$, 298 K).

P1:

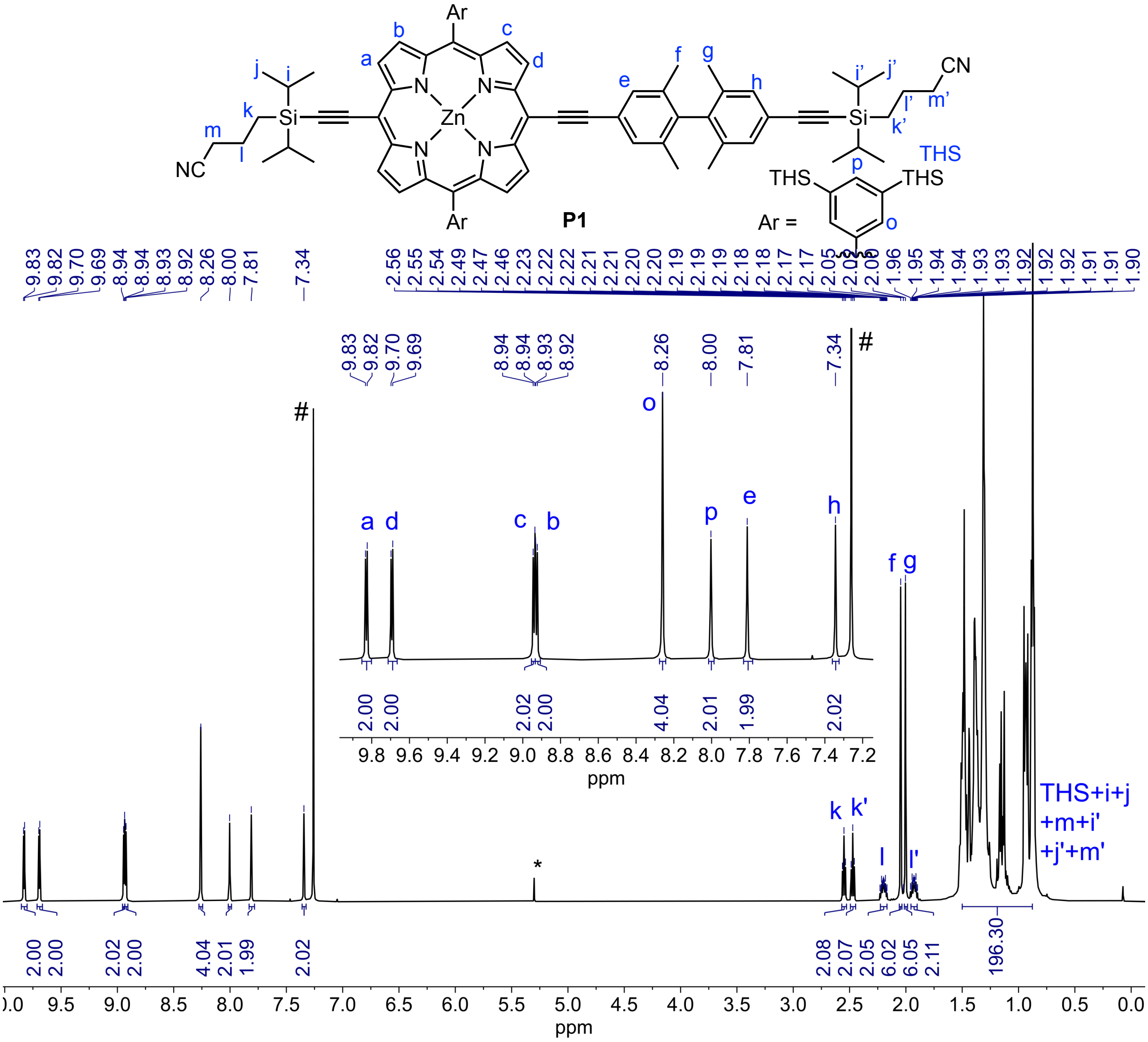

Figure S41. Labeled structure and ^{1}H NMR spectrum of porphyrin monomer **P1** (500 MHz, $CDCl_3$, 298 K). # = $CHCl_3$; * = CH_2Cl_2.

Table S19. NMR assignment and correlations for porphyrin monomer **P1**.

#	Assign.	^{1}H	Mult. *J* (Hz)	^{1}H–^{1}H COSY	^{1}H–^{1}H NOESY
1	a	9.83 (2H)	d, *J* = 4.6	4	4
2	d	9.69 (2H)	d, *J* = 4.6	3	3
3	c	8.94 (2H)	d, *J* = 4.6	2	2,5
4	b	8.93 (2H)	d, *J* = 4.6	1	1,5
5	o	8.26 (4H)	s	6	3,4,15
6	p	8.00 (2H)	s	5	15
7	e	7.81 (2H)	s	12	12
8	h	7.34 (2H)	s	13	13
9	k	2.55 (2H)	t, *J* = 6.8	11	-
10	k'	2.47 (2H)	t, *J* = 7.0	14	-
11	l	2.23–2.17 (2H)	m	9,15	-
12	f	2.05 (6H)	s	7	7
13	g	2.00 (6H)	s	8	8
14	l'	1.96–1.89 (2H)	m	10,15	-
15	THS+i+j+m +i'+j'+m'	1.50–0.88 (188H)	m	11,14,15	5,6,15

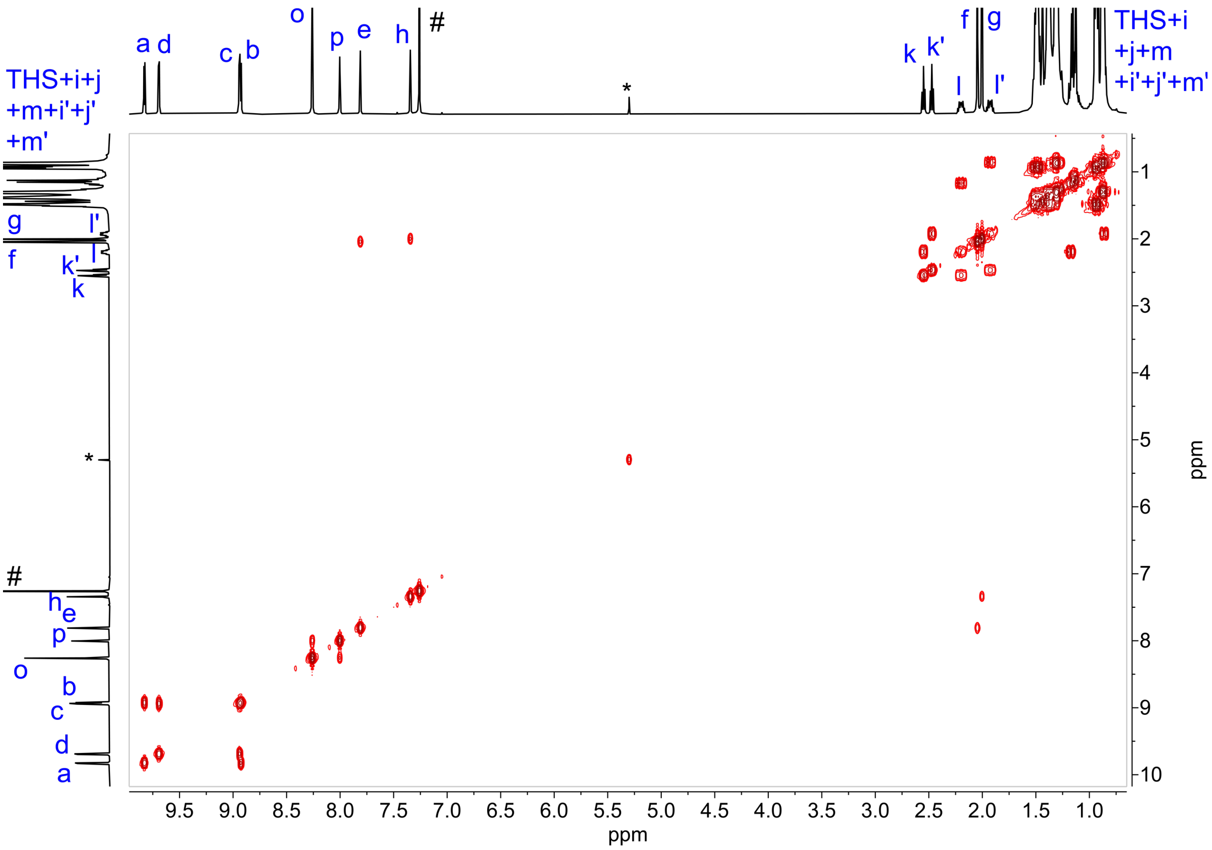

Figure S42. ^{1}H-^{1}H COSY spectrum of monomer **P1** (500 MHz, $CDCl_3$, 298 K).

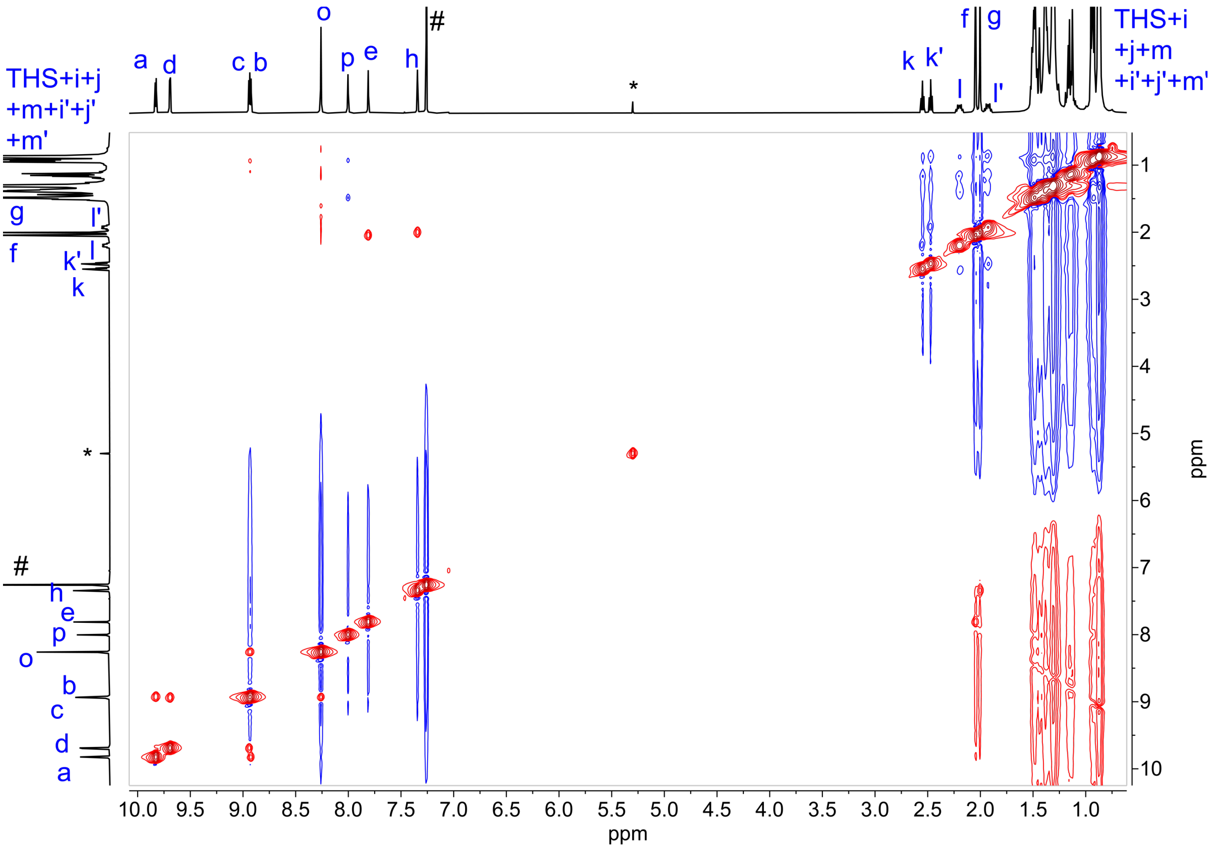

Figure S43. ^{1}H-^{1}H NOESY spectrum of monomer **P1** (500 MHz, $CDCl_3$, 298 K).

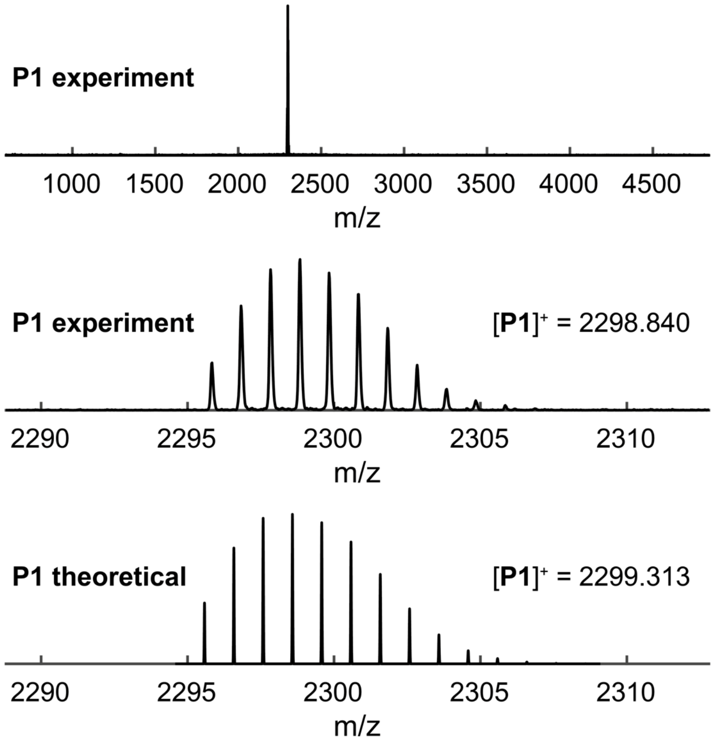

Figure S44. Experimental and simulated MALDI-Tof spectra of **P1**.

P2:

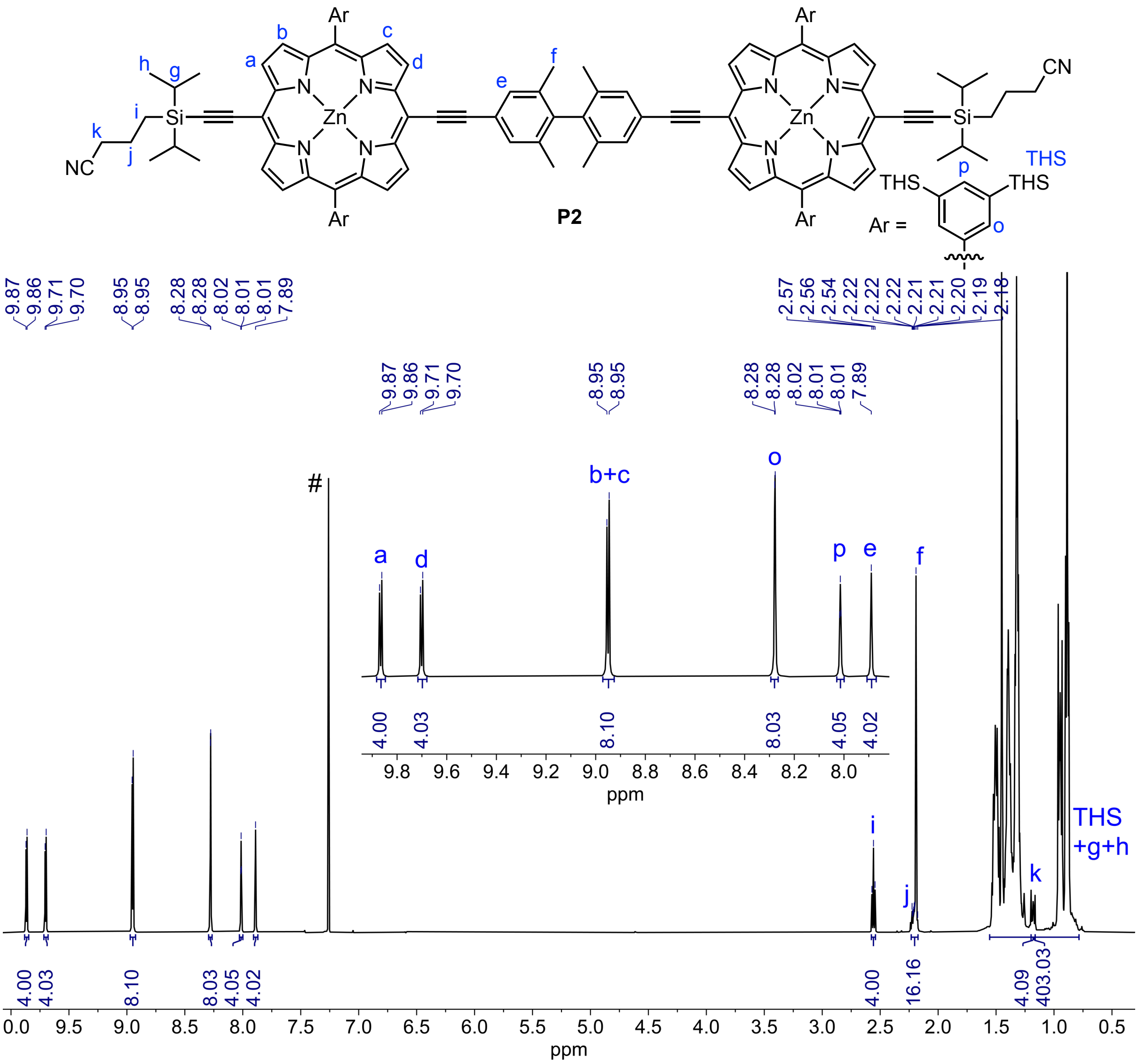

Figure S45. Labeled structure and ^{1}H NMR spectrum of porphyrin dimer **P2** (500 MHz, $CDCl_3$, 298 K). # = $CHCl_3$.

Table S20. NMR assignment and correlations for porphyrin dimer **P2**.

#	Assign.	^{1}H	Mult. *J* (Hz)	^{1}H–^{1}H COSY	^{1}H–^{1}H NOESY
1	a	9.87 (4H)	d, J = 4.6	3	3
2	d	9.70 (4H)	d, J = 4.6	3	3
3	b+c	8.95 (8H)	d, J = 4.6	1,2	1,2,4
4	o	8.28 (8H)	d, J = 1.2	5	3
5	p	8.01 (4H)	d, J = 1.2	4	-
6	e	7.89 (4H)	s	8	8
7	i	2.56 (4H)	t, J = 6.8	8	-
8	j+f	2.24–2.17 (16H)	s+m	6,7,9	6
9	k	1.20–1.16 (4H)	m	8	-
10	THS+g+h	1.55–0.81 (340H)	m	10	10

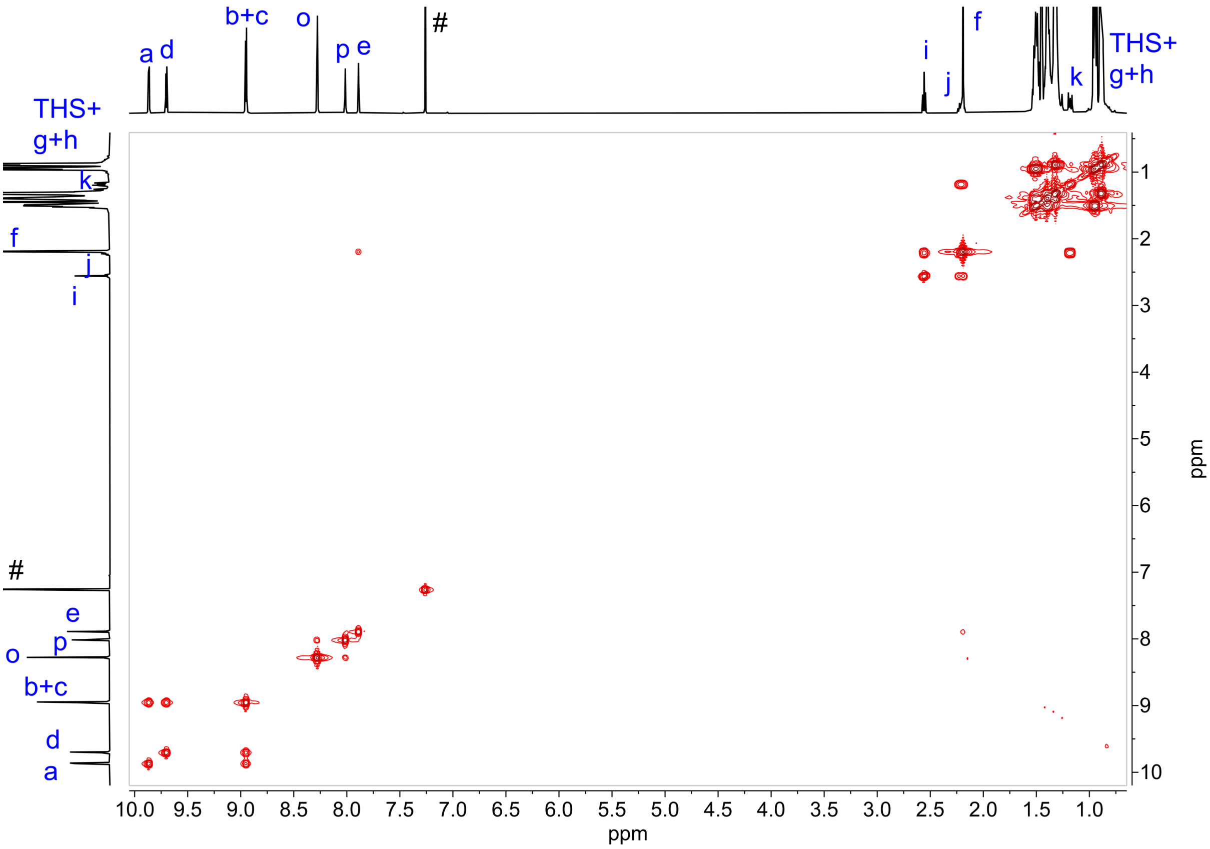

Figure S46. ^{1}H-^{1}H COSY spectrum of dimer **P2** (500 MHz, $CDCl_3$, 298 K).

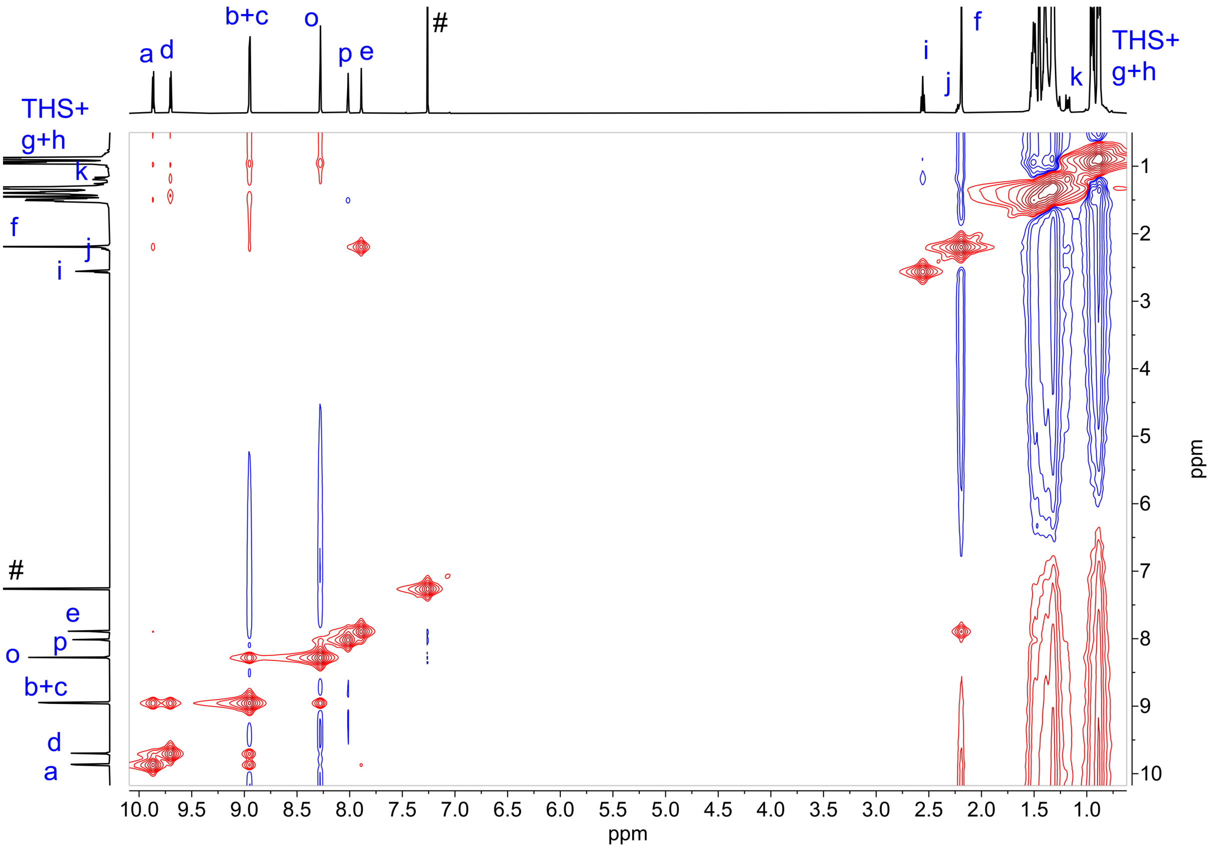

Figure S47. ^{1}H-^{1}H NOESY spectrum of dimer **P2** (500 MHz, $CDCl_3$, 298 K).

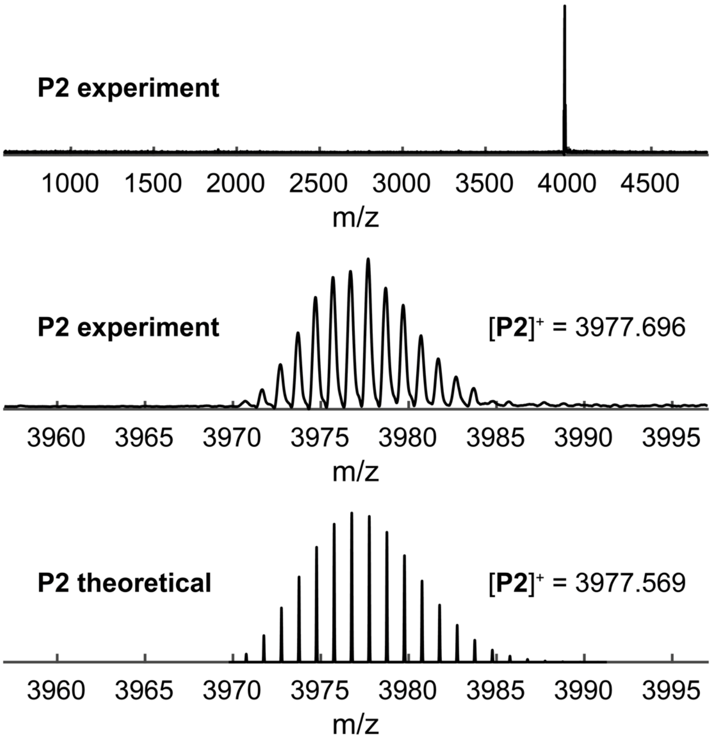

Figure S48. Experimental and simulated MALDI-Tof spectra of **P2**.

11. References

[1] Neese, F. The ORCA program system, *Wiley Interdiscip. Rev.: Comput. Mol. Sci.* **2012**, *2*, 73.

[2] Parkinson, P. *et al.*, *J. Am. Chem. Soc.* **2014**, *136*, 8217–8220.

[3] Paul, S. *et al.*, *J. Phys. Chem. B.* **2021**, *125*, 231–239.

[4] Meyer, A. *et al.*, *Angew. Chem. Int. Ed.* **2020**, *56*, 373–379.

[5] Atherton, N. M., *et al. Chem. Phys. Lett.* **1984**, *103*, 302–304.

[6] McConnell, H. M., *J. Chem. Phys.* **1956**, *24*, 764–766.

[7] Zalibera, M., *J. Phys. Chem. A* **2013**, *117*, 1439–1448.

[8] Kozhanov, K. A., *J. Mol. Struc.* **2017**, *1147*, 541–548.

[9] Stoll, S. *et al.*, *J. Magn. Reson.* **2006**, *178*, 42–55.

[10] Fraenkel, G. K., *J. Phys. Chem.* **1967**, *71*, 139–171.

[11] Freed, J. H., *J. Phys. Chem.* **1963**, *39*, 326.

[12] Bloch, F., *Phys. Rev.* **1946**, *70*, 460.

[13] Gutowski, H. S., *J. Chem. Phys.* **1953**, *21*, 279.

[14] McConnell, H. M., *J. Phys. Chem.* **1958**, *28*, 430.

[15] Newton, M. D. *et al.*, *Ann. Rev. Phys. Chem.* **1984**, *35*, 437–480.

[16] Zhu, G. Y. *et al.*, *Nat. Comm.* **2021**, *12*, 456.

[17] Gajdos, F. *et al.*, *J. Chem. Theory Comput.* **2014**, *10*, 4653–4660.

[18] Cheng, C. Y. *et al.*, *Chem. Sci.* **2020**, *11*, 4922–4933.

[19] Troisi, A. *et al.*, *J. Chem. Phys.* **2003**, *119*, 5782–5788.

[20] Marcus, R. A., *J. Chem. Phys.* **2004**, *24*, 966–978.

[21] Vydrov, O. A. *et al.*, *J. Chem. Phys.* **2006**, *125*.
